# Supplementary material for: Non-Ergot Dopamine Agonists and the Risk of Heart Failure and Other Adverse Cardiovascular Reactions in Parkinson’s Disease
Source: Brain Sci. 2024 Jul 31;14(8):776. doi: 10.3390/brainsci14080776 (PMC11352331; doi:10.3390/brainsci14080776)
Supplement: Supplementary file 1 [file brainsci-14-00776-s001.zip › brainsci-3087225-supplementary.pdf]

Systematic Review: Supplementary Material

Non-ergot dopamine agonists and the risk of heart failure and other adverse cardiovascular reactions in Parkinson's disease

Supplementary Material S1. Differences between protocol and review.

Additional co-authors (MH and PG) were recruited to assist with review completion, including data extraction (JC, YF, MH, and PG completed all screening, data extraction, and risk of bias assessments in duplicate).

Case–control and cohort studies included in our review that only reported counts of events for outcomes of interest were not considered for quantitative synthesis. This was because adjusted effect estimates could not be derived from these data. These studies were, therefore, not assessed using the Newcastle–Ottawa scale nor were related study findings evaluated by GRADE. The data extracted from these studies are presented Supplementary Material S5 (Table S5.1).

We initially planned to report mean differences or standardized mean differences with 95% CIs, as appropriate, for continuous outcomes. After careful consideration of our primary and secondary outcomes, all endpoints were determined to be dichotomous in nature. Therefore, no findings were reported using continuous measures.

We planned to not report the results of meta-analyses in instances where I2 was greater than 50%. Since investigations of heterogeneity are of limited value when there are very few studies, we reported the findings of meta-analyses that included a small number of studies regardless of heterogeneity.

Subgroup analyses by dose of non-ergot DAs were not initially planned; however, they were subsequently determined to be possible and beneficial. These analyses were, therefore completed.

The GRADE approach [1,2] was utilized to assess the quality of evidence for our primary and secondary outcomes. Findings from GRADE assessments are presented in the Summary of Findings tables generated by GRADEpro GDT software for each comparison included in our review.

Supplementary Material S2. Search Strategies.

MEDLINE search strategy via Ovid SP

1. Parkinson disease/
2. parkinson*.tw.
3. (paralysis adj2 agitans).tw.
4. or/1-3
5. Piribedil/
6. Apomorphine/
7. apomorphine.tw.
8. p#ribedil.tw.
9. pramipexole.tw.
10. ropinirole.tw.
11. rotigotine.tw.
12. or/5-11
13. (ae or co or de).fs. or (safe or safety or side effect* or undesirable effect* or treatment emergent or tolerability or toxicity or adrs or (adverse adj2 (effect or effects or reaction or reactions or event or events or outcome or outcomes))).ti,ab.
14. exp Cardiovascular Diseases/
15. exp Cerebrovascular Disorders/
16. Defibrillators, implantable/
17. exp Pacemaker, Artificial/
18. exp Syncope/
19. exp Angiotensin-Converting Enzyme Inhibitors/
20. exp Adrenergic beta-Antagonists/
21. Pleural Effusion/
22. (cardiovasc* adj2 dis*).tw.
23. (cerebrovasc* adj2 dis*).tw.
24. (heart adj2 failure).tw.
25. (edema or oedema).tw.
26. (pleura* adj1 effusion*).tw.
27. (valv* adj2 dis*).tw.
28. regurgitation.tw.
29. hypertension.tw.
30. hypotension.tw.
31. (myocardial adj2 infarction*).tw.
32. (heart adj2 attack*).tw.
33. arrhythmia*.tw.
34. (implant* adj1 cardiovert* adj1 defib*).tw.
35. (cardiac adj1 resynchroni?ation* adj1 therap*).tw.
36. sycop*.tw.
37. stroke.tw.
38. ACE.tw.
39. (beta adj1 blocker*).tw.
40. (cardiovasc* adj2 death*).tw.
41. or/14-40
42. 4 and 12 and 13 and 41

Embase search strategy via Ovid SP

1. Parkinson disease/
2. parkinson*.tw.
3. (paralysis adj2 agitans).tw.
4. or/1-3
5. Piribedil/
6. Apomorphine/
7. apomorphine.tw.
8. ropinirole/
9. pramipexole/
10. rotigotine/
11. p#ribedil.tw.
12. pramipexole.tw.
13. ropinirole.tw.
14. rotigotine.tw.
15. or/5-14
16. DRUG/ae, to or (safe or safety or side effect* or undesirable effect* or treatment emergent or tolerability or toxicity or adrs or (adverse adj2 (effect or effects or reaction or reactions or event or events or outcome or outcomes))).ti,ab.
17. exp Cardiovascular Disease/
18. exp Cerebrovascular Disorder/
19. exp artificial heart pacemaker/
20. exp faintness/
21. exp dipeptidyl carboxypeptidase inhibitor/
22. exp beta adrenergic receptor blocking agent/
23. exp pleural effusion/
24. (cardiovasc* adj2 dis*).tw.
25. (cerebrovasc* adj2 dis*).tw.
26. (heart adj2 failure).tw.
27. (edema or oedema).tw.
28. (pleura* adj1 effusion*).tw.
29. (valv* adj2 dis*).tw.
30. regurgitation.tw.
31. hypertension.tw.
32. hypotension.tw.
33. (myocardial adj2 infarction*).tw.
34. (heart adj2 attack*).tw.
35. arrhythmia*.tw.
36. (implant* adj1 cardiovert* adj1 defib*).tw.
37. (cardiac adj1 resynchroni?ation* adj1 therap*).tw.
38. sycop*.tw.
39. stroke.tw.
40. ACE.tw.
41. (beta adj1 blocker*).tw.
42. (cardiovasc* adj2 death*).tw.
43. or/17-42
44. 4 and 15 and 16 and 43

PsycINFO search strategy via Ovid SP

1. Parkinson's Disease/
2. parkinson*.tw.
3. (paralysis adj2 agitans).tw.
4. or/1-3
5. Apomorphine/
6. apomorphine.tw.
7. p#ribedil.tw.
8. pramipexole.tw.
9. ropinirole.tw.
10. rotigotine.tw.
11. or/5-10
12. exp Cardiovascular disorders/
13. exp Cerebrovascular Disorders/
14. Artificial pacemakers/
15. (cardiovasc* adj2 dis*).tw.
16. (cerebrovasc* adj2 dis*).tw.
17. (heart adj2 failure).tw.
18. (edema or oedema).tw.
19. (pleura* adj1 effusion*).tw.
20. (valv* adj2 dis*).tw.
21. regurgitation.tw.
22. hypertension.tw.
23. hypotension.tw.
24. (myocardial adj2 infarction*).tw.
25. (heart adj2 attack*).tw.
26. arrhythmia*.tw.
27. (implant* adj1 cardiovert* adj1 defib*).tw.
28. (cardiac adj1 resynchroni?ation* adj1 therap*).tw.
29. sycop*.tw.
30. stroke.tw.
31. ACE.tw.
32. (beta adj1 blocker*).tw.
33. (cardiovasc* adj2 death*).tw.
34. or/12-33
35. 4 and 11 and 34

Cochrane Central Register of Controlled Trials search strategy via OvidSP

1. exp Parkinsonian Disorders/
2. parkinson*.tw.
3. (paralysis adj2 agitans).tw.
4. or/1-3
5. Piribedil/
6. Apomorphine/
7. apomorphine.tw.
8. p#ribedil.tw.
9. pramipexole.tw.
10. ropinirole.tw.
11. rotigotine.tw.
12. or/5-11
13. exp Cardiovascular Diseases/
14. exp Cerebrovascular Disorders/
15. Defibrillators, implantable/
16. exp Pacemaker, Artificial/
17. exp Syncope/
18. exp Angiotensin-Converting Enzyme Inhibitors/
19. exp Adrenergic beta-Antagonists/
20. Pleural Effusion/
21. (cardiovasc* adj2 dis*).tw.
22. (cerebrovasc* adj2 dis*).tw.
23. (heart adj2 failure).tw.
24. (edema or oedema).tw.
25. (pleura* adj1 effusion*).tw.
26. (valv* adj2 dis*).tw.
27. regurgitation.tw.
28. hypertension.tw.
29. hypotension.tw.
30. (myocardial adj2 infarction*).tw.
31. (heart adj2 attack*).tw.
32. arrhythmia*.tw.
33. (implant* adj1 cardiovert* adj1 defib*).tw.
34. (cardiac adj1 resynchroni?ation* adj1 therap*).tw.
35. sycop*.tw.
36. stroke.tw.
37. ACE.tw.
38. (beta adj1 blocker*).tw.
39. (cardiovasc* adj2 death*).tw.
40. or/13-39
41. 4 and 12 and 40

Cumulative Index to Nursing and Allied Health search strategy

1. (MH "Parkinsonian Disorders+")
2. parkinson*
3. paralysis agitans
4. or/1-3
5. (MH "Apomorphine")
6. apomorphine
7. piribedil
8. pramipexole
9. ropinirole
10. rotigotine
11. or/5-10
12. (MH "Cardiovascular Diseases+")
13. (MH "Cerebrovascular Disorders+")
14. (MH "Defibrillators, Implantable")
15. (MH "Pacemaker, Artificial")
16. (MH "Syncope+")
17. (MH "Angiotensin-Converting Enzyme Inhibitors+")
18. (MH "Adrenergic Beta-Antagonists+")
19. (MH "Pleural Effusion+")
20. (cardiovasc* N2 dis*)
21. (cerebrovasc* N2 dis*)
22. (MH "Edema")
23. (MH "Heart Failure+")
24. (valv* N2 dis*)
25. edema
26. oedema
27. heart failure
28. (pleura* N1 effusion*)
29. regurgitation
30. hypertension
31. hypotension
32. myocardial infarction*
33. heart attack
34. arrhythmia*
35. implant* cardiovert* defib*
36. cardiac resynchronization therap*
37. cardiac resynchronisation therap*
38. syncop*
39. stroke
40. age
41. beta blocker*
42. (cardiovasc N2 death*)
43. or/12-42
44. 4 and 11 and 43

PubMed search strategy

("parkinson disease"[MeSH Terms] OR parkinson*[Text Word] OR paralysis agitans[Text Word])

AND

("piribedil"[MeSH Terms] OR apomorphine[Text Word] OR piribedil[Text Word] OR pramipexole[Text Word] OR ropinirole[Text Word] OR rotigotine[Text Word])

AND

("cardiovascular diseases"[MeSH Terms] OR "cerebrovascular disorders"[MeSH Terms] OR "defibrillators, implantable"[MeSH Terms] OR "pacemaker, artificial"[MeSH Terms] OR "syncope"[MeSH Terms] OR "angiotensin converting enzyme inhibitors"[MeSH Terms]
OR "adrenergic beta antagonists"[MeSH Terms] OR "pleural effusion"[MeSH Terms] OR "cardiovascular disorder*"[Text Word] OR "cardiovascular disease*"[Text Word] OR "cerebrovascular disorder*"[Text Word] OR "cerebrovascular disease*"[Text Word] OR heart failure[Text Word] OR edema[Text Word] OR oedema[Text Word] OR "pleura* effusion*"[Text Word] OR "valve disorder*"[Text Word] OR "valve disease*"[Text Word]
OR regurgitation[Text Word] OR hypertension[Text Word] OR hypotension[Text Word] OR myocardial infarction*[Text Word] OR heart attack*[Text Word] OR arrhythmia*[Text Word] OR "implantable cardioverter defibrillator*"[Text Word] OR cardiac resynchronization therap*[Text Word] OR cardiac resynchronisation therap*[Text Word] OR syncop*[Text Word] OR stroke[Text Word] OR ACE[Text Word] OR beta blocker*[Text Word] OR "cardiovascular death*"[Text Word])

Supplementary Material S3. Newcastle–Ottawa quality assessment scale for observational studies.

NEWCASTLE - OTTAWA QUALITY ASSESSMENT SCALE CASE CONTROL STUDIES
Note: A study can be awarded a maximum of one star for each numbered item within the Selection and Exposure categories. A maximum of two stars can be given for Comparability.

Selection
1) Is the case definition adequate?
- a) yes, with independent validation *
- b) yes, eg record linkage or based on self reports
- c) no description

2) Representativeness of the cases
- a) consecutive or obviously representative series of cases *
- b) potential for selection biases or not stated

3) Selection of Controls
- a) community controls *
- b) hospital controls
- c) no description

4) Definition of Controls
- a) no history of disease (endpoint) *
- b) no description of source

Comparability
1) Comparability of cases and controls on the basis of the design or analysis
- a) study controls for _______________ (Select the most important factor.) *
- b) study controls for any additional factor * (This criteria could be modified to indicate specific control for a second important factor.)

Exposure
1) Ascertainment of exposure
- a) secure record (eg surgical records) *
- b) structured interview where blind to case/control status *
- c) interview not blinded to case/control status
- d) written self report or medical record only
- e) no description

2) Same method of ascertainment for cases and controls
- a) yes *
- b) no

3) Non-Response rate
- a) same rate for both groups *
- b) non respondents described
- c) rate different and no designation

NEWCASTLE - OTTAWA QUALITY ASSESSMENT SCALE COHORT STUDIES

Note: A study can be awarded a maximum of one star for each numbered item within the Selection and Outcome categories. A maximum of two stars can be given for Comparability

Selection

1) <u>Representativeness of the exposed cohort</u>
    a) truly representative of the average _______________ (describe) in the community *
    b) somewhat representative of the average ______________ in the community *
    c) selected group of users eg nurses, volunteers
    d) no description of the derivation of the cohort

2) <u>Selection of the non exposed cohort</u>
    a) drawn from the same community as the exposed cohort *
    b) drawn from a different source
    c) no description of the derivation of the non exposed cohort

3) <u>Ascertainment of exposure</u>
    a) secure record (eg surgical records) *
    b) structured interview *
    c) written self report
    d) no description

4) <u>Demonstration that outcome of interest was not present at start of study</u>
    a) yes *
    b) no

Comparability

1) <u>Comparability of cohorts on the basis of the design or analysis</u>
    a) study controls for _____________ (select the most important factor) *
    b) study controls for any additional factor * (This criteria could be modified to indicate specific control for a second important factor.)

Outcome

1) <u>Assessment of outcome</u>
    a) independent blind assessment *
    b) record linkage *
    c) self report
    d) no description

2) <u>Was follow-up long enough for outcomes to occur</u>
    a) yes (select an adequate follow up period for outcome of interest) *
    b) no

3) <u>Adequacy of follow up of cohorts</u>
    a) complete follow up - all subjects accounted for *
    b) subjects lost to follow up unlikely to introduce bias - small number lost - > ____ % (select an adequate %) follow up, or description provided of those lost) *
    c) follow up rate < ____% (select an adequate %) and no description of those lost
    d) no statement

Supplementary Material S4. Characteristics of included studies.

Study	Methods	Participants	Interventions	Outcomes
Barone, 2010 [3]	**Study type:** double-blind RCT<br><br>**Duration:** 12 weeks	**Setting:** multicentre (76 centres in 12 European countries and South Africa)<br><br>**Number of participants:** 296 randomized (placebo = 152; pramipexole = 144)<br><br>**Sex:** % female: placebo = 49%; pramipexole = 57%<br><br>**Age:** mean (SD) in years: placebo = 66.6 (9.9); pramipexole = 67.4 (9.0)<br><br>**PD duration:** mean (SD) in years: placebo = 4.0 (3.9); pramipexole = 4.0 (4.5)<br><br>**Inclusion criteria:**<br>• at least 30 years old<br>• had idiopathic PD<br>• did not have motor fluctuations<br>• required to have clinically relevant depressive symptoms<br><br>**Exclusion criteria:**<br>• a score of less than 24 on the Mini-Mental State Examination<br>• severe depression, as defined by the presence of suicidal ideation<br>• present psychotherapy<br>• use of typical neuroleptics, meto-clopramide, α-methyldopa, methylphenidate, reserpine, flunarizine,	**Control:** placebo<br><br>**Primary treatment:** pramipexole (0.125-1 mg/dose, 3 times a day)	• orthostatic hypotension

Study	Methods	Participants	Interventions	Outcomes
		cinnarizine, or amphetamine derivatives within the past 3 months<br>• a history of malignant melanoma<br>• previous deep brain stimulation surgery<br>• women of childbearing potential were excluded if they were pregnant, lactating, or not taking adequate contraception		
Blindeauer, 2003 [4]	**Study type:** double-blind, parallel-design RCT<br><br>**Duration:** 11 weeks	**Setting:** multicentre<br><br>**Number of participants:** 242 randomized (placebo = 47; rotigotine 4.5 mg = 49; rotigotine 9.0 mg = 47; rotigotine 13.5 mg = 48; rotigotine 18.0 mg = 51)<br><br>**Sex:** % female: placebo = 51.1%; rotigotine 4.5 mg = 30.6%; rotigotine 9.0 mg = 23.4%; rotigotine 13.5 mg = 35.4%; rotigotine 18.0 mg = 41.2%<br><br>**Age:** mean age (SD) in years: placebo = 62.3 (10.5); rotigotine 4.5mg = 61.8 (9.8); rotigotine 9.0 mg = 60.9 (8.3); rotigotine 13.5 mg = 61.3 (10.9); rotigotine 18.0 mg = 60.5 (10.7)<br><br>**PD duration:** mean (SD) in years: placebo = 1.3 (1.4); rotigotine 4.5 mg = 1.2 (1.4); rotigotine 9.0 mg = 1.5 (2.0); rotigotine 13.5 mg = 1.2 (1.0); rotigotine 18.0 mg = 1.1 (1.2)<br><br>**Inclusion criteria:**<br>• men and women older than 30<br>• diagnosed with idiopathic PD	**Treatment groups:**<br>• rotigotine (grouped in doses of 4.5 mg/24 hr, 9.0 mg/24 hr, 13.5 mg/24 hr, 18.0 mg/24 hr)<br>• placebo	• peripheral edema

Study	Methods	Participants	Interventions	Outcomes
		• had an H&Y stage of 3 or less<br><br>**Exclusion criteria:**<br>• had cognitive impairment<br>• were unable to appropriately apply and remove the patches<br>• had a history of skin sensitivity to adhesives or other transdermal medications<br>• had taken a dopamine agonist or levodopa within 28 days of the baseline visit or had ever taken levodopa for longer than 6 months<br>• had an atypical Parkinsonian syndrome<br>• had a clinically unstable medical or psychiatric condition<br>• had cardiac abnormalities such as arrhythmias, conduction blocks, congestive heart failure, QT-corrected interval of 500 milliseconds or more, unexplained syncope, symptomatic orthostatic hypotension, or a recent myocardial infarction<br>• had recent exposure to monoamine oxidase type A inhibitors, amphetamines, dopamine-depleting antihypertensive agents, neuroleptics, or antipsychotics or antiemetics that blocked central dopamine activity		
Castro-Caldas, 2006 [5]	**Study type:** double-blind, parallel-design RCT	**Setting:** multicentre (5 centres in the UK, 16 in Belgium, 44 in France, 15 in Spain, 10 in Germany, 6 in Italy, 5 in Argentina, 4 in Portugal)	**Treatment groups:**<br>• piribedil (50 mg/dose, 1-3 times/24hr)	• hypotension<br>• hypertension<br>• syncope<br>• peripheral edema

Study	Methods	Participants	Interventions	Outcomes
	**Duration:** 12 months	**Number of participants:** 425 randomized (piribedil = 210; bromocriptine = 215)<br><br>**Sex:** % female: piribedil = 44.3%; bromocriptine = 44.7%<br><br>**Age:** mean (SD) in years: piribedil = 64.3 (7.6); bromocriptine = 65.1 (7.9)<br><br>**PD duration:** mean (SD) in months: piribedil = 37 (24); bromocriptine = 39 (29)<br><br>**Inclusion criteria:**<br>• male and female patients<br>• 40 to 77 years of age<br>• a clinical diagnosis of idiopathic PD, stages I to III on the H&Y scale were recruited<br>• their motor symptoms, with or without fluctuations, had to be insufficiently controlled and they had to require therapeutic adaptation<br><br>**Exclusion criteria:**<br>• patients frequently falling<br>• previous neurosurgery for PD<br>• patients suffering from psychotic symptoms or visual hallucinations<br>• patients with intellectual impairment	• bromocriptine (1.25-25 mg dose, 1-3 times/24hr)	

Study	Methods	Participants	Interventions	Outcomes
		• severe cardiovascular diseases, including uncontrolled coronary ischemic heart disease, recent acute myocardial infarction, past history of symptomatic orthostatic hypotension, unexplained loss of consciousness<br>• uncontrolled hypertension within the past 2 months<br>• cancer of any type, severe or uncontrolled diabetes, renal or hepatic disease, gastric or duodenal ulcer<br>• past history of significant psychiatric disease or current major depressive episode		
Crispo, 2016 [6]	**Study type:** case–control<br><br>**Duration:** 13 years, 2000-2012	**Setting:** USA, Cerner Corporation Health Facts database<br><br>**Number of participants:** 14,122<br><br>**Sex:** 46% female<br><br>**Age:** 40-90+ years of age<br><br>**Inclusion criteria:**<br>• hospitalized individuals with a diagnosis of PD who were prescribed an anti-Parkinson's drug during their encounter<br><br>**Exclusion criteria:**<br>• diagnoses (in any setting) of secondary Parkinsonism or other degenerative diseases of the basal ganglia	**Control:** no treatment or levodopa monotherapy<br><br>**Treatment groups:**<br>• pramipexole<br>• ropinirole	• myocardial infarction<br>• heart failure<br>• hypotension<br>• valvulopathy<br>• ischemic stroke

Study	Methods	Participants	Interventions	Outcomes
		• those under the age of 40 years or without a documented age at first PD diagnosis<br>• missing or unknown demographic (sex and/or race) data<br>• only outpatient encounters<br>• only inpatient encounters occurring before their PD diagnosis<br>• encounters where the inpatient stay was less than 3 days due to discharge or death		
Grosset, 2013 [7]	**Study type:** double-blind, parallel-design RCT<br><br>**Duration:** 17 weeks	**Setting:** multicentre (16 centres in 3 countries)<br><br>**Number of participants:** 55 randomized (placebo = 15; apomorphine = 40)<br><br>**Sex:** % female: placebo = 46.7%; apomorphine = 50%<br><br>**Age:** mean (SD) in years): placebo = 65.8 (5.7); apomorphine = 65.6 (7.7)<br><br>**PD duration:** mean (SD) in years: placebo = 11.5 (3.2); apomorphine = 12.2 (3.9)<br><br>**Inclusion criteria:**<br>• patients ages 30 to 90 years with a clinical diagnosis of PD for at least 5 years<br>• fulfilling the UK Brain Bank Criteria<br>• at H&Y stage 2–4 in the 'on' state<br>• motor fluctuations<br>• minimum of 2 hours average daily 'off' time and with at least a 30% improvement in unified PD rating scale motor score	**Control:** placebo<br><br>**Treatment:** apomorphine (1.5-4.5 mg/dose)	• hypertension<br>• hypotension

Study	Methods	Participants	Interventions	Outcomes
		(UPDRS 3) in response to an levodopa dose given when 'off'<br><br>**Exclusion criteria:**<br>• not specified		
Guttman 1997 [8]	**Study type:** double-blind, parallel-design RCT<br><br>**Duration:** 9 months, 11 days	**Setting:** multicentre (34 centres in 6 European countries and Canada)<br><br>**Number of participants:** 247 randomized (placebo = 83; pramipexole = 79; bromocriptine = 84)<br><br>**Sex:** % female: placebo = 36.1%; pramipexole = 39.2%; bromocriptine = 34.5%<br><br>**Age:** mean (SD) in years: placebo = 63.72 (10.35); pramipexole = 62.89 (10.03); bromocriptine = 61.51 (9.48)<br><br>**PD duration:** mean (SD) in years: placebo = 7.58 (0.83-23); pramipexole = 6 (0.67-36); bromocriptine = 7.17 (1-23)<br><br>**Inclusion criteria:**<br>• men or women who were at least 30 years old<br>• idiopathic PD with H&Y stages I to IV during an “on” period<br><br>**Exclusion criteria:**<br>• subjects with atypical Parkinsonism<br>• dementia that could impair participation in the study	**Control:** placebo<br><br>**Treatment groups:**<br>• pramipexole (0.375-4.5 mg/24 hr)<br>• bromocriptine (1.25-30 mg/24 hr)	• orthostatic hypotension

Study	Methods	Participants	Interventions	Outcomes
		• psychosis except what was elicited by treatment with levodopa or dopamine agonists<br>• history of a seizure within 2 years<br>• clinically significant heart, liver, or kidney disease<br>• elevation in either total bilirubin, alkaline phosphatase, lactic dehydrogenase, aspartate aminotransferase, or serum creatinine of more than 1.5 times the laboratory normal<br>• retinopathic pigmentosa<br>• presence of active neoplastic disease<br>• surgery within 180 days of the baseline visit that would negatively impact the subject's participation<br>• stereotactic brain surgery<br>• if at the screening visit subjects had a supine systolic blood pressure of less than 100 mmHg or evidence of a symptomatic drop of 20 mmHg or more, measured at 1 minute after standing, they were not permitted to participate<br>• treatment with the following drugs during the month prior to administration of the trial medication was not permitted: alpha methyldopa, flunarizine, cinnarizine, parenteral ergot preparations, bromocriptine, pergolide, lisuride, monoamine oxidase inhibitors except deprenyl, methylphenidate hydrochloride, amphetamine derivatives, and beta blockers (unless used to treat		

Study	Methods	Participants	Interventions	Outcomes
		Parkinsonian symptoms)—or treatment with neuroleptics or metoclopramide during the 2 months prior to administration of the trial medication<br>• females of childbearing potential not using a medically recognized means of contraception were not permitted to participate<br>• subjects who had electroconvulsive therapy within 90 days prior to administration of study medication were not included, as well as subjects participating in other studies of other investigational drugs within 30 days of baseline		
Holloway, 2004 [9]	**Study type:** double-blind, parallel-design RCT<br><br>**Duration:** 4 years	**Setting:** multicentre (17 centres in the USA, 5 centres in Canada)<br><br>**Number of participants:** 301 randomized (levodopa-completed trial = 100; pramipexole-completed trial = 83; levodopa-withdrew trial = 50; pramipexole-withdrew trial = 68)<br><br>**Sex:** % female: levodopa-completed trial = 32%; pramipexole-completed trial = 39.8%; levodopa-withdrew trial = 38%; pramipexole-withdrew trial = 32.3%<br><br>**Age:** mean (SD) in years: levodopa-completed trial = 60.8 (9.8); pramipexole-completed trial = 61.1 (9.6); levodopa-withdrew trial = 61.0 (11.9); pramipexole-withdrew trial = 62.1 (10.8)	**Control:** carbidopa/levodopa (12.5/50 or 25/100 mg per dose, 3 times/24 hr)<br><br>**Primary treatment:** pramipexole (1.5-45 mg/dose 3 times/24 hr)	• peripheral edema

Study	Methods	Participants	Interventions	Outcomes
		**PD duration:** mean (SD) in years: levodopa-completed trial = 1.8 (1.7); pramipexole-completed trial = 1.4 (1.3); levodopa-withdrew trial = 1.8 (1.7); pramipexole-withdrew trial = 1.6 (1.6)<br><br>**Inclusion criteria:**<br>• Parkinson's disease of less than 7 years duration from diagnosis with at least two of the following three cardinal signs: resting tremor, bradykinesia, rigidity being present, without any other known or suspected cause of Parkinsonism, and requiring additional therapy to treat symptoms at the time of enrollment<br>• no use of levodopa or dopamine agonist medications (such as bromocriptine, pergolide) in the past 2 months<br>• modified H&Y scale stages I–III<br>• age 30 years or older<br>• gender: men and women<br><br>**Exclusion criteria:**<br>• previous experience of a dopaminergic complication (such as wearing off, on–off effects, dyskinesias)<br>• previous history of freezing		
Hubble, 1995 [10]	**Study type:** double-blind, parallel-design RCT	**Setting:** 4 centres<br><br>**Number of participants:** 55 randomized (placebo = 27; pramipexole = 28)	**Control:** placebo<br><br>**Treatment:** pramipexole (4.5 mg/24 hr)	• orthostatic hypertension (symptomatic and asymptomatic)

Study	Methods	Participants	Interventions	Outcomes
	**Duration:** 9 weeks	**Sex:** % female: placebo = 55.6%; pramipexole = 71.4%<br><br>**Age:** mean(SD) in years: placebo: 63 (8.8); pramipexole: 63.5 (12.3)<br><br>**PD duration:** mean (SD) in years: placebo = 2.4 (2.4); pramipexole = 2.1 (2.5)<br><br>**Inclusion criteria:**<br>• 21 years of age or older<br>• diagnosis of early idiopathic PD<br><br>**Exclusion criteria:**<br>• evidence of atypical Parkinson's syndromes, clinically significant cardiovascular, cerebrovascular conditions, or other unstable medical condition		
Im, 2003 [11]	**Study type:** parallel-design RCT<br><br>**Duration:** 16 weeks	**Setting:** Asan Medical Center in Seoul, South Korea<br><br>**Number of participants:** 76 randomized (bromocriptine = 39; ropinirole = 37)<br><br>**Sex:** % female: bromocriptine = 49%; ropinirole = 43%<br><br>**Age:** mean (SD) in years: bromocriptine = 60.0 (8.3); ropinirole = 63.5 (10.8)	**Treatment groups:**<br>• bromocriptine (10-17.5 mg/24 hr)<br>• ropinirole (4.5-9.0 mg/24 hr)	• orthostatic hypotension

Study	Methods	Participants	Interventions	Outcomes
		**PD duration:** mean (SD) in months: bromocriptine = 77.2 (38.2); ropinirole = 81.3 (45.3)<br><br>**Inclusion criteria:**<br>• patients were aged over 40 years<br>• had a clinical diagnosis of PD<br><br>**Exclusion criteria:**<br>• had severe disabling peak dose or diphasic dyskinesias and/or complex 'on-off' phenomena<br>• severe systemic or psychiatric disease<br>• history of alcoholism or drug dependence<br>• severe dementia<br>• severe dizziness or fainting as a result of postural hypotension<br>• other clinically relevant abnormalities in their history or diagnostic laboratory tests, including electrocardiography<br>• had previously been treated with ropinirole or had contraindications to bromocriptine or other ergot alkaloids<br>• women of childbearing age were excluded unless they were postmenopausal, surgically sterilised, or had undergone hysterectomy		
Junghanns, 2007 [12]	**Study type:** cohort<br><br>**Duration:** January to December 2005	**Setting:** single-centre<br><br>**Number of participants:** 123 (placebo = 38; peroglide = 25; cabergoline = 24; ropinirole = 13; pramipexole = 23)	**Controls:** no treatment<br><br>**Treatment groups:**<br>• pergolide (3.2 mg/24 hr average dose)	• valvulopathy (aortic regurgitation, mitral regurgitation, valvular heart disease)

Study	Methods	Participants	Interventions	Outcomes
		**Sex:** % female: placebo = 25 (66); peroglide = 22 (88); cabergoline = 16 (67); ropinirole = 7 (54); pramipexole = 15 (65)<br><br>**Age:** mean (SD) in years: placebo = 63.0 (6.8); peroglide = 63.0 (7.6); cabergoline = 64.3 (8.8); ropinirole = 59.9 (7.0); pramipexole = 62.3 (10.6)<br><br>**PD duration:** mean (SD) in months: peroglide = 11.1 (4.9); cabergoline = 8.6 (6.0); ropinirole = 7.3 (2.7); pramipexole = 8.2 (5.2)<br><br>**Inclusion criteria:**<br>• patients with PD taking pergolide, cabergoline, ropinirole, or pramipexole for at least 9 months<br>• female/male outpatients with definite PD and healthy controls both at least 18 years of age<br><br>**Exclusion criteria:**<br>• nonpermitted medications<br>• history of significant coronary heart disease<br>• impaired function/dilatation of left/right ventricle<br>• history of peripheral artery occlusive disease<br>• carcinoid syndrome<br>• any clinically significant illnesses that interfere with capability to participate in the study	• cabergoline (3.9 mg/24 hr average dose)<br>• ropinirole (3.9 mg/24 hr average dose)<br>• pramipexole (8.4 mg/24 hr average dose)	

Study	Methods	Participants	Interventions	Outcomes
		• limited legal capacity<br>• pregnancy and/or lactation period		
Katzenschlager, 2018<br>[13]	**Study type:** double-blind RCT<br><br>**Duration:** 12 weeks	**Setting:** multicentre (23 European hospitals)<br><br>**Number of participants:** 107 randomized (placebo = 54; apomorphine = 53);<br><br>**Sex:** % female: placebo = 40%; apomorphine = 36%<br><br>**Age:** <65 years: placebo = 55%; apomorphine = 49%<br><br>**PD duration:** mean (SD) years: placebo = 10.6 (4.3); apomorphine = 11.8 (5.6)<br><br>**Inclusion criteria:**<br>• at least 30 years old<br>• diagnosed with PD for more than 3 years<br>• had levodopa-related motor fluctuations that had not been adequately controlled by optimised medical treatment<br>• H&Y stage 3 or less in the on state and 2–5 in the off state<br>• had been on the same dose of oral medication for 4 weeks or more before enrolment<br>• able to differentiate between their subjective on and off states and between on with troublesome or non-troublesome dyskinesia and on without dyskinesia	**Control:** placebo<br><br>**Primary treatment:** apomorphine infusion (3-8 mg/hr, approximately 16 hrs per day)	• hypotension<br>• myocardial infarction<br><br>**Note:** investigators reported a single myocardial infarction event that was unrelated to treatment

Study	Methods	Participants	Interventions	Outcomes
		• had to have a mean of 3 hours or more off time per day for 2 days and no day with less than 2 hours off time<br><br>**Exclusion criteria:**<br>• secondary and atypical Parkinsonian syndromes<br>• previous neurosurgical treatment for PD<br>• previous use of apomorphine infusion<br>• treatment during the 28 days before enrolment with apomorphine injections, intrajejunal levodopa, or any neuroleptic drug<br>• had severe freezing of gait leading to falls during on times; clinically relevant postural instability during on times; or symptomatic, clinically relevant uncontrolled orthostatic hypotension, prolonged QT duration, clinically relevant cognitive decline, or at least moderate psychosis during the year before or at enrolment		
Kieburtz, 2011 [14]	**Study type:** RCT<br><br>**Duration:** 12 weeks	**Setting:** multicentre (39 centres in the USA)<br><br>**Number of participants:** 311 randomized (placebo = 77; pramipexole: 0.50 mg BID = 81, 0.75 mg BID = 73; 0.50 mg TID = 80)<br><br>**Sex:** % female: placebo = 75.3%; pramipexole: 0.50 mg BID = 63%; 0.75 mg BID = 56.2%; 0.50 mg TID = 71.3%	**Control:** placebo<br><br>**Treatment:** pramipexole (in groups of 0.5 mg BID, 0.75 mg BID, and 0.5 mg TID)	• peripheral edema

Study	Methods	Participants	Interventions	Outcomes
		**Age:** mean (SD) in years: placebo = 61.2 (11.0); pramipexole: 0.50 mg BID = 62.1 (10.2); 0.75 mg BID = 63.6 (9.9); 0.50 mg TID = 64.1 (9.8)<br><br>**PD duration:** mean (SD) in years: placebo = 1.1 (1.5); pramipexole: 0.50 mg BID = 1.1 (1.3); 0.75 mg BID = 1.2 (1.4); 0.50 mg TID = 1.1 (1.3)<br><br>**Inclusion criteria:**<br>• a diagnosis of idiopathic PD of < 7 years duration characterized by two of three cardinal signs (resting tremor, bradykinesia, and rigidity) that overall needed to be asymmetric<br>• older than 30 years<br>• have a modified H&Y stage < 3<br>• able to tolerate placebo for up to 12 weeks after baseline<br><br>**Exclusion criteria:**<br>• a diagnosis or signs and symptoms of other Parkinsonian syndromes<br>• use of dopaminergic medications within the last 3 months or for longer than 6 months at any time prior to the baseline visit<br>• DSM- IV diagnosis of dementia or Mini-Mental State Examination score of < 26<br>• major depression, active epilepsy within the past year, myocardial infarction within the past 6 months, third-degree heart block or sick sinus syndrome, congestive heart failure Class III or IV,		

Study	Methods	Participants	Interventions	Outcomes
		symptomatic orthostatic hypotension, prior stereotaxic brain surgery, or clinically significant kidney or liver disease		
Korczyn, 1999 [15]	**Study type:** double-blind RCT<br><br>**Duration:** 3 years	**Setting:** multicentre (37 centres in Israel, Europe, and South Africa)<br><br>**Number of participants:** 335 randomized (ropinirole = 168; bromocriptine = 167)<br><br>**Sex:** % female: ropinirole = 39.3%; bromocriptine = 38.9%<br><br>**Age:** mean (SD) in years: ropinirole = 63.0 (10.3); bromocriptine = 62.7 (10.4)<br><br>**PD duration:** mean (SD) in months ropinirole = 22.8 (21.1); bromocriptine = 26.8 (25.5)<br><br>**Inclusion criteria:**<br>• 30 years of age or older and had early idiopathic PD (H&Y stages I-III) requiring dopaminergic therapy<br>• levodopa or dopamine agonists could not have been administered for longer than 6 weeks, and any such treatment was to be discontinued at least 2 weeks before entry to the study<br><br>**Exclusion criteria:**<br>• had severe systemic or psychiatric disease, a history of alcohol or drug dependence,	**Treatment groups:**<br>• ropinirole (0.75 mg/24 hr up to 24 mg/24 hr maximum)<br>• bromocriptine (1.25 mg/24 hr up to 40 mg/24 hr maximum)	• myocardial infarction<br>• orthostatic hypotension<br>• heart failure

Study	Methods	Participants	Interventions	Outcomes
		severe dementia, or other clinically relevant abnormalities<br>• had previously been treated with ropinirole or had contraindications to bromocriptine or other ergot alkaloids<br>• women of childbearing age were excluded unless they had undergone hysterectomy or surgical sterilization		
LeWitt, 2007 [16]	**Study type:** double-blind, parallel-design RCT<br><br>**Duration:** 24 weeks	**Setting:** multicentre (54 centres in USA and Canada)<br><br>**Number of participants:** 351 randomized (placebo = 120; rotigotine 8 mg/24 hr = 120; rotigotine 12 mg/24 hr = 111)<br><br>**Sex:** % female: placebo = 38.3%; rotigotine 8 mg/24 hr = 39.2%; rotigotine 12 mg/24 hr = 47%<br><br>**Age:** mean (SD) in years: placebo = 66.3 (9.6); rotigotine 8 mg/24 hours = 66.5 (10.0); rotigotine 12 mg/24 hr = 64.5 (10.4)<br><br>**PD duration:** mean (SD) in years: placebo = 7.7 (4.0); rotigotine 8 mg/24 hr = 7.7 (4.3); rotigotine 12 mg/24 hr = 7.8 (4.6)<br><br>**Inclusion criteria:**<br>• at least 30 years of age<br>• diagnosis of idiopathic PD for at least 3 years<br>• clinical features of bradykinesia plus at least one additional cardinal feature	**Control:** placebo<br><br>**Treatment:** rotigotine (in groups of 8 and 12mg/24 hr)	• orthostatic hypotension<br>• peripheral edema

Study	Methods	Participants	Interventions	Outcomes
		(resting tremor, rigidity, impaired postural reflex)<br>• H&Y stage between II and IV in both the "on" and "off " states and were not demented<br><br>**Exclusion criteria:**<br>• subjects needed to demonstrate an average of > 2.5 hr of "off " time (suboptimal control of Parkinsonism) on several 24 hr self-report motor function diaries		
Lieberman, 1997 [17]	**Study type:** double-blind, parallel-design RCT<br><br>**Duration:** 32 weeks	**Setting:** multicentre (26 centres in the US and Canada)<br><br>**Number of participants:** 360 randomized (placebo = 179; pramipexole = 181)<br><br>**Sex:** % female: placebo = 35%; pramipexole = 34%<br><br>**Age:** mean in years: placebo = 63.3; pramipexole = 63.4<br><br>**PD duration:** mean in years: placebo = 9.0; pramipexole = 9.4<br><br>**Inclusion criteria:**<br>• patients of either sex must have been at least 30 years of age with advanced idiopathic PD in stage II to IV as measured by the H&Y scale during an "on" period	**Control:** placebo<br><br>**Treatment:** pramipexole (0.375-4.5 mg/24 hr)	• orthostatic hypotension (symptomatic and asymptomatic)

Study	Methods	Participants	Interventions	Outcomes
		• patients must have continued to experience motor fluctuations specifically characterized as an end-of-dose phenomenon or a "wearing-off" effect while receiving a stable dosage of carbidopa/levodopa for at least 30 days prior to entering the study<br>• patients must have been able to maintain an accurate daily record of times of "on" and "off' periods during waking hours<br><br>**Exclusion criteria:**<br>• atypical Parkinsonian syndromes caused by drugs, encephalitis, progressive supranuclear palsy, or multiple system atrophy<br>• patients with cognitive impairment that could adversely affect the understanding of informed consent, compliance with medication, or maintenance of accurate diaries<br>• patients with second- or third-degree atrioventricular block or sick sinus syndrome, resting heart rate below 50 beats per minute, congestive heart failure, myocardial infarction within 6 months of the study, clinically significant liver or renal disease, or active neoplastic disease<br>• patients who within 180 days of the study had surgery that would negatively impact on their ability to participate or a history of stereotactic brain surgery		

Study	Methods	Participants	Interventions	Outcomes
		• patients with a supine systolic blood pressure less than 100 mmHg or evidence of a 20 mmHg decline in systolic blood pressure plus orthostatic symptoms 1 minute after standing compared with the previous supine systolic blood pressure<br>• females of childbearing potential were required to have a negative pregnancy test at baseline		
Mizuno, 2012 [18]	**Study type:** double-blind, parallel-design RCT<br><br>**Duration:** 12 weeks	**Setting:** multicentre (21 centres in Japan)<br><br>**Number of participants:** 112 randomized (pramipexole IR = 56; pramipexole ER = 56)<br><br>**Sex:** % female: pramipexole IR = 62.5%; pramipexole = 62.5%<br><br>**Age:** mean (SD) in years: pramipexole IR = 66.1 (7.5); pramipexole ER = 66.8 (8.0)<br><br>**PD duration:** mean (SD) in years: pramipexole IR = 3.1 (3.5); pramipexole ER = 2.9 (2.7)<br><br>**Inclusion criteria:**<br>• men and women with PD<br>• patients had a modified H&Y stage of 2 to 4 at on time<br>• receiving levodopa therapy at a stable dose for 4 weeks or longer before the baseline visit<br>• exhibited at least 1 common levodopa-related problem, such as wearing-off, on–off, no-on/delayed-on, off-time dystonia,	**Treatment groups:**<br>• pramipexole IR (4.5 mg/24 hr maximum)<br>• pramipexole ER (4.5 mg/24 hr maximum)	• orthostatic hypotension

Study	Methods	Participants	Interventions	Outcomes
		off-time freezing, or suboptimal clinical condition from suboptimal levodopa dosage because of adverse events or patient and/or physician preference for lower dosage<br><br>**Exclusion criteria:**<br>• atypical Parkinsonian symptoms because of drugs, metabolic disorders, encephalitis, or degenerative diseases<br>• dementia<br>• any psychiatric disorder<br>• history of psychosis, except for drug-induced hallucination<br>• clinically significant electrocardiogram abnormalities<br>• hypotension or symptomatic orthostatic hypotension<br>• patients who had used any DA or medication with central dopaminergic activity, or methylphenidate, within 4 weeks before the baseline visit, or who had previously discontinued pramipexole IR treatment because of adverse events		
Mizuno, 2014 [19]	**Study type:** double-blind RCT<br><br>**Duration:** up to 20 weeks	**Setting:** multicentre (62 centres in Japan)<br><br>**Number of participants:** 420 randomized (rotigotine = 168; ropinirole = 167; placebo = 85)<br><br>**Sex:** % female: rotigotine = 62.8%; ropinirole = 59.0%; placebo = 50.0%	**Control:** placebo<br><br>**Treatment groups:**<br>• rotigotine (2-16 mg/24 hr)<br>• ropinirole (0.75-15 mg/24 hr)	• orthostatic hypotension<br>• valvulopathy<br>• peripheral edema

Study	Methods	Participants	Interventions	Outcomes
		**Age:** mean (SD) in years: rotigotine = 64.8 (8.8); ropinirole = 67.0 (7.9); placebo = 65.3 (7.9)<br><br>**PD duration:** mean (SD) in years rotigotine = 7.0 (4.9); ropinirole = 6.8 (4.2); placebo = 7.0 (4.2)<br><br>**Inclusion criteria:**<br>• 30-79 years of age<br>• diagnosis of PD<br>• H&Y stage of 2-4 and UPDRS Part III sum score of greater than or equal to10 at screening (ON state)<br>• levodopa doses were not changed from the period 28 days before starting treatment<br><br>**Exclusion criteria:**<br>• patients with psychiatric symptoms, orthostatic hypotension, history of epilepsy or convulsion, history of serious cardiac disease, arrhythmia, QT prolongation, abnormal liver function, history of allergy to topical agents<br>• female patients who were pregnant or lactating from the trial<br>• concomitant use of drugs that may affect the symptoms of PD, cause QT prolongation, or interact with ropinirole was prohibited		
Mokhles, 2012 [20]	**Study type:** case–control	**Setting:** multicentre (4 healthcare databases from UK, Netherlands, and Italy)	**Treatment groups:**<br>• levodopa<br>• piribedil	• heart failure

Study	Methods	Participants	Interventions	Outcomes
	**Duration:** 1996–2007	**Number of participants:** 25,459 (controls = 38,641; cases = 518)<br><br>**Sex:** % female: controls = 49.8%; cases = 48.5%<br><br>**Age:** mean (SD) in years: controls = 78.0 (7.2); cases = 79.9 (9.1)<br><br>**Inclusion criteria:**<br>• all individuals from the four databases, who had at least one year of registered medical history prior to study entry<br>• two cohorts of patients who were new users of either DAs (no DA use during the one-year follow-up study before the study entry) or levodopa (no DAs or levodopa in year prior) for PD were included<br><br>**Exclusion criteria:**<br>• participants with heart failure, rheumatic heart disease, valvulopathy, congenital heart disease, dilated or hypertrophic cardiomyopathies, endocarditis, or myocarditis at any time prior to study entry	• pramipexole<br>• ropinirole<br>• rotigotine<br>• bromocriptine<br>• cabergoline	
Moller, 2005 [21]	**Study type:** double-blind RCT<br><br>**Duration:** up to 32 weeks<br><br>**Note:** study's Supplementary	**Setting:** multicentre<br><br>**Number of participants:** 363 randomized (pramipexole = 180;<br>placebo = 183)<br><br>**Sex:** % female: pramipexole = 37.9%; placebo = 32.2%	**Control:** placebo<br><br>**Treatment:** pramipexole (daily range: 0.26-3.15 mg; average 3.7 mg/24 hr)	• asymptomatic postural hypotension

Study	Methods	Participants	Interventions	Outcomes
	Materials are unavailable (limited information on study methods)	**Age:** mean in years: pramipexole = 63.4; placebo = 64.7<br><br>**PD duration:** mean in years pramipexole = 7.6; placebo = 7.9<br><br>**Inclusion criteria:**<br>• had to experience motor fluctuations characterized as end-of-dose phenomena while receiving an individually adjusted stable dosage of levodopa		
Navan, 2003 [22]	**Study type:** double-blind, parallel-design RCT<br><br>**Duration:** 3 months	**Setting:** multicentre (neurological clinics in London and Essex)<br><br>**Number of participants:** 30 randomized (placebo = 10; peroglide = 10; pramipexole = 10)<br><br>**Sex:** % female: placebo = 40%; peroglide = 40%; pramipexole = 30%<br><br>**Age:** mean range in years: placebo = 70 (62–78); peroglide = 71 (54–80); pramipexole = 66 (55–80)<br><br>**PD duration:** mean range in years: placebo = 3 (0.8–7); peroglide = 5 (0.6–8); pramipexole = 4 (0.5–10)<br><br>**Inclusion criteria:**<br>• idiopathic PD	**Control:** placebo<br><br>**Treatment groups:**<br>• pergolide (0.1-1.5 mg/dose, 1-3 times/24 hr)<br>• pramipexole (0.125-1.5 mg/dose, 1-3 times/24 hr)	• symptomatic orthostatic hypotension

Study	Methods	Participants	Interventions	Outcomes
		• a symptomatic tremor of an upper limb that reached at least grade 2/10 in severity on a validated tremor rating scale<br><br>**Exclusion criteria:**<br>• patients previously had not taken any direct acting dopamine agonist class medication, although other anti-Parkinsonian medications were permitted		
Nicholas, 2014 [23]	**Study type:** double-blind, parallel-design RCT<br><br>**Duration:** 12 weeks	**Setting:** multicentre (77 centres in the US, India, Mexico, Peru, and Chile)<br><br>**Number of participants:** 514 randomized (placebo = 108; rotigotine (2 mg/24 hr) = 101; rotigotine (4 mg/24 hr) = 107; rotigotine (6 mg/24 hr) = 104; rotigotine (8 mg/24 hr) = 94)<br><br>Sex: % female: placebo = 31%; rotigotine (1 mg/24 hr) = 24%; rotigotine (4 mg/24 hr) = 26%; rotigotine (6 mg/24 hr) = 30%; rotigotine (8 mg/24 hr) = 40%<br><br>**Age:** mean (SD) in years: placebo = 64.8 (10.20); rotigotine (2 mg/24 hr) = 65.4 (10.5); rotigotine (4 mg/24 hr) = 64.6 (9.0); rotigotine (6 mg/24 hr) = 64.6 (10.4); rotigotine (8 mg/24 hr) = 63.2 (11.6)<br><br>**PD duration:** mean (SD) in years: placebo = 7.23 (3.76); rotigotine (2 mg/24 hr) = 7.51 (3.87); rotigotine (4 mg/24 hr) = 7.27 (3.94); rotigotine (6 mg/24 hr) = 7.79 (3.92); rotigotine (8 mg/24 hr) = 7.49 (4.75)	**Control:** placebo<br><br>**Treatment:** rotigotine (groups divided into doses of 2,4,6, and 8 mg/24 hr)	• orthostatic hypotension<br>• peripheral edema<br>• myocardial infarction<br>• stroke

Study	Methods	Participants	Interventions	Outcomes
		**Inclusion criteria:**<br>• men and women aged ≥ 30 years with idiopathic PD of longer than 3 years duration<br>• presenting with bradykinesia plus at least one of the following: rest tremor, rigidity, or impairment of postural reflexes<br>• patients were within H&Y stage II–IV in both the 'on' and 'off' states<br>• Mini-Mental State Examination score of at least 25<br>• judged by the treating physician to be inadequately controlled on levodopa in combination with benserazide or carbidopa, with an average 'off' time of ≥ 2.5 hr/24 hr<br><br>**Exclusion criteria:**<br>• previous experience of a dopaminergic complication<br>• previous history of freezing<br>• atypical Parkinsonian syndromes caused by drugs, metabolic disorders, encephalitis, or degenerative diseases<br>• dementia<br>• serious concurrent illness<br>• symptomatic orthostatic hypotension at the screening visit<br>• electroconvulsive therapy in the previous 90 days<br>• history of stereotaxic brain surgery for PD		

Study	Methods	Participants	Interventions	Outcomes
Pahwa, 2007 [24]	**Study type:** double-blind, parallel-design RCT<br><br>**Duration:** 24 weeks	**Setting:** 67 centres in Belgium, the Czech Republic, France, Hungary, Italy, Poland, Spain, and the USA<br><br>**Number of participants:** 393 randomized (placebo = 191; ropinirole = 202)<br><br>**Sex:** % female: placebo = 32%; ropinirole = 42%<br><br>**Age:** mean (SD) in years: placebo = 66 (9.7); ropinirole = 66.3 (9.2)<br><br>**PD duration:** mean (SD) in years: placebo = 8.6 (5.2); ropinirole = 8.6 (4.8)<br><br>**Inclusion criteria:**<br>• men and women at least 30 years of age<br>• diagnosis of idiopathic PD and a modified H&Y stage of II to IV with suboptimal control with levodopa therapy<br><br>**Exclusion criteria:**<br>• patients with incapacitating peak dose or biphasic dyskinesia<br>• any dopamine agonist use within 4 weeks of screening<br>• significant or uncontrolled psychiatric, neurologic, or other medical disorders<br>• clinically significant laboratory abnormalities at screening<br>• a recent history of severe dizziness or fainting due to postural hypotension	**Control:** placebo<br>**Treatment:** ropinirole (18.8 mg/24 hr)	• orthostatic hypotension<br>• syncope

Study	Methods	Participants	Interventions	Outcomes
		• clinical dementia<br>• a recent history or current evidence of drug abuse or alcoholism<br>• withdrawal, introduction, or dose change of hormone replacement therapy, or any drug known to substantially inhibit or induce cytochrome P450 1A2		
Peralta, 2006 [25]	**Study type:** cohort<br><br>**Duration:** ~ 2003–2004	**Setting:** not specified<br><br>**Number of participants:** 124 (controls = 49; peroglide = 29; cabergoline = 13; non-ergot group (ropinirole and pramipexole = 33)<br><br>**Sex:** % female: controls = 29%; peroglide = 14%; cabergoline = 31%; non-ergot group (ropinirole and pramipexole): 48%<br><br>**Age:** mean (SD) years: controls = 64 (5.5); peroglide = 65 (7.7); cabergoline = 65 (11.5); non-ergot group = 62 (7.7)<br><br>**PD duration:** mean (SD) in years: peroglide = 10.5 (4.1); cabergoline = 8.3 (4.0); non-ergot group = 6.4 (3.8)<br><br>**Inclusion criteria:**<br>• PD patients treated with dopamine agonists for a minimum of 12 months<br><br>**Exclusion criteria:** not specified	**Treatment groups:**<br>• pergolide (cumulative dose 3.2 mg)<br>• cabergoline (cumulative dose 4.0 mg)<br>• ropinirole (cumulative dose 14.25 mg)<br>• pramipexole (cumulative dose 2.09 mg)	• heart failure<br>• syncope<br>• valvulopathy<br><br>**Note:** effect estimates not reported
Poewe, 2007 [26]	**Study type:** double-blind RCT	**Setting:** multicentre (77 centres in Europe, South Africa, Australia, and New Zealand)	**Control:** placebo<br><br>**Treatment groups:**	• orthostatic hypotension

Study	Methods	Participants	Interventions	Outcomes
	**Duration:** 23 weeks	**Number of participants:** 506 participants randomized (placebo = 101; pramipexole = 101; rotigotine = 204)<br><br>**Sex:** % female: placebo = 29%; pramipexole = 44%; rotigotine = 34%<br><br>**Age:** mean (SD) in years: placebo = 65.0 (10.0); pramipexole = 63.2 (9.7); rotigotine = 64.3 (9.0)<br><br>**PD duration:** mean (SD) in years: placebo = 8.5 (5.0); pramipexole = 8.4 (4.7); rotigotine = 8.9 (4.4)<br><br>**Inclusion criteria:**<br>• 30 years or older (for sites in South Africa: 30-80 years)<br>• diagnosed with idiopathic PD for more than 3 years and on stable treatment<br>• had motor fluctuations of the wearing-off type with an average of at least 2-5 hr/24 hr spent in the "off" state, as assessed by home diaries completed over 6 days before enrollment (recordings covered the 24 hr/24 hr and patients had to mark 30 min intervals as being either "on" without troublesome dyskinesias, on with troublesome dyskinesias, off, or asleep), and were graded no better than H&Y stage II when on and no worse than stage IV when off<br><br>**Exclusion criteria:**	• pramipexole (0.375-4.5 mg/24 hr)<br>• rotigotine (4-16 mg/24 hr)	• symptomatic orthostatic hypotension

Study	Methods	Participants	Interventions	Outcomes
		• more than 2 of the 6 screening diaries were invalid (a diary was considered invalid if more than 2 hr of data during a 24 hr recording day were missing or recorded as double entries)<br>• had received concomitant treatment with any dopamine agonist during the 4 weeks before starting the 6 screening diary recordings<br>• suspicion of atypical Parkinsonism<br>• previous surgery for PD<br>• Mini-Mental State Examination score < 25<br>• concurrent hallucination or psychosis<br>• history of orthostatic hypotension 6 months before baseline<br>• history of myocardial infarction over past 12 months<br>• QTc interval > 450 ms (men) or > 470 ms (women)<br>• history of skin hypersensitivity to adhesives or other transdermals<br>• intake of investigational drug within 4 weeks before pretreatment visit<br>• concomitant treatment with dopamine agonists, monoamine oxidase A inhibitors, dopamine-releasing drugs, tolcapone, neuroleptics, cimetidine, ranitidine, diltiazem, triamterene, verapamil, quinidine, or quinine		
Rascol, 1996 [27]	**Study type:** double-blind RCT<br><br>**Duration:** 12 weeks	**Setting:** multicentre (France and England)<br><br>**Number of participants:** 46 participants randomized (placebo =	**Control:** placebo<br><br>**Treatment:** ropinirole (4 mg/dose maximum)	• postural hypotension

Study	Methods	Participants	Interventions	Outcomes
		23; ropinirole = 23)<br><br>**Sex:** % female: placebo = 39%; ropinirole = 39%<br><br>**Age:** mean (SD) in years: placebo = 63 (9); ropinirole = 62 (7)<br><br>**PD duration:** mean (SD) in years: placebo = 8 (3); ropinirole = 8 (2)<br><br>**Inclusion criteria:**<br>• aged 30 to 80 years<br>• clinical diagnosis of PD<br>• with moderate disability not controlled by levodopa<br><br>**Exclusion criteria:**<br>• with severe disability<br>• require subcutaneous injections of apomorphine<br>• with unpredictable oscillations, disabling dyskinesia, symptomatic orthostatic hypotension, psychosis, dementia, and other neurologic disorders (aside from PD)		
Rascol, 1998 [28]	**Study type:** double-blind RCT<br><br>**Duration:** 6 months	**Setting:** multicentre (30 centres in Europe, Israel, and Canada)<br><br>**Number of participants:** 268 participants randomized (levodopa = 89; ropinirole = 179)	**Treatment groups:**<br>• levodopa (1200 mg/24 hr maximum)<br>• ropinirole (24 mg/24 hr maximum)	• syncope

Study	Methods	Participants	Interventions	Outcomes
		**Sex:** % female: levodopa = 42.7%; ropinirole = 36.9%<br><br>**Age:** mean age: SD in years: levodopa = 63 (9); ropinirole = 63 (9)<br><br>**Inclusion criteria:**<br>• at least 30 years old<br>• clinical diagnosis of idiopathic PD<br>• early stage of the disease (H&Y stages I-III)<br>• required dopaminergic therapy<br><br>**Exclusion criteria:**<br>• severe systemic or psychiatric disease<br>• history of drug or alcohol dependence<br>• severe dementia<br>• other clinically relevant abnormalities<br>• patients with evidence of postural hypotension		
Rascol, 2000 [29]	**Study type:** double-blind RCT<br><br>**Duration:** 5 years	**Setting:** multicentre (30 centres in Israel, Europe, and Canada)<br><br>**Number of participants:** 268 randomized (ropinirole = 179; levodopa = 89)<br><br>**Sex:** % female: ropinirole = 36.9%; levodopa = 41.6%<br>Age: mean (SD) in years: ropinirole = 63 (9); levodopa = 63 (9)<br><br>**PD duration:** mean (SD) in months ropinirole = 30 (34); levodopa = 29 (27)	**Treatment groups:**<br>• ropinirole (0.75 mg/24 hr up to 24 mg/24 hr maximum)<br>• levodopa (50 mg/24 hr up to 1200 mg/24 hr maximum)	• postural hypotension

Study	Methods	Participants	Interventions	Outcomes
		**Inclusion criteria:**<br>• 30 years of age or older<br>• had a clinical diagnosis of PD with an H&Y rating of stage I-III and required dopaminergic therapy<br>• short-term treatment with levodopa or dopamine agonists was limited to a maximum of 6 weeks and had to be discontinued at least 2 weeks before entry into the study<br><br>**Exclusion criteria:**<br>• if they had severe dizziness or fainting, severe systemic disease, major psychosis, severe dementia, alcoholism or drug dependence, or a contraindication to levodopa<br>• treatment with a monoamine oxidase inhibitor within two weeks before entry (with the exception of selegiline) or previous treatment with ropinirole were reasons for exclusion		
Rascol, 2006 [30]	**Study type:** double-blind RCT<br><br>**Duration:** 7 months	**Setting:** multicentre<br>Number of participants: 401 participants randomized (placebo = 204; piribedil = 197)<br><br>**Sex:** % female: placebo = 37.3%; piribedil = 41.1%<br><br>**Age:** mean age (SD) in years: placebo = 62.3 (10.3); piribedil = 62.4 (9.5)	**Treatment groups:**<br>• piribedil (50-300 mg/24 hr)<br>• placebo	• postural hypotension<br>• hypertension<br>• peripheral edema

Study	Methods	Participants	Interventions	Outcomes
		**PD duration:** mean (SD) in years: placebo = 2.0 (2.0); piribedil = 2.0 (1.8)<br><br>**Inclusion criteria:**<br>• aged 30 to 77 years<br>• diagnosis of idiopathic PD (according to Queen Square Brain Bank for Neurological Disorders criteria) at stage I-III on the H&Y scale<br><br>**Exclusion criteria:**<br>• treatment with nonselective monoamine oxidase inhibitors, amineptine, imipramine, and derivates		
Renoux, 2012 [31]	**Study type:** case–control<br><br>**Duration:** 1997-2009	**Setting:** UK General Practice Research Database<br><br>**Number of participants:** 26,814 (controls=7454; cases=783)<br><br>**Sex:** % female: controls = 44.4%; cases = 44.4%<br><br>**Age:** mean (SD) in years: controls = 78.8 (6.8); cases = 79.2 (7.3)<br><br>**PD duration:** mean (SD) in years: controls = 3.2 (2.6); cases = 3.2 (2.7)<br><br>**Inclusion criteria:**<br>• users of anti-Parkinsonian drugs registered with an up-to-standard General Practice Research Database practice	**Treatment groups:**<br>• pramipexole<br>• ropinirole<br>• cabergoline<br>• pergolide<br>• other DAs<br><br>**Control:** non-use of DAs	• heart failure

Study	Methods	Participants	Interventions	Outcomes
		• were 40 to 89 years of age between 1 January 1997 and 30 June 2009<br><br>**Exclusion criteria:**<br>• prior diagnosis of heart failure or had prescriptions suggestive of treated heart failure, which was defined as a digitalis in association with an angiotensin-converting enzyme, inhibitors/angiotensin receptor blocker, or with diuretics, or both, any time before cohort entry<br>• history of heart failure after cohort entry without a prior heart failure diagnosis as the date of heart failure diagnosis could not be determined		
Sampaio, 2011 [32]	**Study type:** double-blind RCT (Vermeer study only extracted)<br><br>**Duration:** 28-31 weeks	**Setting:** multicentre (78 centres)<br><br>**Number of participants:** 334 randomized (pramipexole = 116; pardoprunox = 108; placebo = 110)<br><br>**Sex:** % female: pramipexole = 46.1%; pardoprunox = 47.1%; placebo = 37.3%<br><br>**Age:** mean (SD) in years: pramipexole = 60.8 (11.7); pardoprunox = 62.9 (10.6); placebo = 62.8 (8.9)<br><br>**PD duration:** mean (SD) in months pramipexole = 11.1 (13.7); pardoprunox = 11.8 (14.9); placebo = 10.1 (11.3)<br><br>**Inclusion criteria:**	**Control:** placebo<br><br>**Treatment groups:**<br>• pramipexole (flexible dose: 1.5–4.5 mg/24 hr)<br>• pardoprunox (flexible dose: 12–42 mg/24 hr)	• peripheral edema

Study	Methods	Participants	Interventions	Outcomes
		• outpatients 30 years of age or older with a diagnosis of idiopathic PD and having a modified H&Y stage less than or equal to III<br>• patients had a total UPDRS–motor score greater than or equal to 10 at baseline<br><br>**Exclusion criteria:**<br>• presence of dyskinesias, motor fluctuations, or loss of postural reflexes, an unclear diagnosis, or suspicion of secondary Parkinsonism, Parkinson-plus syndromes, or other hereditary degenerative disease, or a current primary psychiatric diagnosis<br>• forbidden concurrent medications included opioids, anticonvulsants, and sympathomimetics, psychostimulants, and antipsychotics<br>• prophylactic use of antiemetics was forbidden		
Schapira, 2011 [33]	**Study type:** double-blind RCT<br><br>**Duration:** up to 33 weeks	**Setting:** multicentre (76 centres in Austria, Czech Republic, Hungary, India, Italy, Philippines, Poland, Russia, Slovakia, South Korea, Spain, Sweden, Ukraine, and the UK)<br><br>**Number of participants:** 518 participants randomized (placebo = 178; pramipexole IR = 164; pramipexole ER = 175)<br><br>**Sex:** % female: placebo = 47.2%; pramipexole ER = 43.9%; pramipexole IR = 44%	**Control:** placebo<br><br>**Treatment groups:**<br>• pramipexole ER (0.375–4.5 mg/ 24 hr)<br>• pramipexole IR (0.125–1.5 mg/ 24hr)	• orthostatic hypotension

Study	Methods	Participants	Interventions	Outcomes
		**Age:** mean (SD) in years: placebo = 60.9 (9.7); pramipexole ER = 61.6 (9.7); pramipexole IR = 62.0 (10.3);<br><br>**PD duration:** mean (SD) in years: placebo = 5.9 (3.8); pramipexole ER = 6.1 (4.0); pramipexole IR = 6.6 (4.4)<br><br>**Inclusion criteria:**<br>• 30 years old<br>• idiopathic PD at H&Y stage II-IV during on time<br>• were diagnosed 2 years before entry<br>• were being treated with levodopa at an optimized dosage (investigator's judgment) unchanged during at least the 4 weeks before baseline<br>• patients were also required to experience motor fluctuations (2 cumulative hrs of daily off time during waking hrs, documented as described below, on 2 consecutive days)<br><br>**Exclusion criteria:**<br>• patients were excluded for a Mini-Mental State Examination score < 24<br>• atypical Parkinsonian syndromes<br>• history of deep brain stimulation<br>• psychiatric or non-PD medical disorders capable of impeding trial participation<br>• clinically significant hypotension or electrocardiographic abnormalities		

Study	Methods	Participants	Interventions	Outcomes
		• creatinine clearance < 50 mL/min		
Schapira, 2013 [34]	**Study type:** double-blind RCT<br><br>**Duration:** 15 months	**Setting:** 98 centres in ten countries (Austria, Finland, France, Germany, Italy, Japan, Spain, Sweden, the UK, and the USA)<br><br>**Number of participants:** 535 participants randomized (delayed pramipexole = 274; early pramipexole = 261)<br><br>**Sex:** % female: delayed pramipexole: 39%; early pramipexole: 32%<br><br>**Age:** mean (SD) in years: delayed pramipexole: 62.9 (9.9); early pramipexole: 62.1 (10.1)<br><br>**PD duration:** mean (SD) in months: delayed pramipexole: 4.5 (5.9); early pramipexole: 4.4 (6.3)<br><br>**Inclusion criteria:**<br>• patients were 30–79 years<br>• had idiopathic PD characterised by bradykinesia plus at least two PD signs (resting tremor, rigidity, or asymmetry)<br>• were at modified H&Y stage I or II<br>• were diagnosed within the preceding 2 years<br>• were unlikely to need symptomatic treatment for at least the next 6 months, preferably 9 months<br><br>**Exclusion criteria:**	**Treatment groups:**<br>• delayed pramipexole (1.5 mg/24 hr)<br>• early pramipexole (1.5 mg/24 hr)	• peripheral edema

Study	Methods	Participants	Interventions	Outcomes
		• were currently using PD drugs<br>• had used antipsychotic drugs within the preceding 6 months<br>• had any clinically significant abnormalities unrelated to PD in physical findings or laboratory values<br>• patients with medical or psychiatric disorders capable of interfering with study participation or the interpretation of study data<br>• history of psychosis, dementia, or major or seasonal depression		
Seiple, 2016 [35]	**Study type:** RCT<br><br>**Duration:** 2 years	**Setting:** multicentre (21 neurology and 19 ophthalmology sites in the USA)<br><br>**Number of participants:** 246 participants randomized (pramipexole = 121; ropinirole = 125)<br><br>**Sex:** % female: pramipexole = 34.7%; ropinirole = 37.6%<br><br>**Age:** mean (SD) in years: pramipexole = 57.5 (9.3); ropinirole = 59.1 (8.7)<br><br>**PD duration:** mean (SD) in years: pramipexole = 0.97 (1.15); ropinirole = 1.29 (1.70)<br><br>**Inclusion criteria:**	**Treatment groups:**<br>• pramipexole (0.375–4.5 mg/24 hr)<br>• ropinirole (0.75–24.0 mg/24 hr)	• orthostatic hypotension<br>• peripheral edema

Study	Methods	Participants	Interventions	Outcomes
		• ≥ 30 years old with idiopathic PD of ≤7 years duration, with modified H&Y stages I–III<br>• with ≤ 6 months cumulative lifetime dopamine agonist exposure<br><br>**Exclusion criteria:**<br>• nonidiopathic PD<br>• prior stereotactic brain surgery<br>• existing eye abnormalities<br>• select medical conditions		
Stocchi, 2011 [36]	**Study type:** double-blind RCT<br><br>**Duration:** 24 weeks	**Setting:** multicentre<br><br>**Number of participants:** 350 participants randomized (ropinirole PR=177; ropinirole IR=173)<br><br>**Sex:** % female: ropinirole PR = 40%; ropinirole IR = 46%<br><br>**Age:** mean (SD) in years: ropinirole PR = 64.9 (9.20); ropinirole IR = 65.6 (9.01)<br><br>**PD duration:** mean (SD) in years: ropinirole PR = 7.9 (4.79); pramipexole IR = 7.5 (5.04)<br><br>**Inclusion criteria:**<br>• at least 30 years old<br>• with a diagnosis of idiopathic PD (H&Y stage II–IV)<br>• demonstrating a lack of control with levodopa therapy	**Treatment groups:**<br>• ropinirole PR (2-24 mg/24 hr)<br>• ropinirole IR (0.75-24 mg/24 hr)	• orthostatic hypotension<br>• hypotension

Study	Methods	Participants	Interventions	Outcomes
		• a stable dose of levodopa for at least 4 weeks before baseline and between 3 and 12 hrs of daily awake time spent ‘‘off’’ as measured using patient diaries during the 2-week baseline period were required<br><br>**Exclusion criteria:**<br>• had incapacitating dyskinesia, significant or uncontrolled psychiatric, neurologic, or other medical disorders (including postural hypotension), or clinically significant laboratory abnormalities at screening<br>• had used a DA within 4 weeks of the screening visit or had withdrawal, introduction, or dose change of hormone replacement therapy or any drug known to substantially inhibit or induce cytochrome P450 1A2		
Titlic, 2008 [37]	**Study type:** cohort<br><br>**Duration:** August 2003–May 2005	**Setting:** 2 hospitals<br><br>**Number of participants:** 102 (levodopa=52; ropinirole=50)<br><br>**Sex:** % female: levodopa = 63%; ropinirole = 62%<br><br>**Age:** mean (SD) in years: levodopa = 63.2 (4.1); ropinirole = 61.4 (4.3)<br><br>**PD duration:**<br>0.5-2 years (range for duration of PD symptoms)	**Treatment groups:**<br>• levodopa (dose not reported)<br>• ropinirole (4-6 mg/24 hr)	• orthostatic hypotension

Study	Methods	Participants	Interventions	Outcomes
		**Inclusion criteria:**<br>• PD patients administered ropinirole therapy for the first time<br><br>**Exclusion criteria:**<br>• with depression and dementia		
Viallet, 2013 [38]	**Study type:** double-blind RCT<br><br>**Duration:** 15 weeks	**Setting:** multicentre<br><br>**Number of participants:** 109 participants randomized (pramipexole = 56; rasagiline = 53)<br><br>**Sex:** % female: pramipexole = 44.6%; rasagiline = 30.2%<br><br>**Age:** mean (SD) in years: pramipexole = 62.1 (6.2); rasagiline = 63.2 (7.3)<br><br>**PD duration:** mean (SD) in years: pramipexole = 4.3 (7.3); rasagiline = 2.5 (3.8)<br><br>**Inclusion criteria:**<br>• never received anti-PD treatment or had received levodopa for less than 12 weeks at a dose less than 200 mg<br>• discontinued all anti-PD treatment other than the study drugs as part of the study protocol<br><br>**Exclusion criteria:**<br>• pregnant or breastfeeding women or women of a childbearing age without	**Treatment groups:**<br>• pramipexole (1.5 mg/24 hr)<br>• rasagiline (1 mg/24 hr)	• orthostatic hypotension<br>• syncope

Study	Methods	Participants	Interventions	Outcomes
		sterilization or a reliable birth control method<br>• liver disease<br>• concomitant disease considered to be significant by the investigator<br>• treated with cerebral stimulation<br>• skin lesions not assessed by a dermatologist<br>• treated with fluoxetine during the 5 weeks preceding inclusion; those treated with fluvoxamine, pethidine, selegiline, or any other monoamine oxidase-B inhibitors during the 2 weeks preceding inclusion; and patients likely to receive dextromethorphan or a sympathomimetic drug during the trial		
Wang, 2014 [39]	**Study type:** double-blind RCT<br><br>**Duration:** 18 weeks	**Setting:** multicentre (20 centres in China)<br><br>**Number of participants:** 475 participants randomized (pramipexole ER = 236; pramipexole IR = 239)<br><br>**Sex:** % female: pramipexole ER = 34%; pramipexole IR = 40%<br><br>**Age:** mean (SD) in years: pramipexole ER = 62.2 (9.10); pramipexole IR = 61.8 (9.03)<br><br>**PD duration:** mean (SD) in years: pramipexole ER = 5.11 (3.33); pramipexole IR = 4.82 (3.09)<br><br>**Inclusion criteria:**	**Treatment groups:**<br>• pramipexole ER (0.375-4.5 mg/24 hr)<br>• pramipexole IR (0.375.4.5 mg/24 hr)	• orthostatic hypotension<br>• hypotension

Study	Methods	Participants	Interventions	Outcomes
		• Chinese patients diagnosed with idiopathic PD for at least 2 years<br>• age ≥ 30 years old at diagnosis, modified H&Y score of 2 to 4 during on time<br>• patients taking standard or controlled release levodopa or levodopa/entacapone, dose has to be optimized and stable for at least 4 weeks prior to the baseline visit<br>• motor fluctuations while taking levodopa, 'off' time at waking should be no more than 6 hours daily during 2 consecutive days before the baseline visit<br><br>**Exclusion criteria:**<br>• patients without UPDRS II and III score at baseline or during treatment		
Watts, 2007 [40]	**Study type:** double-blind RCT<br><br>**Duration:** 7 months	**Setting:** multicentre (50 centres in Canada and the USA)<br><br>**Number of participants:** 277 randomized (rotigotine = 181; placebo = 96)<br><br>**Sex:** % female: rotigotine = 32%; placebo = 40%<br><br>**Age:** mean (SD) in years: rotigotine = 62.0 (10.3); placebo = 64.5 (10.7)<br><br>**PD duration:** mean (SD) in years rotigotine = 1.3 (1.3); placebo = 1.4 (1.3)<br><br>**Inclusion criteria:**<br>• 30 years of age or older	**Control:** placebo<br><br>**Treatment:** rotigotine (2-6 mg/24 hr)	• peripheral edema<br>• orthostatic hypotension

Study	Methods	Participants	Interventions	Outcomes
		• idiopathic PD of less than or equal to 5 years in duration<br>• UPDRS Motor Function Examination (part III) score of at least 10 at baseline<br>• H&Y stage score less than or equal to III<br>• two or more of the following cardinal signs of PD (bradykinesia, resting tremor, rigidity, or postural instability)<br>• Mini-Mental State Examination score of 25 or more<br>• no other known or suspected cause of Parkinsonism<br>• had received an anticholinergic agent, monoamine oxidase-B inhibitor, or amantadine (must have been on a stable dose for at least 28 days prior to study baseline and must be maintained on that dose for the duration of the trial)<br><br>**Exclusion criteria:**<br>• prior or concurrent therapy with a dopamine agonist or carbidopa/levodopa within 28 days of the baseline visit or carbidopa/levodopa therapy lasting for more than 6 months since diagnosis<br>• atypical Parkinsonism<br>• surgical intervention for PD<br>• clinically relevant hepatic, renal, or cardiac dysfunction; a diagnosis of epilepsy; a history of seizures as an adult; stroke or a transient ischemic attack within the last year; significant skin hypersensitivity to adhesive or other		

Study	Methods	Participants	Interventions	Outcomes
		transdermals or recent unresolved contact dermatitis; known intolerance/hypersensitivity to the antiemetic ondansetron; pregnancy or nursing; and inadequate birth control methods<br>• patients receiving central nervous system active therapy were excluded, unless their pharmacotherapy dose(s) had been stable for at least 28 days prior to baseline and was likely to remain stable for the duration of the trial		
Wermuth, 1998 [41]	**Study type:** double-blind, parallel-design RCT<br><br>**Duration:** 12 weeks	**Setting:** multicentre<br><br>**Number of participants:** 69 randomized (placebo = 33; pramipexole = 36)<br><br>**Sex:** % female: pramipexole = 39.4%; pramipexole = 44.5%<br><br>**Age:** mean (SD) in years: pramipexole = 62.1 (9.9); pramipexole = 63.2 (7.9)<br><br>**PD duration:** mean (SD) in years: pramipexole = 9.9 (4.1); pramipexole = 10.1 (5.0)<br><br>**Inclusion criteria:**<br>• females without childbearing potential or males aged 30-75 years with advanced disease as defined by H&Y stage II-IV, in whom the optimized dose of levodopa and a dopa decarboxylase inhibitor was accompanied by dyskinesia, "on-off"	**Control:** placebo<br><br>**Treatment:** pramipexole (0.2-0.5 mg/24 hr)	• postural hypotension<br>• hypertension

Study	Methods	Participants	Interventions	Outcomes
		fluctuation, and dystonia, akinesia, or end-of-dose deterioration<br><br>**Exclusion criteria:**<br>• patients with other serious diseases that might interfere with study evaluations<br>• in particular, patients were excluded if they had symptomatic Parkinsonian syndromes, PD resistant to dopaminergic agonists, dementia, epilepsy, prior brain operations within the past 10 years, or prior intracranial vessel operations within the past 6 months<br>• patients with significant renal, hepatic, or metabolic disorders were also excluded, as were patients with severe cardiovascular disease, including conduction abnormalities, myocardial infarction within the prior 6 months, or uncontrolled hypertension or hypotension<br>• the emergence of any of these exclusionary criteria during the study, as well as serious adverse events, was cause for withdrawing a patient from the study		
Yamamoto, 2006 [42]	**Study type:** case–control<br><br>**Duration:** September 2004–September 2005	**Setting:** single centre in Japan<br><br>**Number of participants:** 210 consecutive patients (control = 85; peroglide = 66; cabergoline = 16; pramipexole = 16; past treated ergot-derived DA = 27)	**Control:** not treated with any DAs<br><br>**Treatment groups:**<br>• pergolide (1.4 mg/24 hr)<br>• cabergoline (3.8 mg/24 hr)	• valvulopathy

Study	Methods	Participants	Interventions	Outcomes
		**Sex:** % female: control = 59%; peroglide = 52%; cabergoline = 69%; pramipexole = 56%; Past treated ergot-derived DA = 70%<br><br>**Age:** mean (SD) in years: control = 70.4 (8.9); peroglide = 66.3 (9.8); cabergoline = 64.5 (8.4); pramipexole = 70 (6.7); past treated ergot-derived DA = 68.6 (7.2)<br><br>**PD duration:** mean (SD) in years: control = 5 (4.9); peroglide = 9.7 (5.5); cabergoline = 7.8 (4.4); pramipexole = 5.2 (4); past treated ergot-derived DA = 10.3 (4.7)<br><br>**Inclusion criteria:**<br>• patients with PD<br>• recruited consecutively without any clinical reason for transthoracic echocardiography<br><br>**Exclusion criteria:**<br>not specified	• pramipexole (1.7 mg/24 hr)<br>• past treated ergot-derived DA	
Yamashiro, 2008 [43]	**Study type:** cohort<br><br>**Duration:** August 2004 to May 2006	**Setting:** single centre in Japan<br><br>**Number of participants:** 527 (controls = 79; peroglide = 194; cabergoline = 153; bromocriptine = 28; pramipexole = 51; talipexole = 22<br><br>**Sex:** % female: controls = 39 (49.4); peroglide = 101 (52.1); cabergoline = 67 (43.8); bromocriptine = 10 (35.7); pramipexole = 27 (52.9); talipexole = 9 (40.9)	**Control:** untreated<br><br>**Treatment groups:**<br>• pergolide (1.2 mg/24 hr)<br>• cabergoline (2.5 mg/24 hr)<br>• bromocriptine (11.2 mg/24 hr)<br>• pramipexole (1.3 mg/24 hr)	• valvulopathy (tricuspid, mitral, aortic regurgitation)

Study	Methods	Participants	Interventions	Outcomes
		**Age:** mean (SD) in years: controls = 65.8 (6.6); peroglide = 64.0 (8.1); cabergoline = 65.3 (8.9); bromocriptine = 65.1 (10.2); pramipexole = 64.1 (8.9); talipexole = 71.1 (7.3)<br><br>**Inclusion criteria:**<br>• consecutive PD patients who visited the clinic<br><br>**Exclusion criteria:**<br>• control group patients had never been treated with a DA<br>• patients who were switched from one ergot dopamine agonist to another were excluded from the study	• talipexole (1.4 mg/24 hr)	
Zesiewicz, 2017 [44]	**Study type:** parallel-design RCT<br><br>**Duration:** 17 weeks	**Setting:** multicentre (41 centres across Argentina, Chile, Estonia, the Russian Federation, Slovakia, the Republic of Korea, and the USA)<br><br>**Number of participants:** 352 randomized (placebo = 74; ropinirole PR 4 mg = 25; ropinirole PR 8mg = 76; ropinirole PR 12 mg = 75; ropinirole PR 16 mg = 75; ropinirole PR 24 mg = 25)<br><br>**Sex:** % female: placebo = 55%; ropinirole PR 4 mg = 48%; ropinirole PR 8 mg = 43%; ropinirole PR 12 mg = 44%; ropinirole PR 16 mg = 49%; ropinirole PR 24 mg = 40%	**Control:** placebo<br><br>**Treatment:** ropinirole PR (in groups of 4, 8, 12, 16, and 24 mg/24 hr)	• hypertension

Study	Methods	Participants	Interventions	Outcomes
		**Age:** mean (SD) in years: placebo = 63.8 (10.02); ropinirole PR 4 mg = 66.5 (7.45); ropinirole PR 8 mg = 65.6 (9.19); ropinirole PR 12 mg = 65.2 (9.62); ropinirole PR 16 mg = 63.8 (9.15); ropinirole PR 24 mg = 66.9 (7.94)<br><br>**Inclusion criteria:**<br>• male and nonpregnant/non-breastfeeding women ≥30 years of age<br>• a diagnosis of idiopathic PD (modified H&Y stages II-IV) demonstrating a lack of control with levodopa (such as end-of-dose akinesia, simple 'on/off' fluctuations)<br>• receiving a stable dose of levodopa for ≥ 4 weeks prior to screening and ≥ 3 hr awake 'off' time per diary day at baseline<br><br>**Exclusion criteria:**<br>• an incapacitating peak dose or diphasic dyskinesia on their stable levodopa dose<br>• DA treatment within 4 weeks of study randomization or physical or mental conditions that would preclude accurate assessment of safety or efficacy<br>• clinically significant ECG or laboratory test abnormalities at screening<br>• a definite or suspected personal or family history of adverse reactions or hypersensitivity to ropinirole; any withdrawal, introduction, or dose change of hormone replacement therapy and/or drugs known to inhibit or induce CYP450 CYP1A2 activity		

Study	Methods	Participants	Interventions	Outcomes
		• use of investigational drugs from 30 days or five half-lives prior to enrollment or a personal history of melanoma		
Zhang, 2013 [45]	**Study type:** double-blind, parallel-design RCT<br><br>**Duration:** 24 weeks	**Setting:** multicentre (20 centres in China)<br><br>**Number of participants:** 347 randomized (placebo = 171; ropinirole PR = 176)<br><br>**Sex:** % female: placebo = 38.2%; ropinirole PR = 34.3%<br><br>**Age:** mean (SD) in years: placebo = 63.6 (10.5); ropinirole PR = 64.1 (9.0)<br><br>**PD duration:** mean (SD) in months: placebo = 95.7 (48.3); ropinirole PR = 92.1 (59.9)<br><br>**Inclusion criteria:**<br>• Chinese men and women aged >30 years with a diagnosis of idiopathic PD and a modified H&Y stage II-IV demonstrating lack of symptomatic control with a stable dose of levodopa (such as end of dose akinesia, simple "on/off" fluctuations)<br>• subjects had to be receiving a stable dose of levodopa for at least 4 weeks prior to screening and had a minimum of 3 hrs awake time "off" for each diary day recorded during the placebo run-in period<br><br>**Exclusion criteria:**	**Control:** placebo<br><br>**Treatment:** ropinirole PR (2-24 mg/24 hr)	• orthostatic hypotension<br>• hypertension<br>• peripheral edema<br>• arrhythmia<br><br>**Note:** Arrythmia data were extracted from clinicaltrials.gov

Study	Methods	Participants	Interventions	Outcomes
		• subjects with an incapacitating peak dose or biphasic dyskinesia on their stable dose of levodopa<br>• a history of alcoholism, severe dizziness, or fainting; consumption of any dopamine agonist within 4 weeks of the screening visit<br>• use of any drug known to substantially inhibit cytochrome P4501A2 or any investigational drug within 30 days or five half-lives (whichever was longer) of study commencement		
Ziegler, 2003 [46]	**Study type:** double-blind, parallel-design RCT<br><br>**Duration:** 6 months	**Setting:** multicentre (31 centres in France and Portugal)<br><br>**Number of participants:** 115 randomized (placebo = 54; piribedil = 61)<br><br>**Sex:** % female: placebo = 48%; piribedil = 34%<br><br>**Age:** mean (SD) in years: placebo = 64.8 (7.6); piribedil = 63.4 (7.3)<br><br>**PD duration:** mean (SD) in months: placebo = 48 (28); piribedil = 55 (33)<br><br>**Inclusion criteria:**<br>• male and female patients<br>• 35 to 75 years of age<br>• clinical diagnosis of idiopathic PD of less than 10 years duration (stages I–III on the H&Y scale)	**Control:** placebo<br><br>**Treatment:** piribedil (50-150 mg/24 hr)	• orthostatic hypotension

Study	Methods	Participants	Interventions	Outcomes
		• previous treatment with DAs, anticholinergics, and amantadine had to be discontinued for at least 1 month before screening<br>• eligible patients had to be receiving levodopa treatment for more than 6 months and less than 8 years and suffering from stable residual Parkinsonism on a stable dosage of levodopa (150 mg and 800 mg) combined with carbidopa or benserazide for at least 1 month before inclusion<br>• patients treated with selegiline could participate as long as they were on stable dosage for at least 1 month before enrollment<br><br>**Exclusion criteria:**<br>• patients with motor fluctuations		

Abbreviations: BID: two times a day; TID: three times a day.

Supplementary Material S5. Additional tables.

Table S5.1. Findings from observational studies that reported counts of events.

Reference	Outcome	Treatment: Number of patients with event/number of patients in group
Peralta, 2006 [25]	Heart failure	Controls: 0/49<br>Pramipexole: 0/25
	Syncope	Controls: 4/49<br>Pramipexole: 0/25
	Valvulopathy (valvular regurgitation)	Controls: number of events not reported (49 patients in group)<br>Pramipexole: 6/25
	Heart failure	Controls: 0/49<br>Ropinirole: 0/8
	Syncope	Controls: 4/49<br>Ropinirole: 0/8
Titlic, 2008 [37]	Orthostatic hypotension	Levodopa: 0/52<br>Ropinirole: 2/50
Yamamoto, 2006 [42]	Valvulopathy	**Abnormal regurgitation**<br>Control: 15/85<br>Pramipexole: 4/16<br><br>**Aortic regurgitation**<br>Control: 5/85<br>Pramipexole: 2/16<br><br>**Mitral regurgitation**<br>Control: 6/85<br>Pramipexole: 1/16<br><br>**Tricuspid regurgitation**<br>Control: 8/85<br>Pramipexole: 3/16<br><br>**Valve thickness**<br>Control: 18/85<br>Pramipexole: 4/16<br><br>**Valve calcification**<br>Control: 19/85<br>Pramipexole: 2/16
Junghanns, 2007 [12]	Valvulopathy	Ropinirole: 4/13<br>Pergolide: 16/25<br>Cabergoline: 15/24

Reference	Outcome	Treatment: Number of patients with event/number of patients in group
Yamashiro, 2008 [43]	Valvulopathy	**Tricuspid regurgitation, under 70 years**<br>Control: 0/53<br>Pramipexole: 1/37<br>Pergolide: 6/143<br>Cabergoline: 4/100<br>Bromocriptine: 0/20<br>Talipexole: 0/22<br><br>**Mitral regurgitation, under 70 years**<br>Control: 1/53<br>Pramipexole: 0/37<br>Pergolide: 3/143<br>Cabergoline: 2/100<br>Bromocriptine: 0/20<br>Talipexole: 0/22<br><br>**Aortic regurgitation, under 70 years**<br>Control: 1/53<br>Pramipexole: 0/37<br>Pergolide: 5/143<br>Cabergoline: 9/100<br>Bromocriptine:1/20<br>Talipexole: 0/22<br><br>**Tricuspid regurgitation, 70+ years**<br>Control: 2/26<br>Pramipexole: 1/14<br>Pergolide: 2/51<br>Cabergoline: 6/53<br>Bromocriptine: 0/8<br>Talipexole: 0/14<br><br>**Mitral regurgitation, 70+ years**<br>Control: 1/26<br>Pramipexole: 0/14<br>Pergolide: 0/51<br>Cabergoline: 3/53<br>Bromocriptine: 0/8<br>Talipexole: 1/14<br><br>**Aortic regurgitation, 70+ year**<br>Control: 1/26<br>Pramipexole: 2/14<br>Pergolide: 7/51<br>Cabergoline: 12/53<br>Bromocriptine: 2/8<br>Talipexole: 3/14

Table S5.2. Findings from RCTs that could not be included in the meta-analyses.

Comparison	Reference	Outcome	Proportion of patients with event	OR (95% CI)
Apomorphine vs. Placebo	Grosset, 2013 [7]	Hypertension	Apomorphine (treatment): 5/40; Placebo (control): 0/15.	4.80 [0.25,92.31]
Piribedil vs. Bromocriptine	Castro Caldas, 2006 [5]	Syncope	Piribedil (treatment): 5/210; Bromocriptine (control): 8/215.	0.63 [0.20,1.96]
		Hypertension	Piribedil (treatment): 15/210; Bromocriptine (control): 9/215.	1.76 [0.75,4.12]
		Hypotension	Piribedil (treatment): 16/210; Bromocriptine (control): 20/215.	0.80 [0.40,1.60]
		Peripheral Edema	Piribedil (treatment): 10/210; Bromocriptine (control): 10/215.	1.02 [0.42,2.52]
Piribedil vs. Placebo	Rascol, 2006 [30]	Hypertension	Piribedil (treatment): 19/200; Placebo (control): 9/205.	2.29 [1.01,5.181]
		Peripheral Edema	Piribedil (treatment): 10/200; Placebo (control): 7/205.	1.49 [0.56,3.99]
Pramipexole ER vs. Placebo	Schapira, 2011 [33]	Hypotension [Orthostatic Hypotension (Symptomatic and Asymptomatic)]	Pramipexole ER (treatment): 3/164; Placebo (control): 2/178.	1.64 [0.27,9.94]
Pramipexole IR vs. Pramipexole ER	Wang, 2014 [39]	Hypotension	Pramipexole IR (treatment): 5/239; Pramipexole ER (control).	4.98 [0.58,42.94]
Pramipexole vs. Bromocriptine	Guttman, 1997 [8]	Hypotension (Symptomatic and Asymptomatic Orthostatic Hypotension)	Pramipexole (treatment): 32/80; Bromocriptine (control): 37/84.	0.85 [0.46,1.58]
Pramipexole vs. Levodopa	Holloway, 2004 [9]	Peripheral Edema	Pramipexole (treatment): 7/151; Levodopa (control): 2/150.	3.60 [0.73,17.61]

Comparison	Reference	Outcome	Proportion of patients with event	OR (95% CI)
Pramipexole vs. Pardoprunox	Sampaio, 2011 [32]	Peripheral Edema	Pramipexole (treatment): 13/116; Pardoprunox (control): 3/108.	4.42 [1.22,15.96]
Pramipexole vs. Pergolide	Navan, 2003 [22]	Hypotension (Symptomatic Orthostatic)	Pramipexole (treatment): 0/10; Pergolide (control): 0/10.	Not estimable
Pramipexole vs. Placebo	Hubble, 1995 [10]	Hypertension (Asymptomatic Orthostatic Hypertension)	Pramipexole (treatment): 28/28; Placebo (control): 27/27.	Not estimable
		Hypertension (Symptomatic Orthostatic Hypertension)	Pramipexole (treatment): 7/28; Placebo (control): 5/27.	1.47 [0.40,5.35]
	Wermuth, 1998 [41]	Hypertension	Pramipexole (treatment): 0/36; Placebo (control): 1/33.	0.30 [0.01,7.54]
Pramipexole vs. Rasagiline	Viallet, 2013 [38]	Syncope	Pramipexole (treatment): 6/56; Rasagiline (control): 2/53.	3.06 [0.59,15.89]
		Hypotension (Symptomatic and Asymptomatic Orthostatic hypotension)	Pramipexole (treatment): 3/56; Rasagiline (control): 1/53.	2.94 [0.30,29.22]
Pramipexole vs. Ropinirole	Seiple, 2016 [35]	Hypotension (Symptomatic and Asymptomatic Orthostatic hypotension)	Pramipexole (treatment): 10/121; Ropinirole (control): 14/125.	0.71 [0.30,1.68]
		Peripheral Edema	Pramipexole (treatment): 22/121; Ropinirole (control): 18/125.	1.32 [0.67,2.61]
Pramipexole vs. Rotigotine	Poewe, 2007 [26]	Hypotension (Symptomatic and Asymptomatic Orthostatic Hypotension)	Pramipexole (treatment): 10/201; Rotigotine (G3) (control): 7/204.	1.47 [0.55,3.95]
Ropinirole IR vs. Ropinirole PR	Stocchi, 2011 [36]	Hypotension (Symptomatic and Asymptomatic Orthostatic Hypotension)	Ropinirole IR (treatment): 9/173; Ropinirole PR (control): 3/177.	3.18 [0.85,11.96]
		Hypotension	Ropinirole IR (treatment): 4/173; Ropinirole PR (control): 8/177.	0.50 [0.15,1.69]

Comparison	Reference	Outcome	Proportion of patients with event	OR (95% CI)
Ropinirole PR vs. Placebo	Zhang, 2013 [45]	Hypotension (Symptomatic and Asymptomatic Orthostatic Hypotension)	Ropinirole PR (treatment): 6/175; Placebo (control): 7/170.	0.83 [0.27,2.51]
		Hypotension	Ropinirole PR (treatment): 4/175; Placebo (control): 1/170.	3.95 [0.44,35.73]
		Peripheral Edema	Ropinirole PR (treatment): 5/175; Placebo (control): 4/170.	1.22 [0.32,4.62]
Ropinirole vs. Bromocriptine	Korczyn, 1999 [15]	Heart Failure	Ropinirole (treatment): 2/168; Bromocriptine (control): 5/167.	0.39 [0.07,2.04]
		Myocardial Infarction	Ropinirole (treatment): 1/168; Bromocriptine (control): 4/167.	0.24 [0.03,2.21]
Ropinirole vs. Levodopa	Rascol, 1998 [28]	Syncope	Ropinirole (treatment): 2/179; Levodopa (control): 1/89.	0.99 [0.09, 11.12]
	Rascol, 2000 [29]	Hypotension (Symptomatic and Asymptomatic Orthostatic Hypotension)	Ropinirole (treatment): 21/179; Levodopa (control): 11/89.	0.94 [0.43,2.05]
Ropinirole vs. Placebo	Pahwa, 2007 [24]	Syncope	Ropinirole (treatment): 1/202; Placebo (control): 0/191.	2.85 [0.12,70.42]
Ropinirole vs. Placebo	Mizuno, 2014 [19]	Valvulopathy	Ropinirole (treatment): 0/167; Placebo (control): 0/85.	Not estimable
		Peripheral Edema	Ropinirole (treatment): 2/167; Placebo (control): 3/85.	0.33 [0.05,2.02]
Ropinirole PR vs. Placebo	Zhang, 2013 [45]	Arrhythmia	Ropinirole PR (treatment): 1/175; Placebo (control): 1/170.	0.97 [0.06,15.65]
Rotigotine vs. Placebo	Poewe, 2007 [26]	Hypotension (Symptomatic Orthostatic)	Rotigotine (treatment): 1/205; Placebo (control): 1/99.	0.48 [0.03,7.76]
	Mizuno, 2014 [19]	Valvulopathy	Rotigotine (treatment): 0/168; Placebo (control): 0/85.	Not estimable
Rotigotine vs. Ropinirole	Mizuno, 2014 [19]	Valvulopathy	Rotigotine (treatment): 0/168; Ropinirole (control): 0/167.	Not estimable

Comparison	Reference	Outcome	Proportion of patients with event	OR (95% CI)
		Hypotension (Symptomatic and Asymptomatic Orthostatic Hypotension)	Rotigotine (treatment): 5/168; Ropinirole (control): 7/167.	0.70 [0.22,2.25]
		Peripheral Edema	Rotigotine (treatment): 0/168; Ropinirole (control): 2/167.	0.20 [0.01,4.12]

Table S5.3. Findings from case–control studies that could not be included in the meta-analyses.

Comparison	Reference	Outcome	Effect estimate (95% CI)
Pramipexole vs. all other DAs	Renoux, 2012 [31]	Heart Failure	RR 1.28 (0.82-2.00)
Pramipexole vs. ergot DAs	Renoux, 2012 [31]	Heart Failure	RR 1.07 (0.66-1.74)
Pramipexole vs. levodopa only use	Crispo, 2016 [6]	Hypotension	OR 1.09 (0.61-1.96)
		Myocardial Infarction	OR 0.88 (0.44-1.77)
		Stroke	OR 1.08 (0.50-2.30)
		Valvulopathy	OR 1.09 (0.68-1.75)
Pramipexole vs. no use of non-ergot DA	Crispo, 2016 [6]	Hypotension	OR 1.12 (0.80-1.44)
		Myocardial	OR 0.68 (0.46-1.01)
		Stroke	OR 1.09 (0.72-1.66)
Pramipexole vs. non-ergot DAs	Renoux, 2012 [31]	Heart Failure	RR 1.53 (0.92-2.57)
Ropinirole vs. levodopa only use	Crispo, 2016 [6]	Hypotension	OR 0.81 (0.49-1.35)
		Myocardial	OR 1.09 (0.52-2.29)
		Stroke	OR 0.52 (0.20-1.36)
		Valvulopathy	OR 1.73 (0.98-3.08)
Ropinirole vs. no use of non-ergot DA	Crispo, 2016 [6]	Hypotension	OR 1.02 (0.79-1.32)
		Myocardial	OR 0.98 (0.68-1.42)
		Stroke	OR 0.67 (0.41-1.09)
		Valvulopathy	OR 1.08 (0.82-1.43)

Supplementary Material S6. List of excluded studies.

Reference	Reason for exclusion
Apraxine, M.; Pasquet, A.; Jeanjean, A. Pramipexole-Induced Reversible Heart Failure. Mov Disord Clin Pract 2014, 1, 381-382, doi:10.1002/mdc3.12096.	Study type: case report.
Attanasio, A.; Capria, A.; Leggiadro, G.; Michisanti, M.; Cannata, D.; Stocchi, F.; Ruggieri, S. Transient cardiac arrest during continuous intravenous infusion of apomorphine. Lancet 1990, 336, 1321, doi:10.1016/0140-6736(90)93006-b.	Study type: case report.
Bares, M.; Rektorová, I.; Krajcovicová, L.; Rektor, I. Heart valve abnormalities in Parkinson's disease treated with dopamine agonists. J Neurol 2008, 255, 1596; author reply 1597, doi:10.1007/s00415-008-0972-x.	Study type: controlled before-and-after trial.
Biglan, K.M.; Holloway, R.G., Jr.; McDermott, M.P.; Richard, I.H. Risk factors for somnolence, edema, and hallucinations in early Parkinson disease. Neurology 2007, 69, 187-195, doi:10.1212/01.wnl.0000265593.34438.00.	Study type: post hoc analysis.
Bondon-Guitton, E.; Perez-Lloret, S.; Rascol, O.; Montastruc, J.L. Adverse drug reactions to dopaminergic agonists in the french pharmacovigilance database. Drug Safety 2009, 32(10), 888, doi:http://dx.doi.org/10.2165/11316660-000000000-00000.	Study type: disproportionality analyses.
Bostan, S.; Durmaz Celik, N.; Ozkan, S. Frequency and risk factors of dopamine agonist-induced peripheral edema in patients with Parkinson's disease. Movement Disorders 2020, 35(SUPPL 1), S457, doi:https://dx.doi.org/10.1002/mds.28268.	Duplicate citation.
Capecci, M.; Andrenelli, E.; Sordoni, E.; Monsu, A.M.; Di Biagio, L.; Ceravolo, M.G. Safety and tolerability of rotigotine transdermal system in patients over 70 years with advanced Parkinson's disease. Movement Disorders 2014, 1), S136, doi:http://dx.doi.org/10.1002/mds.25914.	Comparison: no comparator group.
Castaigne, P.; Laplane, D.; Dordain, G. Clinical experimentation with apomorphine in Parkinson's disease. Res Commun Chem Pathol Pharmacol 1971, 2, 154-158.	Comparison: no comparisons with other interventions.
Ceballos-Baumann, A.; Hck, H.J. Rotigotine transdermal patch in combination therapy for Parkinson's disease observations in routine clinical practice. Current Medical Research and Opinion 2011, 27(10), 1899-1905, doi:http://dx.doi.org/10.1185/03007995.2011.611630.	Comparison: no comparator group.
Chen, J.J.; Fernandez, H.H. Community and long-term care management of Parkinson's disease in the elderly: Focus on monoamine oxidase type B inhibitors. Drugs and Aging 2007, 24(8), 663-680, doi:http://dx.doi.org/10.2165/00002512-200724080-00004.	Study type: review article.
Corsini, G.U.; Del Zompo, M.; Gessa, G.L.; Mangoni, A. Therapeutic efficacy of apomorphine combined with an extracerebral inhibitor of dopamine receptors in Parkinson's disease. Lancet 1979, 1, 954-956.	Comparison: apomorphine in both treatment arms.
Dewey, R.B., Jr.; Maraganore, D.M.; Ahlskog, J.E.; Matsumoto, J.Y. Intranasal apomorphine rescue therapy for parkinsonian "off" periods. Clin Neuropharmacol 1996, 19, 193-201, doi:10.1097/00002826-199619030-00001.	Study type: controlled before-and-after trial.

Reference	Reason for exclusion
Dewey, R.B., 2nd; Reimold, S.C.; O'Suilleabhain, P.E. Cardiac valve regurgitation with pergolide compared with nonergot agonists in Parkinson disease. Archives of Neurology 2007, 64, 377-380.	Comparison: no comparator group.
Di Giacopo, R.; Fasano, A.; Fenici, R.; Loria, G.; Bentivoglio, A.R. Rare and serious cardiac side effects during ropinirole titration. Movement Disorders 2010, 25, 1509-1510, doi:https://dx.doi.org/10.1002/mds.23115.	Study type: open-label crossover trial.
Djaldetti, R.; Giladi, N.; Hassin-Baer, S.; Shabtai, H.; Melamed, E. Pharmacokinetics of Etilevodopa Compared to Levodopa in Patient's with Parkinson's Disease: An Open-label, Randomized, Crossover Study. Clinical Neuropharmacology 2003, 26(6), 322-326, doi:http://dx.doi.org/10.1097/00002826-200311000-00012.	Population: patients described as having Parkinsonism and not Parkinson's disease.
Duby, S.E.; Dahl, L.K.; Cotzias, G.C. Coupling of hypotensive and anit-Parkinson effects with two dopaminergic drugs. Trans Assoc Am Physicians 1971, 84, 289-296.	Comparison: open-label study, no comparator group.
Erken Pamukcu, H.; Gerede Uludag, D.M.; Tekin Tak, B.; Sorgun, M.H.; Efe, T.H.; Acibuca, A.; Akbostanci, C.; Turhan, S. Evaluation of the effect of non-ergot dopamine agonists on left ventricular systolic function with speckle tracking echocardiography. Anatolian Journal of Cardiology 2018, 20, 213-219, doi:https://dx.doi.org/10.14744/AnatolJCardiol.2018.65983.	Outcome: no outcome of interest.
Elmer, L.W.; Surmann, E.; Boroojerdi, B.; Jankovic, J. Long-term safety and tolerability of rotigotine transdermal system in patients with early-stage idiopathic Parkinson's disease: A prospective, open-label extension study. Parkinsonism and Related Disorders 2012, 18(5), 488-493, doi:http://dx.doi.org/10.1016/j.parkreldis.2012.01.008.	Duplicate citation.
Erken Pamukcu, H.; Gerede Uludag, D.M.; Tekin Tak, B.; Sorgun, M.H.; Efe, T.H.; Acibuca, A.; Akbostanci, C.; Turhan, S. Evaluation of the effect of non-ergot dopamine agonists on left ventricular systolic function with speckle tracking echocardiography. Anatolian journal of cardiology 2018, 20(4), 213-219, doi:http://dx.doi.org/10.14744/AnatolJCardiol.2018.65983.	Comparison: no comparator group.
Evidente, V.G.H.; Esteban, R.P.; Domingo, F.M.; Carbajal, L.O.; Parazo, M.A. Piribedil as an adjunct to levodopa in advanced Parkinson's disease: The Asian experience. Parkinsonism and Related Disorders 2003, 10(2), 117-121, doi:http://dx.doi.org/10.1016/S1353-8020%2803%2900096-8.	Study type: controlled before-and-after trial.
Faddoul, L.; Chahine, B.; Haydar, S.; Abourida, S.; Hallit, S.; Bou Raad, E. The effect of pramipexole extended release on the levodopa equivalent daily dose in Lebanese Parkinson diseased patients. Pharmacy Practice (1886-3655) 2018, 16, 1-5, doi:10.18549/PharmPract.2018.04.1220.	Study type: case report.
Famularo, G.; Minisola, G.; De Simone, C.; Nicotra, G.C. Vasculitis and nephritis caused by pramipexole, a second generation dopamine agonist. Clin Exp Rheumatol 2004, 22, 785.	Comparison: no comparator group.
Fasano, A.; Guidubaldi, A.; De Nigris, F.; Bentivoglio, A.R. Safety and efficacy of rotigotine in individuals with Parkinson's disease aged 75 and older. Journal of the American Geriatrics Society 2011, 59(12), 2386-2387, doi:http://dx.doi.org/10.1111/j.1532-5415.2011.03689.x.	Comparison: no comparator group.
Fernandez, H.H.; Isaacson, S.; Espay, A.J.; Pahwa, R.; Truong, D.; Pappert, E.; Agro, A.; Hauser, R.A. Safety of sublingual apomorphine film (APL-	Study type: case series.

Reference	Reason for exclusion
130277) for the treatment of off-episodes in subjects with Parkinson's disease: Preliminary results from the phase-3 study dose titration phase. Parkinsonism and Related Disorders 2018, 46(Supplement 2), e82.	
Giladi, N.; Surmann, E.; Boroojerdi, B. The safety and efficacy of transdermal rotigotine over a 6-year period in patients with early-stage idiopathic Parkinson's disease. Movement Disorders 2011, 2), S128-S129, doi:http://dx.doi.org/10.1002/mds.23764.	Comparison: open-label study, no comparator group.
Giladi, N.; Boroojerdi, B.; Surmann, E. The safety and tolerability of rotigotine transdermal system over a 6-year period in patients with early-stage Parkinson's disease. Journal of Neural Transmission 2013, 120, 1321-1329, doi:https://dx.doi.org/10.1007/s00702-013-1001-5.	Comparison: open-label study, no comparator group.
Giladi, N.; Asgharnejad, M.; Bauer, L.; Grieger, F.; Boroojerdi, B. Rotigotine in combination with the MAO-B inhibitor selegiline in early Parkinson's disease: A post hoc analysis. Journal of Parkinson's Disease 2016, 6(2), 401-411, doi:http://dx.doi.org/10.3233/JPD-150758.	Study type: post hoc analysis.
Grossac, J.; Ruiz, S.; Bondon-Guitton, E.; Roux, F.E.; Fourcade, O.; Montastruc, J.L.; Geeraerts, T. Severe intracranial bleeding related to vitamin K antagonist-ropinirole interaction. Movement Disorders 2011, 26, 1962-1963, doi:https://dx.doi.org/10.1002/mds.23733.	Study type: case report.
Grosset, K.; Needleman, F.; Macphee, G.; Grosset, D. Switching from ergot to nonergot dopamine agonists in Parkinson's disease: A clinical series and five-drug dose conversion table. Movement Disorders 2004, 19(11), 1370-1374, doi:http://dx.doi.org/10.1002/mds.20210.	Study type: controlled before-and-after trial.
Grosset, K.A.; Malek, N.; Morgan, F.; Grosset, D.G. Phase IIa randomized double-blind, placebo-controlled study of inhaled apomorphine as acute challenge for rescuing 'off' periods in patients with established Parkinson's disease. European Journal of Neurology 2013, 20(11), 1445-1450, doi:http://dx.doi.org/10.1111/ene.12091.	Outcome: no outcome of interest.
Hauser, R.A.; Bronzova, J.; Sampaio, C.; Lang, A.E.; Rascol, O.; Theeuwes, A.; Van De Witte, S.V. Safety and tolerability of pardoprunox, a new partial dopamine agonist, in a randomized, controlled study of patients with advanced Parkinson's disease for the pardoprunox study group. European Neurology 2009, 62(1), 40-48, doi:http://dx.doi.org/10.1159/000216839.	Intervention: no intervention of interest.
Hauser, R.A.; Schapira, A.H.; Rascol, O.; Barone, P.; Mizuno, Y.; Salin, L.; Haaksma, M.; Juhel, N.; Poewe, W. Randomized, double-blind, multicenter evaluation of pramipexole extended release once daily in early Parkinson's disease. Movement Disorders 2010, 25, 2542-2549, doi:10.1002/mds.23317.	Outcome: no outcome of interest.
Hauser, R.A.; Reichmann, H.; Lew, M.; Asgharian, A.; Makumi, C.; Shulman, K.J. Long-term, open-label study of once-daily ropinirole prolonged release in early Parkinson's disease. International Journal of Neuroscience 2011, 121, 246-253, doi:https://dx.doi.org/10.3109/00207454.2010.546538.	Comparison: open-label study, no comparator group.
Hauser, R.A.; Schapira, A.H.; Barone, P.; Mizuno, Y.; Rascol, O.; Busse, M.; Debieuvre, C.; Fraessdorf, M.; Poewe, W. Long-term safety and sustained efficacy of extended-release pramipexole in early and advanced Parkinson's disease. Eur J Neurol 2014, 21, 736-743, doi:10.1111/ene.12375.	Comparison: open-label study, no comparator group.

Reference	Reason for exclusion
Hauser, R.; Goud, S.; Formella, A. Should Amantadine DR/ER be considered prior to device-aided therapies for parkinson's disease. Movement Disorder 2021, 36(SUPPL 1), S175, doi:http://dx.doi.org/10.1002/mds.28794.	Intervention: no intervention of interest.
Hersh, B.P.; Earl, N.L.; Hauser, R.A.; Stacy, M. Early treatment benefits of ropinirole prolonged release in Parkinson's disease patients with motor fluctuations. Movement Disorders 2010, 25(7), 927-931, doi:http://dx.doi.org/10.1002/mds.23040.	Study type: review of included RCT.
Hsieh, P.H.; Hsiao, F.Y. Risk of heart failure associated with dopamine agonists: a nested case-control study. Drugs & Aging 2013, 30, 739-745, doi:https://dx.doi.org/10.1007/s40266-013-0102-z.	Population: not an idiopathic Parkinson's disease patient population.
Hutton, J.T.; Metman, L.V.; Chase, T.N.; Juncos, J.L.; Koller, W.C.; Pahwa, R.; LeWitt, P.A.; Samii, A.; Tsui, J.K.C.; Calne, D.B.; et al. Transdermal dopaminergic D<inf>2</inf> receptor agonist therapy in Parkinson's disease with N-0923 TDS: A double-blind, placebo-controlled study. Movement Disorders 2001, 16(3), 459-463, doi:http://dx.doi.org/10.1002/mds.1085.	Intervention: no intervention of interest.
Hwang, H.; Norris, S.A. Managing Advanced Parkinson Disease. Journal of Geriatric Psychiatry and Neurology 2021, 34(4), 289-300, doi:http://dx.doi.org/10.1177/08919887211018277.	Study type: review article.
Isaacson, S.; Lew, M.; Ondo, W.; Pagan, F. Efficacy of apomorphine subcutaneous injections for the management of morning akinesia in Parkinson's disease. Movement Disorders 2015, 1), S94, doi:http://dx.doi.org/10.1002/mds.26295.	Study type: controlled before-and-after trial.
Isaacson, S. Efficacy of apomorphine subcutaneous injections for the management of morning akinesia in Parkinson's disease. European Journal of Neurology 2015, 1), 251, doi:http://dx.doi.org/10.1111/ene.12807.	Study type: controlled before-and-after trial.
Isaacson, S.; Hauser, R.; Espay, A.; Pahwa, R.; Truong, D.; Pappert, E.; Gardzinksi, P.; Dzyngel, B.; Agro, A.; Fernandez, H. Safety of sublingual apomorphine film (APL-130277) for the treatment of OFF episodes in patients with Parkinson's disease: Preliminary results from a Phase III Study. Movement Disorders 2017, 32(Supplement 2), 899, doi:http://dx.doi.org/10.1002/mds.27087.	Duplicate citation.
Jeret, J.S. A multicenter trial of ropinirole as adjunct treatment for PD. 1999, 53, 658-658, doi:10.1212/wnl.53.3.656-c.	Study type: letter to the editor.
Jost, W.H.; Bellon, A.K.; Kaiser, T.; Schrank, B. The impact of ropinirole on blood pressure and noradrenaline concentration after active orthostasis in Parkinsonian patients. Parkinsonism Relat Disord 1998, 4, 61-63, doi:10.1016/s1353-8020(98)00014-5.	Population: not an idiopathic Parkinson's disease patient population.
Kim, J.M.; Chung, S.J.; Kim, J.W.; Jeon, B.S.; Singh, P.; Thierfelder, S.; Ikeda, J.; Bauer, L.; Asia Pacific Rotigotine Add-on Study, G. Rotigotine transdermal system as add-on to oral dopamine agonist in advanced Parkinson's disease: an open-label study. BMC Neurology 2015, 15, 17, doi:https://dx.doi.org/10.1186/s12883-015-0267-7.	Study type: non-randomized, open-label study.

Reference	Reason for exclusion
Korchounov, A.; Kessler, K.R.; Schipper, H.I. Differential effects of various treatment combinations on cardiovascular dysfunction in patients with Parkinson's disease. Acta Neurologica Scandinavica 2004, 109, 45-51.	Study type: controlled before-and-after trial.
Korczyn, A.D.; Brooks, D.J.; Brunt, E.R.; Poewe, W.H.; Rascol, O.; Stocchi, F. Ropinirole versus bromocriptine in the treatment of early Parkinson's disease: A 6-month interim report of a 3-year study. Movement Disorders 1998, 13(1), 46-51, doi:http://dx.doi.org/10.1002/mds.870130112.	Study type: interim analysis; retrieved full-length study.
Kujawa, K.; Leurgans, S.; Raman, R.; Blasucci, L.; Goetz, C.G. Acute orthostatic hypotension when starting dopamine agonists in Parkinson's disease. Archives of Neurology 2000, 57, 1461-1463.	Study type: controlled before-and-after trial.
Kuzu, M.; Özer, İ.Ş.; Herdi, O.; Tezcan, S.; Cenk Akbostancı, M. Switch from Immediate-release Pramipexole to Extended-release Pramipexole: The Safety and Efficacy Characteristics of Sixty-eight Patients. Turkish Journal of Neurology / Turk Noroloji Dergisi 2016, 22, 114-116, doi:10.4274/tnd.48751.	Study type: crossover trial.
Lees, A. Alternatives to levodopa in the initial treatment of early Parkinson's disease. Drugs & Aging 2005, 22, 731-740.	Study type: review article.
Lewitt, P.A.; Boroojerdi, B.; Surmann, E.; Poewe, W. Rotigotine transdermal system for long-term treatment of patients with advanced Parkinson's disease: Results of two open-label extension studies, CLEOPATRA-PD and PREFER. Journal of Neural Transmission 2013, 120(7), 1069-1081, doi:http://dx.doi.org/10.1007/s00702-012-0925-5.	Comparison: open-label study, no comparator group.
LeWitt, P.A.; Ondo, W.G.; Van Lunen, B.; Bottini, P.B. Open-label study assessment of safety and adverse effects of subcutaneous apomorphine injections in treating "off" episodes in advanced Parkinson disease. Clinical Neuropharmacology 2009, 32(2), 89-93, doi:http://dx.doi.org/10.1097/WNF.0b013e31816d91f9.	Comparison: open-label study, no comparator group.
Llau, M.E.; Durrieu, G.; Tran, M.A.; Senard, J.M.; Rascol, O.; Montastruc, J.L. A study of dopaminergic sensitivity in Parkinson's disease: comparison in "de novo" and levodopa-treated patients. Clinical Neuropharmacology 1996, 19, 420-427.	Comparison: no comparator group.
Lyon, G.J.; Shprecher, D.; Coffey, B.; Kurlan, R. Tourette's disorder. Current Treatment Options in Neurology 2010, 12(4), 274-286, doi:http://dx.doi.org/10.1007/s11940-010-0073-x.	Population: not an idiopathic Parkinson's disease patient population.
Lyons, K.E.; Pahwa, R. Ropinirole 24-h prolonged release in advanced Parkinson disease: Review of a randomized, double-blind, placebo-controlled study (EASE PD - Adjunct study). Progress in Neurotherapeutics and Neuropsychopharmacology 2008, 3(1), 73-84, doi:http://dx.doi.org/10.1017/S1748232107000122.	Study type: review of included RCT.
Lyons, K.E.; Friedman, J.H.; Hermanowicz, N.; Isaacson, S.H.; Hauser, R.A.; Hersh, B.P.; Silver, D.E.; Tetrud, J.W.; Elmer, L.W.; Parashos, S.A.; et al. Orally disintegrating selegiline in Parkinson patients with dopamine agonist-related adverse effects. Clin Neuropharmacol 2010, 33, 5-10, doi:10.1097/WNF.0b013e3181b7926f.	Intervention: open-label sutdy of selegiline.

Reference	Reason for exclusion
Makumi, C.W.; Asgharian, A.; Ellis, J.; Shaikh, S.; Jimenez, T.; VanMeter, S. Long-term, open-label, safety study of once-daily ropinirole extended/prolonged release in early and advanced Parkinson's disease. International Journal of Neuroscience 2016, 126, 30-38, doi:https://dx.doi.org/10.3109/00207454.2014.991924.	Comparison: extension study, no comparator group.
Malik, M.; Andreas, J.O.; Hnatkova, K.; Hoeckendorff, J.; Cawello, W.; Middle, M.; Horstmann, R.; Braun, M. Thorough QT/QTc study in patients with advanced Parkinson's disease: Cardiac safety of rotigotine. Clinical Pharmacology and Therapeutics 2008, 84(5), 595-603, doi:http://dx.doi.org/10.1038/clpt.2008.143.	Outcome: no outcome of interest.
Mandal, A.; Chatterjee, S.; Das, S.K.; Mishra, A. Drug safety monitoring in patients of movement disorders of a tertiary care hospital. Indian Journal of Pharmacology 2010, 42(4), 249-251, doi:http://dx.doi.org/10.4103/0253-7613.68437.	Population: not an idiopathic Parkinson's disease patient population.
Mizuno, Y.; Nomoto, M.; Kondo, T.; Hasegawa, K.; Murata, M.; Takeuchi, M.; Ikeda, J.; Tomida, T.; Hattori, N. Transdermal rotigotine in early stage Parkinson's disease: a randomized, double-blind, placebo-controlled trial. Movement Disorders 2013, 28, 1447-1450, doi:10.1002/mds.25537.	Outcome: no outcome of interest.
Mizuno, Y.; Nomoto, M.; Hasegawa, K.; Hattori, N.; Kondo, T.; Murata, M.; Takeuchi, M.; Takahashi, M.; Tomida, T. Rotigotine vs ropinirole in advanced stage Parkinson's disease: a double-blind study. Parkinsonism & Related Disorders 2014, 20, 1388-1393, doi:10.1016/j.parkreldis.2014.10.005.	Duplicate citation.
Montastruc, J.L.; Chaumerliac, C.; Desboeuf, K.; Manika, M.; Bagheri, H.; Rascol, O.; Lapeyre-Mestre, M. Adverse drug reactions to selegiline: A review of the French Pharmacovigilance Database. Clinical Neuropharmacology 2000, 23(5), 271-275, doi:http://dx.doi.org/10.1097/00002826-200009000-00006.	Outcome: no outcome of interest.
Montastruc, F.; Moulis, F.; Araujo, M.; Chebane, L.; Rascol, O.; Montastruc, J.L. Risk of heart failure with dopamine agonists in patients with Parkinson's disease: Differences between ergot and non-ergot derivatives. Fundamental and Clinical Pharmacology 2017, 31(Supplement 1), 11.	Study type: disproportionality analyses.
Montastruc, F.; Moulis, F.; Araujo, M.; Chebane, L.; Rascol, O.; Montastruc, J.-L. Ergot and non-ergot dopamine agonists and heart failure in patients with Parkinson's disease. European Journal of Clinical Pharmacology 2017, 73, 99-103, doi:10.1007/s00228-016-2142-x.	Study type: disproportionality analysis.
Morgante, L.; Basile, G.; Epifanio, A.; Spina, E.; Antonini, A.; Stocchi, F.; di Rosa, E.; Martino, G.; Marconi, R.; la Spina, P.; et al. Continuous apomorphine infusion (CAI) and neuropsychiatric disorders in patients with advanced Parkinson's disease: a follow-up of two years. Archives of Gerontology & Geriatrics 2004, 38, 291-296, doi:10.1016/j.archger.2004.04.039.	Outcome: no outcome of interest.
Muller, T.; Tolosa, E.; Badea, L.; Asgharnejad, M.; Grieger, F.; Markowitz, M.; Nondonfaz, X.; Bauer, L.; Timmermann, L. An observational study of rotigotine transdermal patch and other currently prescribed therapies in patients with Parkinson's disease. Journal of Neural Transmission 2018, 125, 953-963, doi:https://dx.doi.org/10.1007/s00702-018-1860-x.	Comparison: no comparator group.

Reference	Reason for exclusion
Navan, P.; Findley, L.J.; Jeffs, J.A.R.; Pearce, R.K.B.; Bain, P.G. Double-blind, single-dose, cross-over study of the effects of pramipexole, pergolide, and placebo on rest tremor and UPDRS part III in Parkinson's disease. Movement Disorders 2003, 18(2), 176-180, doi:http://dx.doi.org/10.1002/mds.10320.	Study type: crossover trial.
Nomoto, M.; Iwaki, H.; Kondo, H.; Sakurai, M. Efficacy and safety of rotigotine in elderly patients with Parkinson's disease in comparison with the non-elderly: a post hoc analysis of randomized, double-blind, placebo-controlled trials. Journal of Neurology 2018, 265(2), 253-265, doi:http://dx.doi.org/10.1007/s00415-017-8671-0.	Study type: post hoc analysis.
Oertel, W.; LeWitt, P.; Watts, R.; Grieger, F.; Bauer, L.; Boroojerdi, B. Treatment of patients with early and advanced Parkinson's disease with transdermal rotigotine: Safety and tolerability in elderly patients. Parkinsonism and Related Disorders 2009, 15(SUPPL 2), S129, doi:http://dx.doi.org/10.1016/S1353-8020%2809%2970498-5.	Study type: post hoc analysis.
Oertel, W.; Lewitt, P.; Giladi, N.; Ghys, L.; Grieger, F.; Boroojerdi, B.; Oertel, W.; LeWitt, P.; Giladi, N.; Ghys, L.; et al. Treatment of patients with early and advanced Parkinson's disease with rotigotine transdermal system: age-relationship to safety and tolerability. Parkinsonism & Related Disorders 2013, 19, 37-42, doi:10.1016/j.parkreldis.2012.06.009.	Study type: post hoc analysis.
Olanow, C.W.; Kieburtz, K.; Leinonen, M.; Elmer, L.; Giladi, N.; Hauser, R.A.; Klepiskaya, O.S.; Kreitzman, D.L.; Lew, M.F.; Russell, D.S.; et al. A randomized trial of a low-dose Rasagiline and Pramipexole combination (P2B001) in early Parkinson's disease. Movement Disorders 2017, 32(5), 783-789, doi:http://dx.doi.org/10.1002/mds.26941.	Intervention: no intervention of interest.
Olanow, C.; Kieburtz, K.; Livnah, N. Slow release fixed dose combination of low doses pramipexole and rasagiline (P2B001) for the treatment of early Parkinson's disease (PD). Movement Disorders 2017, 32(Supplement 2), 897, doi:http://dx.doi.org/10.1002/mds.27087.	Intervention: no intervention of interest.
Ondo, W.; Hunter, C.; Almaguer, M.; Jankovic, J. A novel sublingual apomorphine treatment for patients with fluctuating Parkinson's disease. Movement Disorders 1999, 14, 664-668.	Comparison: same study drug, different doses.
Ondo, W.; Hunter, C.; Almaguer, M.; Gancher, S.; Jankovic, J. Efficacy and tolerability of a novel sublingual apomorphine preparation in patients with fluctuating Parkinson's disease. Clin Neuropharmacol 1999, 22, 1-4, doi:10.1097/00002826-199901000-00001.	Study type: crossover trial.
Ondo, W.G.; Hunter, C.; Ferrara, J.M.; Mostile, G. Apomorphine injections: predictors of initial common adverse events and long term tolerability. Parkinsonism & Related Disorders 2012, 18, 619-622, doi:https://dx.doi.org/10.1016/j.parkreldis.2012.01.001.	Comparison: no comparator group.
Pahwa, R.; Koller, W.C.; Trosch, R.M.; Sherry, J.H.; Investigators, A.P.O.S. Subcutaneous apomorphine in patients with advanced Parkinson's disease: a dose-escalation study with randomized, double-blind, placebo-controlled crossover evaluation of a single dose. Journal of the Neurological Sciences 2007, 258, 137-143.	Study type: crossover trial.

Reference	Reason for exclusion
Parkinson Study Group. Safety and efficacy of pramipexole in early Parkinson disease. A randomized dose-ranging study. Parkinson Study Group. JAMA: Journal of the American Medical Association 1997, 278, 125-130, doi:10.1001/jama.278.2.125.	Outcome: no outcome of interest by comparison groups.
The Parkinson Study Group. The Parkinson study group: pramipexole as adjunctive treatment in African, Asian and Hispanic Parkinson's disease patients. Brown University Geriatric Psychopharmacology Update 2007, 11, 1-7.	Study type: review article.
Poewe, W.; Kleedorfer, B.; Wagner, M.; Benke, T.; Gasser, T.; Oertel, W. Side-effects of subcutaneous apomorphine in Parkinson's disease. 1989, 333, 1084-1085, doi:10.1016/s0140-6736(89)92487-2.	Study type: commentary.
Poewe, W.; Barone, P.; Hauser, R.; Mizuno, Y.; Rascol, O.; Busse, M.; Debieuvre, C.; Fraessdorf, M.; Schapira, A. Long-term safety and sustained efficacy of extended-release pramipexole in early and advanced Parkinson's disease. Movement Disorders 2011, 2), S137-S138, doi:http://dx.doi.org/10.1002/mds.23764.	Comparison: open-label study, no comparator group.
Poewe, W.; Rascol, O.; Barone, P.; Hauser, R.A.; Mizuno, Y.; Haaksma, M.; Salin, L.; Juhel, N.; Schapira, A.H. Extended-release pramipexole in early Parkinson disease: a 33-week randomized controlled trial. Neurology 2011, 77, 759-766, doi:10.1212/WNL.0b013e31822affb0.	Outcome: no outcome of interest.
Pollak, P.; Mallaret, M.; Gaio, J.M.; Hommel, M.; Perret, J. Blood pressure effects of apomorphine and domperidone in parkinsonism. Adv Neurol 1987, 45, 263-266.	Population: patients described as having Parkinsonism and not Parkinson's disease.
Rascol, O.; Azulay, J.P.; Blin, O.; Bonnet, A.M.; Brefel-Courbon, C.; Cesaro, P.; Damier, P.; Debilly, B.; Durif, F.; Galitzky, M.; et al. Orodispersible sublingual piribedil to abort OFF episodes: A single dose placebo-controlled, randomized, double-blind, cross-over study. Movement Disorders 2010, 25(3), 368-376, doi:http://dx.doi.org/10.1002/mds.22922.	Study type: crossover trial.
Roychowdhury, S.; Forsyth, D.R. Sleep disturbance in Parkinson disease. Journal of Clinical Gerontology and Geriatrics 2012, 3(2), 53-61, doi:http://dx.doi.org/10.1016/j.jcgg.2012.04.002.	Study type: review article.
Schapira, A.H.V.; Barone, P.; Hauser, R.A.; Mizuno, Y.; Rascol, O.; Busse, M.; Debieuvre, C.; Fraessdorf, M.; Poewe, W. Success rate, efficacy, and safety/tolerability of overnight switching from immediate- to extended-release pramipexole in advanced Parkinson's disease. European Journal of Neurology 2013, 20(1), 180-187, doi:http://dx.doi.org/10.1111/j.1468-1331.2012.03822.x.	Comparison: extension study, no comparator group.
Schrag, A.E.; Brooks, D.J.; Brunt, E.; Fuell, D.; Korczyn, A.; Poewe, W.; Quinn, N.P.; Rascol, O.; Stocchi, F. The safety of ropinirole, a selective nonergoline dopamine agonist, in patients with Parkinson's disease. Clinical Neuropharmacology 1998, 21(3), 169-175.	Study type: review article.
Secil, Y.; Eryasar, G.; Incesu, T.K. Less and well known side effects of dopamine agonists in Parkinson's disease: Comparison of ropinirole and pramipexole. Movement Disorders 2013, 1), S207, doi:http://dx.doi.org/10.1002/mds.25605.	Study type: cross-sectional study.

Reference	Reason for exclusion
Senol, M.G.; Togrol, R.E. Dopamine agonists and cardiac valvulopathy in Parkinson disease: a case-control study. Neurology 2007, 69, 117; author reply 117-118, doi:10.1212/01.wnl.0000270100.32701.7d.	Study type: letter to the editor.
Sha, K.; Kodama, T.; Yagi, N. Post-marketing surveillance of pramipexole extended release tablets in patients with Parkinson's disease. [Japanese]. Therapeutic Research 2015, 36(3), 251-258.	Comparison: no comparator group.
Sigurdardottir, G.R.; Nilsson, C.; Odin, P.; Grabowski, M. Cardiovascular effects of domperidone in patients with Parkinson's disease treated with apomorphine. Acta Neurologica Scandinavica 2001, 104, 92-96.	Comparison: no comparator group.
Stibe, C.; Lees, A.; Stern, G. Subcutaneous infusion of apomorphine and lisuride in the treatment of parkinsonian on-off fluctuations. 1987, 1, 871-871, doi:10.1016/s0140-6736(87)91660-6.	Study type: commentary.
Tan, E.K. Peripheral edema and dopamine agonists in Parkinson disease. Archives of Neurology 2007, 64, 1546-1547; author reply 1547.	Study type: commentary.
Trenkwalder, C.; Kies, B.; Rudzinska, M.; Fine, J.; Nikl, J.; Honczarenko, K.; Dioszeghy, P.; Hill, D.; Anderson, T.; Myllyla, V.; et al. Rotigotine effects on early morning motor function and sleep in Parkinson's disease: a double-blind, randomized, placebo-controlled study (RECOVER). Movement Disorders 2011, 26, 90-99, doi:10.1002/mds.23441.	Outcome: no outcome of interest.
Turkmen, C.; Ozen, B.; Ince Gunal, D. Pedal oedema in Parkinson's disease patients using dopamine agonists. European Journal of Neurology 2010, 3), 383, doi:http://dx.doi.org/10.1111/j.1468-1331.2010.03233.x.	Study type: cross-sectional study.
Rotigotine (Neupro) Drug Evaluation. Drug Formulary Review 2007, 23, 1-4.	Study type: review article.
Pramipexole: heart failure. Prescrire Int 2013, 22, 213.	Study type: review article.
Vermersch, P.; Mounier-Vehier, F.; Caron, J.; Salomez, J.L.; Petit, H. Severe oedema after subcutaneous apomorphine in Parkinson's disease. Lancet 1989, 2, 802.	Study type: case report.
Watts, R.; Pahwa, R.; Lyons, K.; Boroojerdi, B. Long-term safety of rotigotine transdermal patch in early-stage Parkinson's disease: Four year results. Parkinsonism and Related Disorders 2009, 15(SUPPL 2), S137, doi:http://dx.doi.org/10.1016/S1353-8020%2809%2970525-5.	Comparison: open-label study, no comparator group.
Watts, R.L.; Boroojerdi, B.; Jankovic, J. Open-label extension trial assessing the effects of long-term treatment with rotigotine in subjects with early-stage, idiopathic Parkinson's disease: Results from up to 7 years. Movement Disorders 2010, 2), S310-S311, doi:http://dx.doi.org/10.1002/mds.23162.	Comparison: open-label study, no comparator group.
Watts, R.L.; Lyons, K.E.; Pahwa, R.; Sethi, K.; Stern, M.; Hauser, R.A.; Olanow, W.; Gray, A.M.; Adams, B.; Earl, N.L. Onset of dyskinesia with adjunct ropinirole prolonged-release or additional levodopa in early Parkinson's disease. Movement Disorders 2010, 25, 858-866, doi:10.1002/mds.22890.	Outcome: no outcome of interest.
Weiner, W.J.; Factor, S.A.; Jankovic, J.; Hauser, R.A.; Tetrud, J.W.; Waters, C.H.; Shulman, L.M.; Glassman, P.M.; Beck, B.; Paume, D.; et al. The long-term safety and efficacy of pramipexole in advanced Parkinson's disease. Parkinsonism & Related Disorders 2001, 7, 115-120.	Comparison: open-label study, no comparator group.

Reference	Reason for exclusion
Whone, A.L.; Watts, R.L.; Stoessl, A.J.; Davis, M.; Reske, S.; Nahmias, C.; Lang, A.E.; Rascol, O.; Ribeiro, M.J.; Remy, P.; et al. Slower progression of Parkinson's disease with ropinirole versus levodopa: the REAL-PET study. Annals of Neurology 2003, 54, 93-101.	Outcome: no outcome of interest.
Wong, K.S.; Lu, C.S.; Shan, D.E.; Yang, C.C.; Tsoi, T.H.; Mok, V. Efficacy, safety, and tolerability of pramipexole in untreated and levodopa-treated patients with Parkinson's disease. Journal of the Neurological Sciences 2003, 216(1), 81-87, doi:http://dx.doi.org/10.1016/S0022-510X%2803%2900217-X.	Outcome: no outcome of interest.
Yoshii, F.; Motoyama, H. Efficacy and safety of long-term and high-dose treatment with ropinirole (ROP) in Japanese patients with Parkinson's disease: LEAD-PD study. Therapeutic Research 2011, 32(8), 1033-1046.	Comparison: no comparator group.

Supplementary Material S7. Characteristics of studies awaiting classification.

Study	Methods	Participants	Interventions	Outcomes	Notes
Barzola, 2012 [47]	**Study type:** cohort	**Number of participants:** 110 with PD (71 patients on levodopa, 33 patients on dopamine)<br><br>**Age:** 65 years or older	**Treatment groups:**<br>• levodopa<br>• dopamine	• peripheral edema	• Abstract only
Bostan, 2020 [48]	**Study type:** retrospective cohort	**Number of participants:** 370 PD patients on dopamine agonists	**Treatment groups:**<br>• dopamine agonists	• peripheral edema	• Abstract only
Chiang, 2017 [49]	**Study type:** cohort<br><br>**Duration:** 6-month follow-up	**Number of participants:** 45 patients with advanced PD	**Treatment groups:**<br>• intermittent injections with apomorphine<br>• continuous subcutaneous infusion with apomorphine<br>• levodopa–carbidopa intestinal gel	• not reported in abstract	• Abstract only
Cvetkovska, 2012 [50]	**Study type:** RCT<br><br>**Duration:** 6 months	**Number of participants:** 21 newly diagnosed PD patients<br><br>**Age:** range in years = 52-74	**Treatment groups:**<br>• pramipexole ER (1.5 mg/24 hr)<br>• pramipexole IR (1.5 mg/24 hr)	• edema	• Study treatment arms were unclear in the abstract
Dafsari, 2019 [51]	**Study type:** prospective cohort<br><br>**Duration:** 6-month follow-up	**Number of participants:** 173 patients with PD<br><br>**Mean age in years (SD):**<br>• STN-DBS: 61.5 (9.5)<br>• IJLI: 65.4 (8.8)<br>• APO: 61.6 (9.7)	**Treatment groups:**<br>• STN-DBS or other targets<br>• IJLI<br>• APO	• orthostatic hypertension	• Eligible for inclusion in a future update of this review

Study	Methods	Participants	Interventions	Outcomes	Notes
Factor, 2020 [52]	**Study type:** RCT<br><br>**Duration:** 12 weeks		• sublingual film apomorphine	• adverse events<br>• syncope	• Abstract only
Gehlen, 1980 [53]	unknown	unknown	unknown	unknown	• Article published in non-English language
Gencler, 2022 [54]	**Study type:** RCT<br><br>**Duration:** 6 months	**Number of participants:** 44 idiopathic PD patients<br><br>**Mean age years (sd):**<br>• pramipexole + DAMG: 60.9 ± 13.7<br>• ropinirole + DAMG: 62.6 ± 11.5<br>• pramipexole + LAG: 69.8 ± 5.9<br>• ropinirole + LAG 66.0 ± 10.6	**Treatment groups:**<br>• pramipexole + DAMG<br>• ropinirole + DAMG<br>• pramipexole + LAG<br>• ropinirole + LAG	• orthostatic hypotension	• Eligible for inclusion in a future update of this review
Hauser, 2016 [55]	**Study type:** RCT<br><br>**Duration:** 29 weeks	**Number of participants:** 122 PD patients<br><br>**Mean age in years (SD):**<br>• placebo: 69.0 (11.7)<br>• low-dose rotigotine: 68.1 (10.5)<br>• high-dose rotigotine: 70.2 (8.0)	**Treatment groups:**<br>• placebo<br>• low-dose rotigotine (transdermal patch)<br>• high-dose rotigotine (transdermal patch)	• peripheral edema	• Eligible for inclusion in a future update of this review
Isaacson, 2017 [56]	**Study type:** RCT	**Number of participants:** 96 PD patients	**Treatment groups:**<br>• placebo<br>• sublingual apomorphine film	• adverse events<br>• blood pressure	• Abstract only

Study	Methods	Participants	Interventions	Outcomes	Notes
Li, 2013 [57]	**Study type:** RCT<br><br>**Duration:** 12 weeks	**Number of participants:** 221 PD patients	**Treatment groups:**<br>• ropinirole<br>• bromocriptine	• adverse events<br>• blood pressure	• Article published in non-English language
Oeda, 2009 [58]	**Study type:** case–control	**Number of participants:** 223 enrolled (patients without VHD =115; patients with VHD =108)<br><br>**% Female:** patients without VHD: 60%; patients with VHD: 58.3%<br><br>**Mean age in years (SD):** without VHD: 68.3 (9.4); with VHD: 72.7 (9.6)<br><br>**Mean PD duration in years (SD):** without VHD: 8.2 (5.7); with VHD: 10.7 (6.0)<br><br>**Inclusion criteria:**<br>• had been treated for PD for 3 or more years<br>• informed consent<br><br>**Exclusion criteria:**<br>• bicuspid aortic valves, mitral valve prolapse, and findings suggestive of myocardial infarction, such as hypokinesia of the ventricular walls	**Treatment groups:**<br>• cabergoline (cumulative dose mean 2,120 mg)<br>• pergolide (cumulative dose mean 739 mg)<br>• bromocriptine (cumulative dose mean 12,700 mg)<br>• pramipexole (cumulative dose mean 795 mg)	• valvulopathy	• Quantitative findings are not reported

Study	Methods	Participants	Interventions	Outcomes	Notes
		• patients with a history of myxomatous degeneration of the heart valves			
Olanow, 2020 [59]	**Study type:** RCT<br><br>**Duration:** 12 weeks	**Number of participants:** 109 PD patients<br><br>**Mean age in years (SD):**<br>• placebo: 62.5 (8.12)<br>• apomorphine sublingual film: 62.9 (9.79	**Treatment groups:**<br>• placebo<br>• sublingual apomorphine film	• orthostatic hypotension<br>• syncope	• Eligible for inclusion in a future update of this review
Pahwa, 2018 [60]	**Study type:** RCT<br><br>**Duration:** 12 weeks	**Number of participants:** 141 PD patients	**Treatment groups:**<br>• placebo<br>• sublingual apomorphine film	• adverse events<br>• vital signs<br>• electrocardiogram<br>• laboratory tests	• Abstract only
Parkinson Study Group, 2000 [61]	**Study type:** RCT<br><br>**Duration:** 23.5 months	**Number of participants:** 301 early-PD patients<br><br>**Mean age in years (SD):**<br>• pramipexole: 61.5 (10.1)<br>• levodopa: 60.9 (10.5)	**Treatment groups:**<br>• pramipexole with levodopa placebo<br>• carbidopa/levodopa with pramipexole placebo	• peripheral edema<br>• syncope	• Eligible for inclusion in a future update of this review
Pinter, 1999 [62]	**Study type:** RCT<br><br>**Duration:** 12 weeks	**Number of participants:** 78 patients with advanced PD<br><br>**Mean age in years (SD):**<br>• placebo: 60.7 (8.7)<br>• pramipexole: 59.3 (8.3)	**Treatment groups:**<br>• placebo<br>• pramipexole	• postural hypotension	• Eligible for inclusion in a future update of this review
Pogarell, 2002 [63]	**Study type:** RCT<br><br>**Duration:** 12 weeks	**Number of participants:** 84 PD patients<br><br>**Mean age in years (SD):**<br>• placebo: 65.4 (7.1)<br>• pramipexole: 62.0 (10.1)	**Treatment groups:**<br>• placebo<br>• pramipexole	• postural hypotension	• Eligible for inclusion in a future update of this review

Study	Methods	Participants	Interventions	Outcomes	Notes
Tao, 2019 [64]	**Study type:** retrospective cohort<br><br>**Duration:** 3 years	**Number of participants:** 500 PD patients<br><br>**Mean age in years (SD):**<br>• pramipexole: 65.52 (7.57)<br>• selegiline: 64.59 (8.11)	**Treatment groups:**<br>• pramipexole<br>• selegiline	• peripheral edema	• Eligible for inclusion in a future update of this review
Thijssen, 2020 [65]	**Study type:** RCT	**Number of participants:** 24 PD patients<br><br>**Age:** range in years = 30-85	**Treatment groups:**<br>• placebo<br>• inhaled apomorphine	• orthostatic hypotension<br>• syncope	• Eligible for inclusion in a future update of this review
Wen, 2006 [66]	**Study type:** RCT<br><br>**Duration:** 12 weeks	**Number of participants:** 208 PD patients	**Treatment groups:**<br>• control<br>• pramipexole<br>• bromocriptine	• safety outcomes<br>• blood pressure	• Study in non-English language
Zhang, 2016 [67]	**Study type:** RCT<br><br>**Duration:** 28 weeks	**Number of participants:** 247 early-PD patients<br><br>**Mean age in years (SD):**<br>• placebo: 59.7 (10.1)<br>• transdermal rotigotine: 59.1 (10.3)	**Treatment groups:**<br>• placebo<br>• transdermal rotigotine	• hypotension	• Eligible for inclusion in a future update of this review
Zhang, 2017 [68]	**Study type:** RCT<br><br>**Duration:** 19 weeks	**Number of participants:** 346 patients with advanced PD<br><br>**Mean age in years (SD):**<br>• placebo: 62.8 (9.1)<br>• transdermal rotigotine: 61.7 (8.8)	**Treatment groups:**<br>• placebo<br>• transdermal rotigotine	• syncope<br>• atrial fibrillation<br>• valvulopathy	• Eligible for inclusion in a future update of this review
Zheng, 2019 [69]	**Study type:** RCT	**Number of participants:** 388 PD patients	**Treatment groups:**<br>• pramipexole<br>• entacapone	• adverse events	• Study in non-English language

Abbreviations: APO: apomorphine infusion; DAG: dopamine agonist monotherapy group; IJLI: intrajejunal levodopa infusion; LAG: levodopa add-on therapy group; STN-DBS: deep brain stimulation of the subthalamic nucleus.

Supplementary Material S8. Risk of bias assessments for RCTs included in the review.

Study	Random sequence generation (selection bias)	Allocation concealment (selection bias)	Blinding of participants, personnel, and outcome assessors (performance bias and detection bias)	Incomplete outcome data (attrition bias)—all outcomes	Selective reporting (reporting bias)	Other bias
Barone, 2010 [3]	**Judgement:** Low risk.<br><br>**Support:** Quote: "The randomisation code was provided by the study sponsor using their validated, centralised, randomisation number generating system, and was stratified by study centre with a block size of four to provide a balanced distribution of the treatment groups within each centre and across the study as a whole."	**Judgement:** Low risk.<br><br>**Support:** Quote: "The randomisation code was provided by the study sponsor using their validated, centralised, randomisation number generating system, and was stratified by study centre with a block size of four to provide a balanced distribution of the treatment groups within each centre and across the study as a whole."	**Judgement:** Low risk.<br><br>**Support:** Quote: "To preserve masking, access to the randomisation code was restricted to clinical trial support and pharmaceutical personnel, who generated the code and labelled and packaged the study drugs... Pramipexole and matching placebo tablets were prepared by Boehringer Ingelheim, Germany, by use	**Judgement:** Low risk.<br><br>**Support:** Proportions of patients that discontinued treatment or placebo were similar.<br><br>Intention to treat analysis.	**Judgement:** Low risk.<br><br>**Support:** The outcome of interest was assessed as an adverse event.	**Judgement:** Low risk.<br><br>**Support:** None identified.

Study	Random sequence generation (selection bias)	Allocation concealment (selection bias)	Blinding of participants, personnel, and outcome assessors (performance bias and detection bias)	Incomplete outcome data (attrition bias)—all outcomes	Selective reporting (reporting bias)	Other bias
			of the same excipients, such that the tablets could not be differentiated."			
Blindeauer, 2003 [4]	**Judgement:** Low risk.<br><br>**Support:** Quote: "The computer-generated randomized plan included stratification by centre and blocking to ensure approximate balance among treatment groups within each center."	**Judgement:** Low risk.<br><br>**Support:** Quote: "Only designated staff members in the Biostatistics Center, Q- Tone IVRS, and Schwarz Pharma (Schwarz Pharma Manufacturing Inc, Seymour, Ind, and Schwarz Pharma Inc, Mequon, Wis), who packaged and labeled the drug, were potentially aware of the individual	**Judgement:** Low risk.<br><br>**Support:** Quote: "Only designated staff members in the Biostatistics Center, Q- Tone IVRS, and Schwarz Pharma (Schwarz Pharma Manufacturing Inc, Seymour, Ind, and Schwarz Pharma Inc, Mequon, Wis), who packaged and labeled the drug, were potentially aware of the individual	**Judgement:** Unclear risk.<br><br>**Support:** Intention to treat analysis.<br><br>Proportions of patients that discontinued treatment were not equal among treatment groups.	**Judgement:** Unclear risk.<br><br>**Support:** Not reported.	**Judgement:** Low risk.<br><br>**Support:** None identified.

Study	Random sequence generation (selection bias)	Allocation concealment (selection bias)	Blinding of participants, personnel, and outcome assessors (performance bias and detection bias)	Incomplete outcome data (attrition bias)—all outcomes	Selective reporting (reporting bias)	Other bias
		treatment assignments."<br><br>Staff had to telephone an interactive voice response system to receive the appropriately assigned drug kit number.	treatment assignments."			
Castro-Caldas, 2006 [5]	**Judgement:** Unclear risk.<br><br>**Support:** Randomization of treatment not explained.	**Judgement:** Unclear risk.<br><br>**Support:** Not reported.	**Judgement:** Unclear risk.<br><br>**Support:** Not reported.	**Judgement:** Low risk.<br><br>**Support:** Intention to treat analysis.<br><br>Similar proportions of patients discontinued treatment in both study arms.	**Judgement:** Unclear risk.<br><br>**Support:** No protocol identified.	**Judgement:** Low risk.<br><br>**Support:** None identified.
Grosset, 2013 [7]	**Judgement:** Low risk.	**Judgement:** Unclear risk.	**Judgement:** Unclear risk.	**Judgement:** Unclear risk.	**Judgement:** Unclear risk.	**Judgement:** Low risk.

Study	Random sequence generation (selection bias)	Allocation concealment (selection bias)	Blinding of participants, personnel, and outcome assessors (performance bias and detection bias)	Incomplete outcome data (attrition bias)—all outcomes	Selective reporting (reporting bias)	Other bias
	**Support:**<br>Randomization was performed centrally via an interactive voice response system.	**Support:**<br>Not reported.	**Support:**<br>Not reported.	**Support:**<br>All patients accounted for in safety analysis.<br><br>Significantly higher proportion of patients on placebo compared to treatment did not complete the study.	**Support:**<br>Not reported.	**Support:**<br>None identified.
Guttman 1997 [8]	**Judgement:**<br>Unclear risk.<br><br>**Support:**<br>Not reported.	**Judgement:**<br>Unclear risk.<br><br>**Support:**<br>Not reported.	**Judgement:**<br>Unclear risk.<br><br>**Support:**<br>Not reported.	**Judgement:**<br>High risk.<br><br>**Support:**<br>There were 16 (20%) in the pramipexole group, 17 (20%) in the bromocriptine group, and 33 (40%) in the placebo group.	**Judgement:**<br>Unclear risk.<br><br>**Support:**<br>Not reported.	**Judgement:**<br>Low risk.<br><br>**Support:**<br>None identified.
Holloway, 2004 [9]	**Judgement:**<br>Low risk.	**Judgement:**<br>Low risk.	**Judgement:**<br>Low risk.	**Judgement:**<br>High risk.	**Judgement:**<br>Unclear risk.	**Judgement:**<br>Low risk.

Study	Random sequence generation (selection bias)	Allocation concealment (selection bias)	Blinding of participants, personnel, and outcome assessors (performance bias and detection bias)	Incomplete outcome data (attrition bias)—all outcomes	Selective reporting (reporting bias)	Other bias
	**Support:** Quote: "Eligible subjects were randomized with equal allocation to one of the two treatment groups with use of a computer-generated randomization plan that included stratification by investigator and blocking to ensure that each investigator had approximately the same number of subjects assigned to each treatment groups."	**Support:** Quote: "When a subject was judged eligible to be enrolled, a telephone call was made to the Coordination Center, which provided a unique subject identification number and treatment assignment from the randomization module. Access to the randomization code is restricted to specified programmers at Pharmacia & Upjohn and the PSG Biostatistics Center (Rochester, NY), but is otherwise	**Support:** Participants, study personnel, and the investigator assessing the primary endpoint at each site were blinded.	**Support:** 67/151 in pramipexole group and 49/150 in placebo group discontinued treatment. Higher proportion of discontinuations were due to somnolence in pramipexole group compared to placebo group.	**Support:** Not reported.	**Support:** None identified.

Study	Random sequence generation (selection bias)	Allocation concealment (selection bias)	Blinding of participants, personnel, and outcome assessors (performance bias and detection bias)	Incomplete outcome data (attrition bias)—all outcomes	Selective reporting (reporting bias)	Other bias
		concealed from all other study personnel."				
Hubble, 1995 [10]	**Judgement:** Unclear risk.<br><br>**Support:** Not reported.	**Judgement:** Unclear risk.<br><br>**Support:** Not reported.	**Judgement:** Low risk.<br><br>**Support:** Participants and study investigators were blinded as to whether active drug or placebo was dispensed and taken. Study coordinator was not blinded.	**Judgement:** Low risk.<br><br>**Support:** Similar proportions of patients discontinued treatment or placebo.	**Judgement:** Unclear risk.<br><br>**Support:** No study protocol identified.	**Judgement:** Low risk.<br><br>**Support:** None identified.
Im, 2003 [11]	**Judgement:** Unclear risk.<br><br>**Support:** Quote: "Stratified block randomisation method was applied according to the previous	**Judgement:** Unclear risk.<br><br>**Support:** Not reported.	**Judgement:** High risk.<br><br>**Support:** Open trial.<br><br>Quote: "Ideally, the trial would have been blinded, but	**Judgement:** Low risk.<br><br>**Support:** Intention to treat analysis used.<br><br>Similar proportions of patients	**Judgement:** Unclear risk.<br><br>**Support:** Not reported.	**Judgement:** Low risk.<br><br>**Support:** None identified.

Study	Random sequence generation (selection bias)	Allocation concealment (selection bias)	Blinding of participants, personnel, and outcome assessors (performance bias and detection bias)	Incomplete outcome data (attrition bias)—all outcomes	Selective reporting (reporting bias)	Other bias
	exposure to a dopamine agonist."<br><br>Details of randomization method not described.		importation of matched tablets of these drugs is difficult in Korea. Thus the present study was designed as an open comparative one, but with patients randomly assigned to the treatment groups."	discontinued treatment in both study arms.		
Katzenschlager, 2018 [13]	**Judgement:** Low risk.<br><br>**Support:** Patients were randomized to apomorphine or placebo using a central computer-generated randomization code generated by the Biometric Department of	**Judgement:** Low risk.<br><br>**Support:** Randomization was provided by a central computer-generated code.	**Judgement:** High risk.<br><br>**Support:** All study participants and investigators were masked to group assignment. Treatment and placebo infusions were similar in packaging, appearance, and weight. However,	**Judgement:** High risk.<br><br>**Support:** Unequal loss of patients from study arms: 12 patients on apomorphine and 24 patients from the placebo group did not complete the full 12-week double-blind phase.	**Judgement:** Unclear risk.<br><br>**Support:** Publicly available protocol (NCT02006121). No mention of monitoring adverse events.	**Judgement:** High risk.<br><br>**Support:** The funder, Britannia Pharmaceuticals, was involved in study design and was responsible for data collection, monitoring, and statistical analysis.

Study	Random sequence generation (selection bias)	Allocation concealment (selection bias)	Blinding of participants, personnel, and outcome assessors (performance bias and detection bias)	Incomplete outcome data (attrition bias)—all outcomes	Selective reporting (reporting bias)	Other bias
	Advanced Medical Services.		the authors suggest that blinding may have been compromised for both participants and assessors due to treatment-related reasons.			
Kieburtz, 2011 [14]	**Judgement:** Low risk.<br><br>**Support:** Quote: "A computer-generated randomization plan was provided by the study sponsor."	**Judgement:** Low risk.<br><br>**Support:** Quote: "Participant enrollment was implemented through an internet-accessible electronic data capture system."	**Judgement:** Low risk.<br><br>**Support:** Quote: "Only the personnel who generated the randomization plan, those involved in drug packaging, and a programmer had access to the participant treatment assignments until the trial database was locked for	**Judgement:** Low risk.<br><br>**Support:** Intention to treat analysis.<br><br>Similar proportions of patients in each study arm discontinued treatment.	**Judgement:** Unclear risk.<br><br>**Support:** Not reported.	**Judgement:** Low risk.<br><br>**Support:** None identified.

Study	Random sequence generation (selection bias)	Allocation concealment (selection bias)	Blinding of participants, personnel, and outcome assessors (performance bias and detection bias)	Incomplete outcome data (attrition bias)—all outcomes	Selective reporting (reporting bias)	Other bias
			analysis at the end of the trial."			
Korczyn, 1999 [15]	**Judgement:** Unclear risk.<br><br>**Support:** Not reported.	**Judgement:** Unclear risk.<br><br>**Support:** Not reported.	**Judgement:** Unclear risk.<br><br>**Support:** Quote: "Ropinirole and bromocriptine were supplied in tablet and capsule form, respectively, and a double-dummy technique was used to maintain study blinding."<br><br>Blinding was not explicitly described for personnel or investigators.	**Judgement:** Unclear risk.<br><br>**Support:** Intention to treat analysis.<br><br>Similar but high proportions in study arms did not complete treatment (40% ropinirole group and 33% in the bromocriptine).	**Judgement:** Unclear risk.<br><br>**Support:** Unclear if protocol publicly available.	**Judgement:** Low risk.<br><br>**Support:** None identified.
LeWitt, 2007 [16]	**Judgement:** Low risk.<br><br>**Support:**	**Judgement:** Low risk.<br><br>**Support:**	**Judgement:** Low risk.<br><br>**Support:**	**Judgement:** Low risk.<br><br>**Support:**	**Judgement:** Unclear risk.<br><br>**Support:**	**Judgement:** Low risk.<br><br>**Support:** None identified.

Study	Random sequence generation (selection bias)	Allocation concealment (selection bias)	Blinding of participants, personnel, and outcome assessors (performance bias and detection bias)	Incomplete outcome data (attrition bias)—all outcomes	Selective reporting (reporting bias)	Other bias
	Quote: "Treatment assignments were randomly allocated in equal groups and stratified by clinical sites. The interactive voice response telephone system was used for randomization."	Quote: "The IVRS system automatically assigned a randomization number to each subject and this number was used to identify study medication for the subject throughout the trial keeping all study personnel blinded to the treatment assignment."	Patients and all study personnel blinded to treatment assignment.	Intention to treat analysis.<br><br>Similar proportions of participants in study arms completed the study.	Protocol not publicly available.	
Lieberman, 1997 [17]	**Judgement:** Low risk.<br><br>**Support:** Quote: "The randomization schedule was computer-generated using a block size of four."	**Judgement:** Unclear risk.<br><br>**Support:** Not reported.	**Judgement:** Low risk.<br><br>**Support:** Quote: "All members of the monitoring team involved in the day-to- day supervision	**Judgement:** Low risk.<br><br>**Support:** Similar proportions of participants in study arms completed the study.	**Judgement:** Unclear risk.<br><br>**Support:** Not reported.	**Judgement:** Low risk.<br><br>**Support:** None identified.

Study	Random sequence generation (selection bias)	Allocation concealment (selection bias)	Blinding of participants, personnel, and outcome assessors (performance bias and detection bias)	Incomplete outcome data (attrition bias)—all outcomes	Selective reporting (reporting bias)	Other bias
			and/or monitoring of the study were blinded to the treatment assignments."			
Mizuno, 2012 [18]	**Judgement:** Low risk.<br><br>**Support:** Quote: "The randomization list was generated by the sponsor."	**Judgement:** Low risk.<br><br>**Support:** Contents of the randomization list were not known by study site personnel.	**Judgement:** Low risk.<br><br>**Support:** Quote: "The randomization list was generated by the sponsor, and its contents were not known by trial site personnel."<br><br>Pramipexole and placebo were matching.	**Judgement:** Low risk.<br><br>**Support:** Intention to treat analysis.<br><br>Similar proportions of patients in treatment groups did not complete the study.	**Judgement:** Low risk.<br><br>**Support:** Quote: "This trial was conducted in compliance with the protocol and principles of the Declaration of Helsinki (1996 version) and in accordance with the International Conference on Harmonisation Harmonised Tripartite Guideline for Good Clinical Practice (GCP) and the Japanese	**Judgement:** Low risk.<br><br>**Support:** None identified.

Study	Random sequence generation (selection bias)	Allocation concealment (selection bias)	Blinding of participants, personnel, and outcome assessors (performance bias and detection bias)	Incomplete outcome data (attrition bias)—all outcomes	Selective reporting (reporting bias)	Other bias
					GCP regulations (Ministry of Health and Welfare Ordinance No. 28, March 27, 1997). The clinical trial identifier number is NCT00560508."	
Mizuno, 2014 [19]	**Judgement:** Low risk.<br><br>**Support:** Quote: "Eligible patients were randomized 2:2:1 to receive rotigotine, ropinirole, or placebo using a dynamic allocation procedure."	**Judgement:** Unclear risk.<br><br>**Support:** Not reported.	**Judgement:** Unclear risk.<br><br>**Support:** Quote: "A double-dummy technique was used to maintain blinding with placebo patches or tablets. Technique used to blind investigators and personnel was not described.	**Judgement:** Low risk.<br><br>**Support:** Similar proportions of patients receiving placebo (20%) compared to ropinirole (13-15%) discontinued treatment.	**Judgement:** Low risk.<br><br>**Support:** Study protocol published and available.	**Judgement:** Low risk.<br><br>**Support:** None identified.
Moller, 2005 [21]	**Judgement:** Unclear risk.<br><br>**Support:**	**Judgement:** Unclear risk.<br><br>**Support:**	**Judgement:** Unclear risk.<br><br>**Support:**	**Judgement:** High risk.<br><br>**Support:**	**Judgement:** Unclear risk.<br><br>**Support:**	**Judgement:** Low risk.<br><br>**Support:**

Study	Random sequence generation (selection bias)	Allocation concealment (selection bias)	Blinding of participants, personnel, and outcome assessors (performance bias and detection bias)	Incomplete outcome data (attrition bias)—all outcomes	Selective reporting (reporting bias)	Other bias
	Not reported.	Not reported.	Not reported.	A significantly higher proportion of discontinued placebo (60%) compared to treatment group (26%).	Unable to locate study protocol.	None identified.
Navan, 2003 [22]	**Judgement:** Low risk.<br><br>**Support:** Quote: " ...patients were randomly assigned in blocks of three by using a computer".	**Judgement:** High risk.<br><br>**Support:** Quote: "The randomisation was performed by the research pharmacist... who also administered the medications so that patients and assessors were blind to treatment allocation".	**Judgement:** High risk.<br><br>**Support:** Patient blinding was likely ineffective, as patients correctly ascertained whether or not they received an active treatment (pramipexole or pergolide) or placebo.	**Judgement:** High risk.<br><br>**Support:** Dropping out was associated with receiving pergolide.	**Judgement:** Unclear risk.<br><br>**Support:** Not reported.	**Judgement:** Low risk.<br><br>**Support:** None identified.
Nicholas, 2014 [23]	**Judgement:** Low risk.<br><br>**Support:**	**Judgement:** Low risk.<br><br>**Support:**	**Judgement:** Low risk.<br><br>**Support:**	**Judgement:** Low risk.<br><br>**Support:**	**Judgement:** Low risk.<br><br>**Support:**	**Judgement:** Unclear risk.<br><br>**Support:** None identified.

Study	Random sequence generation (selection bias)	Allocation concealment (selection bias)	Blinding of participants, personnel, and outcome assessors (performance bias and detection bias)	Incomplete outcome data (attrition bias)—all outcomes	Selective reporting (reporting bias)	Other bias
	Quote: "…eligible patients were randomized by computer".	Quote: "Study investigators telephoned an interactive voice response system to allocate patients, based on a randomization schedule produced by UCB Pharma."	Investigators and patients were blinded.	Similar proportions of patients randomized to each study group discontinued treatment.	Publicly available study protocol.	
Pahwa, 2007 [24]	**Judgement:** Low risk.<br><br>**Support:** Quote: "A computer generated randomization schedule using the Registration and Medication Ordering System was used."	**Judgement:** Low risk.<br><br>**Support:** Investigators phoned into the Registration and Medication Ordering System to register and randomize patients.	**Judgement:** Unclear risk.<br><br>**Support:** Investigators and patients were blinded to treatment allocation. Placebo tablets were identical in appearance and packaging to the active treatment. Investigators could only	**Judgement:** Low risk.<br><br>**Support:** 17% of patients in treatment group and 30% of placebo group discontinued treatment. The number of patients reporting adverse events leading to withdrawal was low and similar in	**Judgement:** Unclear risk.<br><br>**Support:** Not reported.	**Judgement:** Low risk.<br><br>**Support:** None identified.

Study	Random sequence generation (selection bias)	Allocation concealment (selection bias)	Blinding of participants, personnel, and outcome assessors (performance bias and detection bias)	Incomplete outcome data (attrition bias)—all outcomes	Selective reporting (reporting bias)	Other bias
			unmask the blinding in emergencies.	both treatment groups.		
Poewe, 2007 [26]	**Judgement:** Low risk.<br><br>**Support:** Quote: "Randomisation was implemented by an interactive voice response system with a computerised randomisation schedule stratified by centre in blocks of five".	**Judgement:** Low risk.<br><br>**Support:** Patients were allocated to study groups using an interactive voice response system.	**Judgement:** Low risk.<br><br>**Support:** Investigators were blinded to all patient treatment details, which were allocated and maintained by the interactive voice response system. Study participants were unaware of the allocated treatment.	**Judgement:** Unclear risk.<br><br>**Support:** 11-26% of patients discontinued treatment from the study arms.<br><br>Similar proportion of patients withdrew from the study due to adverse events in each study arm.	**Judgement:** Unclear risk.<br><br>**Support:** Not reported.	**Judgement:** Low risk.<br><br>**Support:** None identified.
Rascol, 1996 [27]	**Judgement:** Unclear risk.<br><br>**Support:** Not reported.	**Judgement:** Unclear risk.<br><br>**Support:** Not reported.	**Judgement:** Unclear risk.<br><br>**Support:** Not reported.	**Judgement:** Unclear risk.<br><br>**Support:** A higher proportion of patients in the placebo group	**Judgement:** Unclear risk.<br><br>**Support:** Not reported.	**Judgement:** Low risk.<br><br>**Support:** None identified.

Study	Random sequence generation (selection bias)	Allocation concealment (selection bias)	Blinding of participants, personnel, and outcome assessors (performance bias and detection bias)	Incomplete outcome data (attrition bias)—all outcomes	Selective reporting (reporting bias)	Other bias
				withdrew from the study compared to the treatment group.		
Rascol, 1998 [28]	**Judgement:** Unclear risk.<br><br>**Support:** Not reported.	**Judgement:** High risk.<br><br>**Support:** Ropinirole was supplied as tablets and levodopa as capsules.	**Judgement:** Low risk.<br><br>**Support:** Double blind.<br><br>Investigators and all other site and monitoring staff remained masked after the interim analysis, with the authors (investigators) having access to tables and figures only.	**Judgement:** Low risk.<br><br>**Support:** All participants were examined for this outcome.	**Judgement:** Unclear risk.<br><br>**Support:** Not reported.	**Judgement:** Low risk.<br><br>**Support:** None identified.
Rascol, 2000 [29]	**Judgement:** Unclear risk.<br><br>**Support:** Quote: "Sealed copies of the	**Judgement:** Low risk.<br><br>**Support:** Quote: "Sealed copies of the	**Judgement:** Low risk.<br><br>**Support:** Blinding was maintained using	**Judgement:** Low risk.<br><br>**Support:** Intention to treat analysis.	**Judgement:** Unclear risk.<br><br>**Support:** Protocol unavailable.	**Judgement:** Low risk.<br><br>**Support:** None identified.

Study	Random sequence generation (selection bias)	Allocation concealment (selection bias)	Blinding of participants, personnel, and outcome assessors (performance bias and detection bias)	Incomplete outcome data (attrition bias)—all outcomes	Selective reporting (reporting bias)	Other bias
	randomization code were held by the principal investigator at each site and by the study sponsor."<br><br>Double-dummy technique used.<br><br>Method for generating randomization code not described.	randomization code were held by the principal investigator at each site and by the study sponsor."<br><br>Double-dummy technique used.	a double-dummy technique. Sealed copies of the randomization code were held by the principal investigator at each site and by the study sponsor.<br><br>Double-dummy technique used.	Similar proportion of patients did not complete the study.		
Rascol, 2006 [30]	**Judgement:** Low risk.<br><br>**Support:** Quote: "Randomization procedures were performed via interactive voice response system."	**Judgement:** Low risk.<br><br>**Support:** Randomization was performed using an interactive voice response system. Double-dummy technique used.	**Judgement:** Low risk.<br><br>**Support:** Double-blind conditions were maintained from randomization up to the end of the 2-year study.	**Judgement:** Low risk.<br><br>**Support:** 12% of placebo group and 20% of treatment group did not complete the study.	**Judgement:** Unclear risk.<br><br>**Support:** Not reported.	**Judgement:** Low risk.<br><br>**Support:** None identified.

Study	Random sequence generation (selection bias)	Allocation concealment (selection bias)	Blinding of participants, personnel, and outcome assessors (performance bias and detection bias)	Incomplete outcome data (attrition bias)—all outcomes	Selective reporting (reporting bias)	Other bias
Sampaio, 2011 [32]	**Judgement:** Low risk.<br><br>**Support:** Quote: "Enrolled patients were allocated a randomization number using a central Interactive Voice Recognition System that corresponded to the trial kit number for study medication."	**Judgement:** Low risk.<br><br>**Support:** Quote: "Enrolled patients were allocated a randomization number using a central Interactive Voice Recognition System that corresponded to the trial kit number for study medication."	**Judgement:** Low risk.<br><br>**Support:** Investigators and patients were blinded to treatment allocation. Identical capsules for study drug and placebo.	**Judgement:** Low risk.<br><br>**Support:** A greater proportion of patients in the pardoprunox group did not complete the study relative to the pramipexole and placebo groups.	**Judgement:** Low risk.<br><br>**Support:** Trial was registered.	**Judgement:** Low risk.<br><br>**Support:** None identified.
Schapira, 2011 [33]	**Judgement:** Low risk.<br><br>**Support:** Quote: "Treatment allocation was determined by randomization code provided by the study sponsor, using the	**Judgement:** Low risk.<br><br>**Support:** Quote: "Access to the randomization schedule was restricted to the sponsor's Clinical Trial Support and Clinical Trial	**Judgement:** Low risk.<br><br>**Support:** Quote: "Access to the randomization schedule was restricted to the sponsor's Clinical Trial Support and Clinical Trial	**Judgement:** Unclear risk.<br><br>**Support:** 6-10% of patients did not complete the interventions across the study arms.	**Judgement:** Unclear risk.<br><br>**Support:** Not reported.	**Judgement:** Low risk.<br><br>**Support:** None identified.

Study	Random sequence generation (selection bias)	Allocation concealment (selection bias)	Blinding of participants, personnel, and outcome assessors (performance bias and detection bias)	Incomplete outcome data (attrition bias)—all outcomes	Selective reporting (reporting bias)	Other bias
	commercial program PMX CTM."	Supplies Unit, with no access by any persons directly involved in the study's conduct or data analysis."	Supplies Unit, with no access by any persons directly involved in the study's conduct or data analysis."<br><br>Double blinding was maintained by having all patients receive four treatments per day.			
Schapira, 2013 [34]	**Judgement:** Low risk.<br><br>**Support:** Quote: "Patients were randomly assigned (1:1 ratio) by centralised, computerised, sponsor-maintained randomisation schedule to	**Judgement:** Low risk.<br><br>**Support:** Allocation to treatment arm was performed using a centralised sponsor-maintained randomization schedule.	**Judgement:** Low risk.<br><br>**Support:** Quote: "All patients and investigators were masked to study treatment... Masking was maintained during period 2 for all but two patients	**Judgement:** Unclear risk.<br><br>**Support:** Similar proportion of patients in study arms completed the study.<br><br>The delayed-start design minimized patient	**Judgement:** Low risk.<br><br>**Support:** Quote: "The study was conducted in accordance with its protocol, with good clinical practice, and with the provisions of the Declaration of	**Judgement:** Low risk.<br><br>**Support:** None identified.

Study	Random sequence generation (selection bias)	Allocation concealment (selection bias)	Blinding of participants, personnel, and outcome assessors (performance bias and detection bias)	Incomplete outcome data (attrition bias)—all outcomes	Selective reporting (reporting bias)	Other bias
	receive double-blind pramipexole or placebo."		unmasked for non-emergencies. Masking was maintained during period 2 for all but two patients unmasked for non-emergencies. An independent masked rater distinct from the study investigators assessed patients at baseline and 15 months, and was separate from the masked investigator who assessed patients at every visit."	withdrawal and missing data.	Helsinki and its amendments."	
Seiple, 2016 [35]	**Judgement:** Unclear risk.<br><br>**Support:** Not reported.	**Judgement:** Unclear risk.<br><br>**Support:** Not reported.	**Judgement:** High risk.<br><br>**Support:** Open-label study.	**Judgement:** Low risk.<br><br>**Support:** Intention to treat analysis.	**Judgement:** Unclear risk.<br><br>**Support:** Not reported.	**Judgement:** Low risk.<br><br>**Support:** None identified.

Study	Random sequence generation (selection bias)	Allocation concealment (selection bias)	Blinding of participants, personnel, and outcome assessors (performance bias and detection bias)	Incomplete outcome data (attrition bias)—all outcomes	Selective reporting (reporting bias)	Other bias
			Quote: "Ophthalmologists, central reading centres, the Expert Panel, and sponsor's in-house team remained masked to treatment allocation, although site investigators and subjects were aware."	Similar proportions of patients in each study arm completed the study.		
Stocchi, 2011 [36]	**Judgement:** Low risk.<br><br>**Support:** Patients were randomized to treatment groups using an interactive voice recognition system.	**Judgement:** Unclear risk.<br><br>**Support:** Not reported.	**Judgement:** Unclear risk.<br><br>**Support:** Not reported.	**Judgement:** Low risk.<br><br>**Support:** Follow-up data were available for all participants.<br><br>The proportion of patients that withdrew from the study was	**Judgement:** Low risk.<br><br>**Support:** Protocol is available at ClinicalTrials.gov.	**Judgement:** Low risk.<br><br>**Support:** None identified.

Study	Random sequence generation (selection bias)	Allocation concealment (selection bias)	Blinding of participants, personnel, and outcome assessors (performance bias and detection bias)	Incomplete outcome data (attrition bias)—all outcomes	Selective reporting (reporting bias)	Other bias
				similar in the treatment groups.		
Viallet, 2013 [38]	**Judgement:** Unclear risk.<br><br>**Support:** Not reported.	**Judgement:** Unclear risk.<br><br>**Support:** Not reported.	**Judgement:** Unclear risk.<br><br>**Support:** Not reported.	**Judgement:** Unclear risk.<br><br>**Support:** Higher number of patients discontinued the study due to adverse event in the pramipexole group (8/56) than in the rasagiline group (3/53).	**Judgement:** Unclear risk.<br><br>**Support:** Not reported.	**Judgement:** Low risk.<br><br>**Support:** None identified.
Wang, 2014 [39]	**Judgement:** Low risk.<br><br>**Support:** Quote: "Randomization was conducted by a validated system using a pseudo-random number generator".	**Judgement:** Low risk.<br><br>**Support:** Treatment assignment was not predictable.	**Judgement:** Low risk.<br><br>**Support:** Quote: "Throughout the study, the persons who administered the medications, the raters, and the patients were all blind to	**Judgement:** Low risk.<br><br>**Support:** Outcome data are missing for only 2 participants.<br><br>Few adverse events led to treatment discontinuation in	**Judgement:** Unclear risk.<br><br>**Support:** Not reported.	**Judgement:** Low risk.<br><br>**Support:** None identified.

Study	Random sequence generation (selection bias)	Allocation concealment (selection bias)	Blinding of participants, personnel, and outcome assessors (performance bias and detection bias)	Incomplete outcome data (attrition bias)—all outcomes	Selective reporting (reporting bias)	Other bias
			medication assignments."	both treatment groups (ER: 4.7%; IR: 5.0%).		
Watts, 2007 [40]	**Judgement:** Unclear risk.<br><br>**Support:** Not reported.	**Judgement:** Unclear risk.<br><br>**Support:** Not reported.	**Judgement:** Unclear risk.<br><br>**Support:** Placebo patches were identical in appearance. Method of blinding investigators and study personnel was not described.	**Judgement:** Low risk.<br><br>**Support:** Similar proportions of patients in both study arms completed the study.	**Judgement:** Unclear risk.<br><br>**Support:** Unclear if protocol is available.	**Judgement:** Low risk.<br><br>**Support:** None identified.
Wermuth, 1998 [41]	**Judgement:** Unclear risk.<br><br>**Support:** Not reported.	**Judgement:** Low risk.<br><br>**Support:** Not reported.	**Judgement:** Unclear risk.<br><br>**Support:** Placebo tablets matching the pramipexole tablets. Methods for blinding investigators and personnel were not described.	**Judgement:** Low risk.<br><br>**Support:** All participants were included in analysis of safety outcomes.	**Judgement:** Unclear risk.<br><br>**Support:** Protocol not available.	**Judgement:** Low risk.<br><br>**Support:** None identified.

Study	Random sequence generation (selection bias)	Allocation concealment (selection bias)	Blinding of participants, personnel, and outcome assessors (performance bias and detection bias)	Incomplete outcome data (attrition bias)—all outcomes	Selective reporting (reporting bias)	Other bias
Zesiewicz, 2017 [44]	**Judgement:** Low risk.<br><br>**Support:** Eligible subjects were randomized using an interactive voice recognition system.	**Judgement:** Unclear risk.<br><br>**Support:** Not reported.	**Judgement:** Unclear risk.<br><br>**Support:** Ropinirole PR and placebo tablets were identical in appearance. Blinding of investigators and personnel was not described.	**Judgement:** Unclear risk.<br><br>**Support:** Intent to treat analysis.<br><br>0-25% of patients in each study group did not complete the study.	**Judgement:** Unclear risk.<br><br>**Support:** Not reported.	**Judgement:** Low risk.<br><br>**Support:** None identified.
Zhang, 2013 [45]	**Judgement:** Unclear risk.<br><br>**Support:** Not reported.	**Judgement:** Unclear risk.<br><br>**Support:** Not reported.	**Judgement:** Unclear risk.<br><br>**Support:** Quote: "To achieve blinding, active ropinirole PR and placebo tablets were identical in appearance and all packaging maintained the double-blind nature of the study." Blinding of	**Judgement:** Low risk.<br><br>**Support:** 80% and 93% of patients in the placebo and ropinirole group completed the study, respectively.	**Judgement:** Low risk.<br><br>**Support:** Protocol available.	**Judgement:** Low risk.<br><br>**Support:** None identified.

Study	Random sequence generation (selection bias)	Allocation concealment (selection bias)	Blinding of participants, personnel, and outcome assessors (performance bias and detection bias)	Incomplete outcome data (attrition bias)—all outcomes	Selective reporting (reporting bias)	Other bias
			investigators and personnel was not described.			
Ziegler, 2003 [46]	**Judgement:** Unclear risk.<br><br>**Support:** Not reported.	**Judgement:** Unclear risk.<br><br>**Support:** Not reported.	**Judgement:** Unclear risk.<br><br>**Support:** Study drug and placebo were identical. Blinding of personnel and investigators was not described.	**Judgement:** Low risk.<br><br>**Support:** Similar proportions of patients in each study group withdrew from the study.	**Judgement:** Unclear risk.<br><br>**Support:** Not reported.	**Judgement:** Low risk.<br><br>**Support:** None identified.

Supplementary Material S9. Summary of Findings (SOF) tables.

Findings from GRADE assessments are presented in the following Summary of Findings tables generated by GRADEpro GDT (https://www.gradepro.org) software for each comparison included in our review.

GRADE working group grades of evidence

- **High certainty:** We are very confident that the true effect lies close to that of the estimate of the effect;
- **Moderate certainty:** We are moderately confident in the effect estimate: the true effect is likely to be close to the estimate of the effect, but there is a possibility that it is substantially different;
- **Low certainty:** Our confidence in the effect estimate is limited: the true effect may be substantially different from the estimate of the effect;
- **Very low certainty:** We have very little confidence in the effect estimate: the true effect is likely to be substantially different from the estimate of effect.

Table S9.1. Pramipexole compared to placebo for patients with Parkinson's disease

Patient or population: patients with Parkinson's disease
Intervention: pramipexole
Comparison: placebo

Outcomes	**Anticipated absolute effects*** (95% CI)		Relative effect (95% CI)	№ of participants (studies)	Certainty of the evidence (GRADE)	Comments
	**Risk with placebo**	**Risk with pramipexole**				
Peripheral Edema	22 per 1,000	**62 per 1,000** (32 to 115)	**OR 2.97** (1.50 to 5.88)	1072 (3 RCTs)	⊕⊕⊕◯ Moderate[a]	
Orthostatic Hypotension (Symptomatic and Asymptomatic)	75 per 1,000	**90 per 1,000** (57 to 138)	**OR 1.22** (0.75 to 1.98)	1183 (5 RCTs)	⊕⊕◯◯ Low[b,c,d]	
Symptomatic Orthostatic Hypotension	73 per 1,000	**110 per 1,000** (64 to 183)	**OR 1.57** (0.87 to 2.84)	680 (3 RCTs)	⊕⊕⊕◯ Moderate[c,d]	

Table S9.1. Pramipexole compared to placebo for patients with Parkinson's disease

Patient or population: patients with Parkinson's disease
Intervention: pramipexole
Comparison: placebo

Outcomes	Anticipated absolute effects* (95% CI)		Relative effect (95% CI)	№ of participants (studies)	Certainty of the evidence (GRADE)	Comments
	**Risk with placebo**	**Risk with pramipexole**				
Asymptomatic Orthostatic Hypotension	340 per 1,000	**357 per 1,000** (286 to 432)	**OR 1.08** (0.78 to 1.48)	723 (2 RCTs)	⊕⊕◯◯ Low[c,d,e]	
Hypertension (Asymptomatic Orthostatic Hypertension)	1,000 per 1,000	**0 per 1,000** (0 to 0)	not estimable	55 (1 RCT)	⊕◯◯◯ Very low[f,g]	
Hypertension (Symptomatic Orthostatic Hypertension)	185 per 1,000	**250 per 1,000** (83 to 549)	**OR 1.47** (0.40 to 5.35)	55 (1 RCT)	⊕◯◯◯ Very low[c,d,f]	
Hypertension	30 per 1,000	**9 per 1,000** (0 to 191)	**OR 0.30** (0.01 to 7.54)	69 (1 RCT)	⊕⊕◯◯ Low[c,d,h]	
Valvulopathy	**Low**		**OR 1.13** (0.88 to 1.45)	1000 cases, 3044 controls (2 non-randomized studies)	⊕◯◯◯ Very low[i]	
	30 per 1,000	**34 per 1,000** (27 to 43)				
Heart Failure	**Low**		**OR 1.46** (1.03 to 2.08)	3101 cases, 14408 controls (2 non-randomized studies)	⊕⊕◯◯ Low	
	47 per 1,000	**66 per 1,000** (48 to 92)				

Table S9.1. Pramipexole compared to placebo for patients with Parkinson's disease

Patient or population: patients with Parkinson's disease
Intervention: pramipexole
Comparison: placebo

Outcomes	Anticipated absolute effects* (95% CI)		Relative effect (95% CI)	№ of participants (studies)	Certainty of the evidence (GRADE)	Comments
	**Risk with placebo**	**Risk with pramipexole**				
Hypotension	**Low**		**OR 1.12** (0.80 to 1.44)	869 cases, 2607 controls (1 non-randomized study)	⊕◯◯◯ Very low[i]	
	30 per 1,000	**33 per 1,000** (24 to 43)				
Myocardial Infarction	**Low**		**OR 0.68** (0.46 to 1.01)	482 cases, 1446 controls (1 non-randomized study)	⊕◯◯◯ Very low[i]	
	30 per 1,000	**21 per 1,000** (14 to 30)				
Stroke	**Low**		**OR 1.09** (0.72 to 1.66)	478 cases, 1434 controls (1 non-randomized study)	⊕◯◯◯ Very low[i]	
	30 per 1,000	**33 per 1,000** (22 to 49)				

***The risk in the intervention group** (and its 95% confidence interval) is based on the assumed risk in the comparison group and the **relative effect** of the intervention (and its 95% CI).
CI: confidence interval; **OR:** odds ratio.

Explanations

a. Unclear as to whether blinding of participants, personnel, and outcome assessors occurred in two [14,32] of the three studies;
b. The study [8] is likely subject to considerable attrition bias and is heavily weighted in the pooled analysis;
c. A 95% CI contains the potential for appreciable harm, as well as benefit;
d. Few events reported;
e. The study [21] is likely subject to considerable attrition bias and is moderately weighted in the pooled analysis;

f. This study [10] provides limited or no information about sequence generation; allocation concealment; and blinding of participants, personnel, and outcome assessors. Selective outcome reporting may also have occurred;
g. No events reported; unable to determine the extent of benefit or harm;
h. This study [41] provides limited or no information about sequence generation and blinding of participants, personnel, and outcome assessors. Selective outcome reporting may also have occurred.;
i. A 95% CI contains the potential for some harm, as well as some benefit.

Table S9.2. Pramipexole compared to non-ergot dopamine agonists for patients with Parkinson's disease

Patient or population: patients with Parkinson's disease
Intervention: pramipexole
Comparison: non-ergot dopamine agonists

Outcomes	**Anticipated absolute effects*** (95% CI)		Relative effect (95% CI)	№ of participants (studies)	Certainty of the evidence (GRADE)	Comments
	**Risk with non-ergot dopamine agonists**	**Risk with pramipexole**				
Heart Failure	**Low**		**RR 1.53** (0.92 to 2.57)	72 cases, 604 controls (1 non-randomized study)	⊕◯◯◯ Very low[a]	
	84 per 1,000	**128 per 1,000** (77 to 216)				

***The risk in the intervention group** (and its 95% confidence interval) is based on the assumed risk in the comparison group and the **relative effect** of the intervention (and its 95% CI).
CI: confidence interval; **RR:** risk ratio.

Explanations
a. A 95% CI contains the potential for appreciable harm, as well as some benefit.

Table S9.3. Pramipexole immediate release (IR) compared to pramipexole extended release (ER) for patients with Parkinson's disease

Patient or population: patients with Parkinson's disease
Intervention: pramipexole immediate release (IR)
Comparison: pramipexole extended release (ER)

Outcomes	**Anticipated absolute effects*** (95% CI)		Relative effect (95% CI)	№ of participants (studies)	Certainty of the evidence (GRADE)	Comments
	**Risk with pramipexole extended release (ER)**	**Risk with pramipexole immediate release (IR)**				
Orthostatic Hypotension (Symptomatic and Asymptomatic)	24 per 1,000	**19 per 1,000** (8 to 46)	**OR 0.79** (0.32 to 1.94)	924 (3 RCTs)	⊕⊕⊕◯ Moderate[a,b]	
Hypotension	4 per 1,000	**21 per 1,000** (2 to 156)	**OR 4.98** (0.58 to 42.94)	473 (1 RCT)	⊕⊕⊕◯ Moderate[b,c]	

***The risk in the intervention group** (and its 95% confidence interval) is based on the assumed risk in the comparison group and the **relative effect** of the intervention (and its 95% CI).
CI: confidence interval; **OR:** odds ratio.

Explanations
a. A 95% CI contains the potential for possible harm and benefit;
b. Few events reported;
c. A 95% CI contains the potential for appreciable harm, as well as possible benefit.

Table S9.4. Pramipexole extended release (ER) compared to placebo for patients with Parkinson's disease

Patient or population: patients with Parkinson's disease
Intervention: pramipexole extended release (ER)
Comparison: placebo

Outcomes	**Anticipated absolute effects*** (95% CI)		Relative effect (95% CI)	№ of participants (studies)	Certainty of the evidence (GRADE)	Comments
	**Risk with placebo**	**Risk with pramipexole extended release (ER)**				
Orthostatic Hypotension (Symptomatic and Asymptomatic)	11 per 1,000	**18 per 1,000** (3 to 101)	**OR 1.64** (0.27 to 9.94)	342 (1 RCT)	⊕⊕◯◯ Low[a,b,c]	

***The risk in the intervention group** (and its 95% confidence interval) is based on the assumed risk in the comparison group and the **relative effect** of the intervention (and its 95% CI).
CI: confidence interval; **OR:** odds ratio.

Explanations
a. It is unclear if there is selective outcome reporting in this study [33];
b. A 95% CI contains the potential for appreciable harm, as well as possible benefit;
c. Few events reported.

Table S9.5. Pramipexole compared to bromocriptine for patients with Parkinson's disease

Patient or population: patients with Parkinson's disease
Intervention: pramipexole
Comparison: bromocriptine

Outcomes	Anticipated absolute effects* (95% CI)		Relative effect (95% CI)	№ of participants (studies)	Certainty of the evidence (GRADE)	Comments
	**Risk with bromocriptine**	**Risk with pramipexole**				
Orthostatic Hypotension (Symptomatic and Asymptomatic)	440 per 1,000	**401 per 1,000** (266 to 554)	**OR 0.85** (0.46 to 1.58)	164 (1 RCT)	⊕◯◯◯ Very low[a,b,c]	

***The risk in the intervention group** (and its 95% confidence interval) is based on the assumed risk in the comparison group and the **relative effect** of the intervention (and its 95% CI).
CI: confidence interval; **OR:** odds ratio.

Explanations
a. While randomized, this study [8] did not report on sequence generation and allocation concealment, and it is unclear as to whether participants, personnel, and outcomes assessors were blinded to exposure assignment. Additionally, the study may be subject to high attrition bias;
b. A 95% CI contains potential of appreciable benefit and harm;
c. Small sample with few events reported.

Table S9.6. Pramipexole compared to levodopa for patients with Parkinson's disease

Patient or population: patients with Parkinson's disease
Intervention: pramipexole
Comparison: levodopa

Outcomes	**Anticipated absolute effects*** (95% CI)		Relative effect (95% CI)	№ of participants (studies)	Certainty of the evidence (GRADE)	Comments
	**Risk with levodopa**	**Risk with pramipexole**				
Peripheral Edema	13 per 1,000	**46 per 1,000** (10 to 192)	**OR 3.60** (0.73 to 17.61)	301 (1 RCT)	⊕⊕◯◯ Low[a,b,c]	
Hypotension	**Low**		**OR 1.09** (0.61 to 1.96)	539 cases, 1665 controls (1 non-randomized study)	⊕◯◯◯ Very low[d]	
	31 per 1,000	**34 per 1,000** (19 to 59)				
Myocardial Infarction	**Low**		**OR 0.88** (0.44 to 1.77)	324 cases, 949 controls (1 non-randomized study)	⊕◯◯◯ Very low[d]	
	29 per 1,000	**26 per 1,000** (13 to 51)				
Stroke	**Low**		**OR 1.08** (0.50 to 2.30)	343 cases, 929 controls (1 non-randomized study)	⊕◯◯◯ Very low[d]	
	29 per 1,000	**32 per 1,000** (15 to 65)				
Valvulopathy	**Low**		**OR 1.09** (0.68 to 1.75)	659 cases, 1986 controls (1 non-randomized study)	⊕◯◯◯ Very low[d]	
	30 per 1,000	**33 per 1,000** (21 to 52)				
Heart Failure	**Low**		**OR 1.54** (1.21 to 1.98)	1979 cases, 29143 controls (2 non-randomized studies)	⊕⊕◯◯ Low	
	147 per 1,000	**210 per 1,000** (173 to 255)				

Table S9.6. Pramipexole compared to levodopa for patients with Parkinson's disease

Patient or population: patients with Parkinson's disease
Intervention: pramipexole
Comparison: levodopa

Outcomes	**Anticipated absolute effects*** (95% CI)		Relative effect (95% CI)	№ of participants (studies)	Certainty of the evidence (GRADE)	Comments
	**Risk with levodopa**	**Risk with pramipexole**				

***The risk in the intervention group** (and its 95% confidence interval) is based on the assumed risk in the comparison group and the **relative effect** of the intervention (and its 95% CI).
CI: confidence interval; **OR:** odds ratio.

Explanations

a. This study [9] may be subject to attrition bias;
b. A 95% CI includes the potential for appreciable harm, as well as possible benefit;
c. Small sample with few events;
d. A 95% CI includes the potential for possible harm, as well as possible benefit.

Table S9.7. Pramipexole compared to pardoprunox for patients with Parkinson's disease

Patient or population: patients with Parkinson's disease
Intervention: pramipexole
Comparison: pardoprunox

Outcomes	**Anticipated absolute effects*** (95% CI)		Relative effect (95% CI)	№ of participants (studies)	Certainty of the evidence (GRADE)	Comments
	**Risk with pardoprunox**	**Risk with pramipexole**				
Peripheral Edema	28 per 1,000	**112 per 1,000** (34 to 313)	**OR 4.42** (1.22 to 15.96)	224 (1 RCT)	⨁⨁⨁◯ Moderate[a,b]	

*__The risk in the intervention group__ (and its 95% confidence interval) is based on the assumed risk in the comparison group and the **relative effect** of the intervention (and its 95% CI).
CI: confidence interval; **OR:** odds ratio.

Explanations
a. A 95% CI contains potential for negligible to appreciable harm;
b. Small sample with few events.

Table S9.8. Pramipexole compared to pergolide for patients with Parkinson's disease

Patient or population: patients with Parkinson's disease
Intervention: pramipexole
Comparison: pergolide

Outcomes	**Anticipated absolute effects*** (95% CI)		Relative effect (95% CI)	№ of participants (studies)	Certainty of the evidence (GRADE)	Comments
	**Risk with pergolide**	**Risk with pramipexole**				
Hypotension (Symptomatic Orthostatic)	0 per 1,000	**0 per 1,000** (0 to 0)	not estimable	20 (1 RCT)	⊕⊕◯◯ Low[a,b]	

*__The risk in the intervention group__ (and its 95% confidence interval) is based on the assumed risk in the comparison group and the **relative effect** of the intervention (and its 95% CI).
CI: confidence interval.

Explanations
a. This study may be biased as a result of poor allocation concealment and inadequate blinding of personnel to exposure assignment. Selective outcome reporting may also be possible;
b. Small sample with no events to determine difference between the interventions.

Table S9.9. Pramipexole compared to ergot dopamine agonists for patients with Parkinson's disease

Patient or population: patients with Parkinson's disease
Intervention: pramipexole
Comparison: ergot dopamine agonists

Outcomes	**Anticipated absolute effects*** (95% CI)		Relative effect (95% CI)	№ of participants (studies)	Certainty of the evidence (GRADE)	Comments
	**Risk with ergot dopamine agonists**	**Risk with pramipexole**				
Heart Failure	**Low**		**RR 1.07** (0.66 to 1.74)	110 cases, 752 controls (1 non-randomized study)	⊕◯◯◯ Very low[a]	
	68 per 1,000	**73 per 1,000** (45 to 119)				

*__The risk in the intervention group__ (and its 95% confidence interval) is based on the assumed risk in the comparison group and the **relative effect** of the intervention (and its 95% CI).
CI: confidence interval; **RR:** risk ratio.

Explanations
a. A 95% CI contains the potential for some harm, as well as some benefit.

Table S9.10. Pramipexole compared to ropinirole for patients with Parkinson's disease

Patient or population: patients with Parkinson's disease
Intervention: pramipexole
Comparison: ropinirole

Outcomes	**Anticipated absolute effects*** (95% CI)		Relative effect (95% CI)	№ of participants (studies)	Certainty of the evidence (GRADE)	Comments
	**Risk with ropinirole**	**Risk with pramipexole**				
Orthostatic Hypotension (Symptomatic and Asymptomatic)	112 per 1,000	**82 per 1,000** (36 to 175)	**OR 0.71** (0.30 to 1.68)	246 (1 RCT)	⨁⨁◯◯ Low[a,b,c]	
Peripheral Edema	144 per 1,000	**182 per 1,000** (101 to 305)	**OR 1.32** (0.67 to 2.61)	246 (1 RCT)	⨁⨁◯◯ Low[a,b,c]	

***The risk in the intervention group** (and its 95% confidence interval) is based on the assumed risk in the comparison group and the **relative effect** of the intervention (and its 95% CI).
CI: confidence interval; **OR:** odds ratio.

Explanations
a. This study does not provide details regarding sequence generation, and investigators, and subjects were not blind to exposure assignment. Selective outcome reporting may also have occurred.
b. A 95% CI contains the potential for appreciable benefit and harm.
c. Small sample with few events.

Table S9.11. Pramipexole compared to rotigotine for patients with Parkinson's disease

Patient or population: patients with Parkinson's disease
Intervention: pramipexole
Comparison: rotigotine

Outcomes	Anticipated absolute effects* (95% CI)		Relative effect (95% CI)	№ of participants (studies)	Certainty of the evidence (GRADE)	Comments
	**Risk with rotigotine**	**Risk with pramipexole**				
Orthostatic Hypotension (Symptomatic and Asymptomatic)	34 per 1,000	**50 per 1,000** (19 to 123)	**OR 1.47** (0.55 to 3.95)	405 (1 RCT)	⊕⊕⊕◯ Moderate[a,b]	

***The risk in the intervention group** (and its 95% confidence interval) is based on the assumed risk in the comparison group and the **relative effect** of the intervention (and its 95% CI).
CI: confidence interval; **OR:** odds ratio.

Explanations
a. A 95% CI contains the potential for appreciable harm, as well as possible benefit.
b. Small sample with few events.

Table S9.12. Pramipexole compared to rasagiline for patients with Parkinson's disease

Patient or population: patients with Parkinson's disease
Intervention: pramipexole
Comparison: rasagiline

Outcomes	Anticipated absolute effects* (95% CI)		Relative effect (95% CI)	№ of participants (studies)	Certainty of the evidence (GRADE)	Comments
	**Risk with rasagiline**	**Risk with pramipexole**				
Syncope	38 per 1,000	**107 per 1,000** (23 to 384)	**OR 3.06** (0.59 to 15.89)	109 (1 RCT)	⊕◯◯◯ Very low[a,b,c]	
Orthostatic Hypotension (Symptomatic and Asymptomatic)	19 per 1,000	**54 per 1,000** (6 to 360)	**OR 2.94** (0.30 to 29.22)	109 (1 RCT)	⊕◯◯◯ Very low[a,b,c]	

***The risk in the intervention group** (and its 95% confidence interval) is based on the assumed risk in the comparison group and the **relative effect** of the intervention (and its 95% CI).
CI: confidence interval; **OR:** odds ratio.

Explanations
a. This study provides limited or no information about sequence generation; allocation concealment; and blinding of participants, personnel, and outcome assessors. Selective outcome reporting may also have occurred.
b. A 95% CI contains the potential for appreciable harm, as well as possible benefit.
c. Small sample with few events.

Table S9.13. Pramipexole compared to all other dopamine agonists for patients with Parkinson's disease

Patient or population: patients with Parkinson's disease
Intervention: pramipexole
Comparison: all other dopamine agonists

Outcomes	**Anticipated absolute effects*** (95% CI)		Relative effect (95% CI)	№ of participants (studies)	Certainty of the evidence (GRADE)	Comments
	**Risk with all other dopamine agonists**	**Risk with pramipexole**				
Heart Failure	**Low**		**RR 1.28** (0.82 to 2.00)	149 cases, 1137 controls (1 non-randomized study)	⊕◯◯◯ Very low[a]	
	76 per 1,000	**98 per 1,000** (63 to 153)				

***The risk in the intervention group** (and its 95% confidence interval) is based on the assumed risk in the comparison group and the **relative effect** of the intervention (and its 95% CI).
CI: confidence interval; **RR:** risk ratio.

Explanations
a. A 95% CI contains the potential for harm, as well as some benefit.

Table S9.14. Ropinirole compared to placebo for patients with Parkinson's disease

Patient or population: patients with Parkinson's disease
Intervention: ropinirole
Comparison: placebo

Outcomes	Anticipated absolute effects* (95% CI)		Relative effect (95% CI)	№ of participants (studies)	Certainty of the evidence (GRADE)	Comments
	**Risk with placebo**	**Risk with ropinirole**				
Orthostatic Hypotension	27 per 1,000	**55 per 1,000** (20 to 142)	**OR 2.10** (0.73 to 6.02)	691 (3 RCTs)	⊕⊕⊕◯ Moderate[a,b]	
Syncope	0 per 1,000	**0 per 1,000** (0 to 0)	**OR 2.85** (0.12 to 70.42)	393 (1 RCT)	⊕⊕◯◯ Low[a,c]	
Valvulopathy	0 per 1,000	**0 per 1,000** (0 to 0)	not estimable	252 (1 RCT)	⊕⊕◯◯ Low[a,d]	
Peripheral Edema	35 per 1,000	**12 per 1,000** (2 to 69)	**OR 0.33** (0.05 to 2.02)	252 (1 RCT)	⊕⊕◯◯ Low[a,b,d]	
Heart Failure	**Low**		**OR 1.04** (0.87 to 1.24)	3101 cases, 14408 controls (2 non-randomized studies)	⊕◯◯◯ Very low[e]	
	46 per 1,000	**48 per 1,000** (40 to 56)				

***The risk in the intervention group** (and its 95% confidence interval) is based on the assumed risk in the comparison group and the **relative effect** of the intervention (and its 95% CI).
CI: confidence interval; **OR:** odds ratio.

Explanations
a. Small sample with few events;
b. A 95% CI includes the potential for appreciable benefit, as well as harm;
c. This study provides limited or no information about the blinding of participants, personnel, and outcome assessors. Selective outcome reporting may also have occurred;
d. This study provides limited or no information about the blinding of participants, personnel, and outcome assessors;
e. Serious concerns for imprecision because 95% CI includes the potential for small harm, as well as small benefit.

Table S9.15. Ropinirole compared to no use of non-ergot dopamine agonists for patients with Parkinson's disease

Patient or population: patients with Parkinson's disease
Intervention: ropinirole
Comparison: no use of non-ergot dopamine agonists

Outcomes	**Anticipated absolute effects*** (95% CI)		Relative effect (95% CI)	№ of participants (studies)	Certainty of the evidence (GRADE)	Comments
	**Risk with no use of non-ergot dopamine agonists**	**Risk with ropinirole**				
Hypotension	**Low**		**OR 1.02** (0.79 to 1.32)	869 cases, 2607 controls (1 non-randomized study)	⊕◯◯◯ Very low[a]	
	3,000 per 100,000	**3058 per 100,000** (2,385 to 3,922)				
Myocardial Infarction	**Low**		**OR 0.98** (0.68 to 1.42)	482 cases, 01446 controls (1 non-randomized study)	⊕◯◯◯ Very low[a]	
	30 per 1,000	**29 per 1,000** (21 to 42)				
Stroke	**Low**		**OR 0.67** (0.41 to 1.09)	478 cases, 01434 controls (1 non-randomized study)	⊕◯◯◯ Very low[a]	
	30 per 1,000	**20 per 1,000** (13 to 33)				
Valvulopathy	**Low**		**OR 1.08** (0.82 to 1.43)	981 cases, 02943 controls (1 non-randomized study)	⊕◯◯◯ Very low[a]	
	30 per 1,000	**32 per 1,000** (25 to 42)				

***The risk in the intervention group** (and its 95% confidence interval) is based on the assumed risk in the comparison group and the **relative effect** of the intervention (and its 95% CI).
CI: confidence interval; **OR:** odds ratio.

Explanations
a. A 95% CI includes the potential for potential benefit, as well as potential harm.

Table S9.16. Ropinirole immediate release (IR) compared to ropinirole prolonged release (PR) for patients with Parkinson's disease

Patient or population: patients with Parkinson's disease
Intervention: ropinirole immediate release (IR)
Comparison: ropinirole prolonged release (PR)

Outcomes	Anticipated absolute effects* (95% CI)		Relative effect (95% CI)	№ of participants (studies)	Certainty of the evidence (GRADE)	Comments
	**Risk with ropinirole prolonged release (PR)**	**Risk with ropinirole immediate release (IR)**				
Orthostatic Hypotension (Symptomatic and Asymptomatic)	17 per 1,000	**52 per 1,000** (14 to 171)	**OR 3.18** (0.85 to 11.96)	350 (1 RCT)	⊕⊕◯◯ Low[a,b,c]	
Hypotension	45 per 1,000	**23 per 1,000** (7 to 74)	**OR 0.50** (0.15 to 1.69)	350 (1 RCT)	⊕⊕◯◯ Low[a,c,d]	

***The risk in the intervention group** (and its 95% confidence interval) is based on the assumed risk in the comparison group and the **relative effect** of the intervention (and its 95% CI).
CI: confidence interval; **OR:** odds ratio.

Explanations
a. This study provides limited or no information about allocation concealment, as well as the blinding of participants, personnel, and outcome assessors;
b. A 95% CI includes potential for appreciable harm, as well as possible benefit;
c. Small sample with few events;
d. A 95% CI includes potential for possible harm and benefit.

Table S9.17. Ropinirole prolonged release (PR) compared to placebo for patients with Parkinson's disease

Patient or population: patients with Parkinson's disease
Intervention: ropinirole prolonged release (PR)
Comparison: placebo

Outcomes	**Anticipated absolute effects**[*] (95% CI)		Relative effect (95% CI)	№ of participants (studies)	Certainty of the evidence (GRADE)	Comments
	**Risk with placebo**	**Risk with ropinirole prolonged release (PR)**				
Orthostatic Hypotension (Symptomatic and Asymptomatic)	41 per 1,000	**34 per 1,000** (11 to 97)	**OR 0.83** (0.27 to 2.51)	345 (1 RCT)	⊕⊕◯◯ Low[a,b,c]	
Hypotension	6 per 1,000	**23 per 1,000** (3 to 175)	**OR 3.95** (0.44 to 35.73)	345 (1 RCT)	⊕⊕◯◯ Low[a,b,c]	
Peripheral Edema	24 per 1,000	**29 per 1,000** (8 to 100)	**OR 1.22** (0.32 to 4.62)	345 (1 RCT)	⊕⊕◯◯ Low[a,b,c]	
Hypertension	33 per 1,000	**4 per 1,000** (1 to 36)	**OR 0.13** (0.02 to 1.10)	695 (2 RCTs)	⊕⊕◯◯ Low[b,d]	
Arrhythmia	6 per 1,000	**6 per 1,000** (0 to 85)	**OR 0.97** (0.06 to 15.65)	345 (1 RCT)	⊕⊕◯◯ Low[a,b,c]	

*__The risk in the intervention group__ (and its 95% confidence interval) is based on the assumed risk in the comparison group and the **relative effect** of the intervention (and its 95% CI).
CI: confidence interval; **OR:** odds ratio.

Explanations
a. This study provides limited or no information about sequence generation, as well as the blinding of participants, personnel, and outcome assessors;
b. A 95% CI contains the potential for both harm, as well as benefit;
c. Small sample with few events;
d. The studies provide limited or no information on blinding of participants, personnel, and outcome assessors.

Table S9.18. Ropinirole compared to bromocriptine for patients with Parkinson's disease

Patient or population: patients with Parkinson's disease
Intervention: ropinirole
Comparison: bromocriptine

Outcomes	**Anticipated absolute effects*** (95% CI)		Relative effect (95% CI)	№ of participants (studies)	Certainty of the evidence (GRADE)	Comments
	**Risk with bromocriptine**	**Risk with ropinirole**				
Orthostatic Hypotension (Symptomatic and Asymptomatic)	112 per 1,000	**82 per 1,000** (44 to 148)	**OR 0.71** (0.37 to 1.38)	411 (2 RCTs)	⊕⊕◯◯ Low[a,b]	
Heart Failure	30 per 1,000	**12 per 1,000** (2 to 59)	**OR 0.39** (0.07 to 2.04)	335 (1 RCT)	⊕⊕◯◯ Low[a,b]	
Myocardial Infarction	24 per 1,000	**6 per 1,000** (1 to 51)	**OR 0.24** (0.03 to 2.21)	335 (1 RCT)	⊕⊕◯◯ Low[a,b]	

***The risk in the intervention group** (and its 95% confidence interval) is based on the assumed risk in the comparison group and the **relative effect** of the intervention (and its 95% CI).
CI: confidence interval; **OR:** odds ratio.

Explanations
a. This study [15] provides limited or no information about sequence generation; allocation concealment; and blinding of participants, personnel, and outcome assessors. Selective outcome reporting may also have occurred;
b. Serious concerns for imprecision because 95% CI includes the potential for both harm and benefit, and it had a small sample with few events.

Table S9.19. Ropinirole compared to levodopa for patients with Parkinson's disease

Patient or population: patients with Parkinson's disease
Intervention: ropinirole
Comparison: levodopa

Outcomes	**Anticipated absolute effects**[*] (95% CI)		Relative effect (95% CI)	№ of participants (studies)	Certainty of the evidence (GRADE)	Comments
	**Risk with levodopa**	**Risk with ropinirole**				
Syncope	11 per 1,000	**11 per 1,000** (1 to 112)	**OR 0.99** (0.09 to 11.12)	268 (1 RCT)	⨁⨁◯◯ Low[a,b,c]	
Orthostatic Hypotension (Symptomatic and Asymptomatic)	117 per 1,000	**111 per 1,000** (54 to 214)	**OR 0.94** (0.43 to 2.05)	268 (1 RCT)	⨁⨁⨁◯ Moderate[b,c]	
Heart Failure	**Low**		**OR 1.02** (0.76 to 1.37)	1954 cases, 29042 controls (2 non-randomized studies)	⨁◯◯◯ Very low[b,d]	
	149 per 1,000	**152 per 1,000** (117 to 193)				
Hypotension	**Low**		**OR 0.81** (0.49 to 1.35)	545 cases, 1684 controls (1 non-randomized study)	⨁◯◯◯ Very low[b]	
	31 per 1,000	**25 per 1,000** (15 to 41)				
Myocardial Infarction	**Low**		**OR 1.09** (0.52 to 2.29)	323 cases, 929 controls (1 non-randomized study)	⨁◯◯◯ Very low[b]	
	29 per 1,000	**31 per 1,000** (15 to 64)				
Stroke	**Low**		**OR 0.52** (0.20 to 1.36)	333 cases, 914 controls (1 non-randomized study)	⨁◯◯◯ Very low[b]	
	28 per 1,000	**14 per 1,000** (6 to 37)				
Valvulopathy	**Low**					

Table S9.19. Ropinirole compared to levodopa for patients with Parkinson's disease

Patient or population: patients with Parkinson's disease
Intervention: ropinirole
Comparison: levodopa

Outcomes	**Anticipated absolute effects*** (95% CI)		Relative effect (95% CI)	№ of participants (studies)	Certainty of the evidence (GRADE)	Comments
	**Risk with levodopa**	**Risk with ropinirole**				
	30 per 1,000	**51 per 1,000** (29 to 87)	**OR 1.73** (0.98 to 3.08)	652 cases, 1961 controls (1 non-randomized study)	⊕◯◯◯ Very low[b]	

***The risk in the intervention group** (and its 95% confidence interval) is based on the assumed risk in the comparison group and the **relative effect** of the intervention (and its 95% CI).
CI: confidence interval; **OR:** odds ratio.

Explanations
a. This study [28] provides limited or no information about sequence generation and treatment allocation was not concealed. Selective outcome reporting may also have occurred;
b. Serious concerns for imprecision because 95% CI includes the potential for appreciable harm, as well as some benefit;
c. Small sample with few events;
d. Study populations [6,20] include both past and current users of levodopa.

Table S9.20. Rotigotine compared to placebo for patients with Parkinson's disease

Patient or population: patients with Parkinson's disease
Setting: outpatient
Intervention: rotigotine
Comparison: placebo

Outcomes	Anticipated absolute effects* (95% CI)		Relative effect (95% CI)	№ of participants (studies)	Certainty of the evidence (GRADE)	Comments
	**Risk with placebo**	**Risk with rotigotine**				
Peripheral Edema	22 per 1,000	**30 per 1,000** (13 to 67)	**OR 1.38** (0.59 to 3.22)	1634 (5 RCTs)	⊕⊕⊕◯ Moderate[a,b]	
Orthostatic Hypotension (Symptomatic and Asymptomatic)	55 per 1,000	**22 per 1,000** (13 to 38)	**OR 0.39** (0.22 to 0.68)	1697 (5 RCTs)	⊕⊕⊕◯ Moderate[c]	
Orthostatic Hypotension (Symptomatic)	10 per 1,000	**5 per 1,000** (0 to 73)	**OR 0.48** (0.03 to 7.76)	304 (1 RCT)	⊕⊕⊕◯ Moderate[a,d]	
Valvulopathy	0 per 1,000	**0 per 1,000** (0 to 0)	not estimable	253 (1 RCT)	⊕⊕◯◯ Low[d,e,f]	

***The risk in the intervention group** (and its 95% confidence interval) is based on the assumed risk in the comparison group and the **relative effect** of the intervention (and its 95% CI).
CI: confidence interval; **OR:** odds ratio.

Explanations
a. A 95% CI contains the potential for benefit as well as the potential for harm;
b. Few events reported;
c. Unclear as to whether blinding of participants, personnel, and outcome assessors occurred in two (Watts, 2007 & Mizuno, 2014) of the five studies. Additionally, sequence generation was unclear in a single study [40];
d. Small sample size with few events;
e. This study provides limited or no information about the blinding of participants, personnel, and outcome assessors;
f. No events reported in either the treatment or placebo group.

Table S9.21. Rotigotine compared to ropinirole for patients with Parkinson's disease

Patient or population: patients with Parkinson's disease
Intervention: rotigotine
Comparison: ropinirole

Outcomes	Anticipated absolute effects* (95% CI)		Relative effect (95% CI)	№ of participants (studies)	Certainty of the evidence (GRADE)	Comments
	**Risk with ropinirole**	**Risk with rotigotine**				
Valvulopathy	0 per 1,000	**0 per 1,000** (0 to 0)	not estimable	335 (1 RCT)	⊕⊕◯◯ Low[a,b]	
Orthostatic Hypotension (Symptomatic and Asymptomatic)	42 per 1,000	**30 per 1,000** (10 to 90)	**OR 0.70** (0.22 to 2.25)	335 (1 RCT)	⊕⊕◯◯ Low[a,c,d]	
Peripheral Edema	12 per 1,000	**2 per 1,000** (0 to 48)	**OR 0.20** (0.01 to 4.12)	335 (1 RCT)	⊕⊕◯◯ Low[a,c,d]	

***The risk in the intervention group** (and its 95% confidence interval) is based on the assumed risk in the comparison group and the **relative effect** of the intervention (and its 95% CI).
CI: confidence interval; **OR:** odds ratio.

Explanations
a. This study [19] provides limited or no information about the blinding of participants, personnel, and outcome assessors;
b. No events reported;
c. A 95% CI includes the potential for possible harm, as well as possible benefit;
d. Small sample size with few events.

Table S9.22. Apomorphine compared to placebo for patients with Parkinson's disease

Patient or population: patients with Parkinson's disease
Intervention: apomorphine
Comparison: placebo

Outcomes	**Anticipated absolute effects*** (95% CI)		Relative effect (95% CI)	№ of participants (studies)	Certainty of the evidence (GRADE)	Comments
	**Risk with placebo**	**Risk with apomorphine**				
Hypertension	0 per 1,000	**0 per 1,000** (0 to 0)	**OR 4.80** (0.25 to 92.31)	55 (1 RCT)	⊕◯◯◯ Very low[a,b,c]	
Hypotension	0 per 1,000	**0 per 1,000** (0 to 0)	**OR 2.46** (0.26 to 22.91)	161 (2 RCTs)	⊕◯◯◯ Very low[a,b,c,d]	

*__The risk in the intervention group__ (and its 95% confidence interval) is based on the assumed risk in the comparison group and the **relative effect** of the intervention (and its 95% CI).
CI: confidence interval; **OR:** odds ratio.

Explanations
a. The study [7] did not report on allocation concealment, nor on blinding of participants, personnel, and outcomes assessors. Selective outcomes may have occurred;
b. A 95% CI includes the potential for unknown harm and benefit;
c. Small sample size with very few events;
d. The study [13] was rated to have serious risk of bias for blinding of participants and assessors, as well as for attrition bias.

Table S9.23. Piribedil compared to placebo for patients with Parkinson's disease

Patient or population: patients with Parkinson's disease
Intervention: piribedil
Comparison: placebo

Outcomes	**Anticipated absolute effects*** (95% CI)		Relative effect (95% CI)	№ of participants (studies)	Certainty of the evidence (GRADE)	Comments
	**Risk with placebo**	**Risk with piribedil**				
Orthostatic Hypotension (Symptomatic and Asymptomatic)	35 per 1,000	**55 per 1,000** (24 to 120)	**OR 1.61** (0.68 to 3.80)	520 (2 RCTs)	⊕⊕⊕◯ Moderate[a]	
Hypertension	44 per 1,000	**95 per 1,000** (44 to 192)	**OR 2.29** (1.01 to 5.18)	405 (1 RCT)	⊕⊕⊕⊕ High	
Peripheral Edema	50 per 1,000	**73 per 1,000** (29 to 174)	**OR 1.49** (0.56 to 3.99)	405 (1 RCT)	⊕⊕⊕◯ Moderate[a,b]	

***The risk in the intervention group** (and its 95% confidence interval) is based on the assumed risk in the comparison group and the **relative effect** of the intervention (and its 95% CI).
CI: confidence interval; **OR:** odds ratio.

Explanations
a. A 95% CI contains the potential for appreciable harm, as well as possible benefit;
b. Small number of events.

Table S9.24. Piribedil compared to bromocriptine for patients with Parkinson's disease

Patient or population: patients with Parkinson's disease
Intervention: piribedil
Comparison: bromocriptine

Outcomes	**Anticipated absolute effects*** (95% CI)		Relative effect (95% CI)	№ of participants (studies)	Certainty of the evidence (GRADE)	Comments
	**Risk with bromocriptine**	**Risk with piribedil**				
Syncope	37 per 1,000	**24 per 1,000** (8 to 70)	**OR 0.63** (0.20 to 1.96)	425 (1 RCT)	⊕⊕◯◯ Low[a,b,c]	
Hypertension	42 per 1,000	**71 per 1,000** (32 to 153)	**OR 1.76** (0.75 to 4.12)	425 (1 RCT)	⊕⊕◯◯ Low[a,b,c]	
Hypotension	93 per 1,000	**76 per 1,000** (39 to 141)	**OR 0.8** (0.4 to 1.6)	425 (1 RCT)	⊕⊕◯◯ Low[a,b,c]	
Peripheral Edema	47 per 1,000	**47 per 1,000** (20 to 109)	**OR 1.02** (0.42 to 2.52)	425 (1 RCT)	⊕⊕◯◯ Low[a,b,c]	

***The risk in the intervention group** (and its 95% confidence interval) is based on the assumed risk in the comparison group and the **relative effect** of the intervention (and its 95% CI).
CI: confidence interval; **OR:** odds ratio.

Explanations

a. While randomized, this study [5] did not report on sequence generation and allocation concealment. Additionally, it is unclear as to whether participants, personnel, and outcomes assessors were blinded to exposure assignment;
b. A 95% CI includes the potential for appreciable harm, as well as possible benefit;
c. Small number of events.

References

1. Guyatt, G.H.; Oxman, A.D.; Schunemann, H.J.; Tugwell, P.; Knottnerus, A. GRADE guidelines: a new series of articles in the Journal of Clinical Epidemiology. *J Clin Epidemiol* **2011**, *64*, 380-382, doi:10.1016/j.jclinepi.2010.09.011.
2. Schunemann, H.J.; Oxman, A.D.; Brozek, J.; Glasziou, P.; Jaeschke, R.; Vist, G.E.; Williams, J.W., Jr.; Kunz, R.; Craig, J.; Montori, V.M.; et al. Grading quality of evidence and strength of recommendations for diagnostic tests and strategies. *BMJ* **2008**, *336*, 1106-1110, doi:10.1136/bmj.39500.677199.AE.
3. Barone, P.; Poewe, W.; Albrecht, S.; Debieuvre, C.; Massey, D.; Rascol, O.; Tolosa, E.; Weintraub, D. Pramipexole for the treatment of depressive symptoms in patients with Parkinson's disease: a randomised, double-blind, placebo-controlled trial. *Lancet Neurol* **2010**, *9*, 573-580, doi:10.1016/S1474-4422(10)70106-X.
4. Blindeauer, K. A Controlled Trial of Rotigotine Monotherapy in Early Parkinson's Disease. *Archives of Neurology* **2003**, *60(12)*, 1721-1728, doi:http://dx.doi.org/10.1001/archneur.60.12.1721.
5. Castro-Caldas, A.; Delwaide, P.; Jost, W.; Merello, M.; Williams, A.; Lamberti, P.; Aguilar, M.; Del Signore, S.; Cesaro, P.; Bueri, J.; et al. The Parkinson-control study: A 1-year randomize, double-blind trial comapring piribedil (150 mg/day) with bromocriptine (25 mg/day) in early combination with levodopa in Parkinson's disease. *Movement Disorders* **2006**, *21(4)*, 500-509, doi:http://dx.doi.org/10.1002/mds.20750.
6. Crispo, J.A.G.; Willis, A.W.; Thibault, D.P.; Fortin, Y.; Emons, M.; Bjerre, L.M.; Kohen, D.E.; Perez-Lloret, S.; Mattison, D.; Krewski, D. Associations Between Cardiovascular Events and Nonergot Dopamine Agonists in Parkinson's Disease. *Movement Disorders Clinical Practice* **2016**, *3*, 257-267, doi:https://dx.doi.org/10.1002/mdc3.12286.
7. Grosset, K.A.; Malek, N.; Morgan, F.; Grosset, D.G. Inhaled apomorphine in patients with 'on-off' fluctuations: A randomized, double-blind, placebo-controlled, clinic and home based, parallel-group study. *Journal of Parkinson's Disease* **2013**, *3(1)*, 31-37, doi:http://dx.doi.org/10.3233/JPD-120142.
8. Guttman, M. Double-blind comparison of pramipexole and bromocriptine treatment with placebo in advanced Parkinson's disease. *Neurology* **1997**, *49(4)*, 1060-1065, doi:http://dx.doi.org/10.1212/WNL.49.4.1060.
9. Holloway, R.G.; Shoulson, I.; Fahn, S.; Kieburtz, K.; Lang, A.; Marek, K.; McDermott, M.; Seibyl, J.; Weiner, W.; Musch, B.; et al. Pramipexole vs levodopa as initial treatment for Parkinson disease: a 4-year randomized controlled trial. *Archives of Neurology* **2004**, *61*, 1044-1053.
10. Hubble, J.P.; Koller, W.C.; Cutler, N.R.; Sramek, J.J.; Friedman, J.; Goetz, C.; Ranhosky, A.; Korts, D.; Elvin, A. Pramipexole in patients with early Parkinson's disease. *Clinical Neuropharmacology* **1995**, *18*, 338-347.
11. Im, J.H.; Ha, J.H.; Cho, I.S.; Lee, M.C. Ropinirole as an adjunct to levodopa in the treatment of Parkinson's disease: A 16-week bromocriptine controlled study. *Journal of Neurology* **2003**, *250(1)*, 90-96, doi:http://dx.doi.org/10.1007/s00415-003-0937-z.
12. Junghanns, S.; Fuhrmann, J.T.; Simonis, G.; Oelwein, C.; Koch, R.; Strasser, R.H.; Reichmann, H.; Storch, A. Valvular heart disease in Parkinson's disease patients treated with dopamine agonists: a reader-blinded monocenter echocardiography study. *Mov Disord* **2007**, *22*, 234-238, doi:10.1002/mds.21225.
13. Katzenschlager, R.; Poewe, W.; Rascol, O.; Trenkwalder, C.; Deuschl, G.; Chaudhuri, K.R.; Henriksen, T.; van Laar, T.; Spivey, K.; Vel, S.; et al. Apomorphine subcutaneous infusion in patients with Parkinson's disease with persistent motor fluctuations (TOLEDO): a multicentre,

double-blind, randomised, placebo-controlled trial. *The Lancet Neurology* **2018**, *17(9)*, 749-759, doi:http://dx.doi.org/10.1016/S1474-4422%2818%2930239-4.

14. Kieburtz, K. Twice-daily, low-dose pramipexole in early Parkinson's disease: A randomized, placebo-controlled trial. *Movement Disorders* **2011**, *26(1)*, 37-44, doi:http://dx.doi.org/10.1002/mds.23396.
15. Korczyn, A.D.; Brunt, E.R.; Larsen, J.P.; Nagy, Z.; Poewe, W.H.; Ruggieri, S. A 3-year randomized trial of ropinirole and bromocriptine in early Parkinson's disease. The 053 Study Group. *Neurology* **1999**, *53*, 364-370, doi:10.1212/wnl.53.2.364.
16. LeWitt, P.A.; Lyons, K.E.; Pahwa, R.; LeWitt, P.A.; Lyons, K.E.; Pahwa, R. Advanced Parkinson disease treated with rotigotine transdermal system: PREFER Study. *Neurology* **2007**, *68*, 1262-1267, doi:10.1212/01.wnl.0000259516.61938.bb.
17. Lieberman, A.; Ranhosky, A.; Korts, D. Clinical evaluation of pramipexole in advanced Parkinson's disease: Results of a double-blind, placebo-controlled, parallel-group study. *Neurology* **1997**, *49(1)*, 162-168, doi:http://dx.doi.org/10.1212/WNL.49.1.162.
18. Mizuno, Y.; Yamamoto, M.; Kuno, S.; Hasegawa, K.; Hattori, N.; Kagimura, T.; Sarashina, A.; Rascol, O.; Schapira, A.H.V.; Barone, P.; et al. Efficacy and safety of extended-versus immediate-release pramipexole in Japanese patients with advanced and L-dopa-undertreated Parkinson disease: A double-blind, randomized trial. *Clinical Neuropharmacology* **2012**, *35(4)*, 174-181, doi:http://dx.doi.org/10.1097/WNF.0b013e31825f77b9.
19. Mizuno, Y.; Nomoto, M.; Hasegawa, K.; Hattori, N.; Kondo, T.; Murata, M.; Takeuchi, M.; Takahashi, M.; Tomida, T. Rotigotine vs ropinirole in advanced stage Parkinson's disease: Adouble-blind study. *Parkinsonism and Related Disorders* **2014**, *20(12)*, 1388-1393, doi:http://dx.doi.org/10.1016/j.parkreldis.2014.10.005.
20. Mokhles, M.M.; Trifiro, G.; Dieleman, J.P.; Haag, M.D.; van Soest, E.M.; Verhamme, K.M.; Mazzaglia, G.; Herings, R.; Luise, C.; Ross, D.; et al. The risk of new onset heart failure associated with dopamine agonist use in Parkinson's disease. *Pharmacological Research* **2012**, *65*, 358-364, doi:https://dx.doi.org/10.1016/j.phrs.2011.11.009.
21. Moller, J.C.; Oertel, W.H.; Koster, J.; Pezzoli, G.; Provinciali, L. Long-term efficacy and safety of pramipexole in advanced Parkinson's disease: Results from a European multicenter trial. *Movement Disorders* **2005**, *20(5)*, 602-610, doi:http://dx.doi.org/10.1002/mds.20397.
22. Navan, P.; Findley, L.J.; Jeffs, J.A.R.; Pearce, R.K.B.; Bain, P.G. Randomized, double-blind, 3-month parallel study of the effects of pramipexole, pergolide, and placebo on Parkinsonian tremor. *Movement Disorders* **2003**, *18(11)*, 1324-1331, doi:http://dx.doi.org/10.1002/mds.10538.
23. Nicholas, A.P.; Borgohain, R.; Chana, P.; Surmann, E.; Thompson, E.L.; Bauer, L.; Whitesides, J.; Elmer, L.W. A randomized study of rotigotine dose response on 'off' time in advanced Parkinson's disease. *Journal of Parkinson's disease* **2014**, *4(3)*, 361-373, doi:http://dx.doi.org/10.3233/JPD-130320.
24. Pahwa, R.; Stacy, M.A.; Factor, S.A.; Lyons, K.E.; Stocchi, F.; Hersh, B.P.; Elmer, L.W.; Truong, D.D.; Earl, N.L. Ropinirole 24-hour prolonged release: Randomized, controlled study in advanced Parkinson disease. *Neurology* **2007**, *68(14)*, 1108-1115, doi:http://dx.doi.org/10.1212/01.wnl.0000258660.74391.c1.
25. Peralta, C.; Wolf, E.; Alber, H.; Seppi, K.; Muller, S.; Bosch, S.; Wenning, G.K.; Pachinger, O.; Poewe, W. Valvular heart disease in Parkinson's disease vs. controls: An echocardiographic study. *Movement Disorders* **2006**, *21*, 1109-1113.
26. Poewe, W.H.; Rascol, O.; Quinn, N.; Tolosa, E.; Oertel, W.H.; Martignoni, E.; Rupp, M.; Boroojerdi, B. Efficacy of pramipexole and transdermal rotigotine in advanced Parkinson's disease: a double-blind, double-dummy, randomised controlled trial. *Lancet Neurology* **2007**, *6(6)*, 513-520, doi:http://dx.doi.org/10.1016/S1474-4422%2807%2970108-4.

27. Rascol, O.; Lees, A.J.; Senard, J.M.; Pirtosek, Z.; Montastruc, J.L.; Fuell, D. Ropinirole in the treatment of levodopa-induced motor fluctuations in patients with Parkinson's disease. *Clinical Neuropharmacology* **1996**, *19(3)*, 234-245, doi:http://dx.doi.org/10.1097/00002826-199619030-00005.
28. Rascol, O.; Brooks, D.J.; Brunt, E.R.; Korczyn, A.D.; Poewe, W.H.; Stocchi, F. Ropinirole in the treatment of early Parkinson's disease: A 6-month interim report of a 5-year levodopa-controlled study. *Movement Disorders* **1998**, *13(1)*, 39-45, doi:http://dx.doi.org/10.1002/mds.870130111.
29. Rascol, O.; Brooks, D.J.; Korczyn, A.D.; De Deyn, P.P.; Clarke, C.E.; Lang, A.E. A five-year study of the incidence of dyskinesia in patients with early Parkinson's disease who were treated with ropinirole or levodopa. *New England Journal of Medicine* **2000**, *342*, 1484-1491.
30. Rascol, O.; Dubois, B.; Castro Caldas, A.; Senn, S.; Del Signore, S.; Lees, A. Early piribedil monotherapy of parkinson's disease: A planned seven-month report of the REGAIN study. *Movement Disorders* **2006**, *21(12)*, 2110-2115, doi:http://dx.doi.org/10.1002/mds.21122.
31. Renoux, C.; Dell'Aniello, S.; Brophy, J.M.; Suissa, S. Dopamine agonist use and the risk of heart failure. *Pharmacoepidemiol Drug Saf* **2012**, *21*, 34-41, doi:https://dx.doi.org/10.1002/pds.2267.
32. Sampaio, C.; Bronzova, J.; Hauser, R.A.; Lang, A.E.; Rascol, O.; van de Witte, S.V.; Theeuwes, A. Pardoprunox in early Parkinson's disease: Results from 2 large, randomized double-blind trials. *Movement Disorders* **2011**, *26(8)*, 1464-1476, doi:http://dx.doi.org/10.1002/mds.23590.
33. Schapira, A.H.V.; Barone, P.; Hauser, R.A.; Mizuno, Y.; Rascol, O.; Busse, M.; Salin, L.; Juhel, N.; Poewe, W. Extended-release pramipexole in advanced Parkinson disease: A randomized controlled trial. *Neurology* **2011**, *77(8)*, 767-774, doi:http://dx.doi.org/10.1212/WNL.0b013e31822affdb.
34. Schapira, A.H.V.; McDermott, M.P.; Barone, P.; Comella, C.L.; Albrecht, S.; Hsu, H.H.; Massey, D.H.; Mizuno, Y.; Poewe, W.; Rascol, O.; et al. Pramipexole in patients with early Parkinson's disease (PROUD): A randomised delayed-start trial. *The Lancet Neurology* **2013**, *12(8)*, 747-755, doi:http://dx.doi.org/10.1016/S1474-4422%2813%2970117-0.
35. Seiple, W.; Jennings, D.; Rosen, R.B.; Borchert, L.; Canale, L.; Fagan, N.; Gordon, M.F. Ophthalmologic baseline characteristics and 2-year ophthalmologic safety profile of pramipexole IR compared with ropinirole IR in patients with early Parkinson's disease. *Parkinson's Disease* **2016**, *2016 (no pagination)*, doi:http://dx.doi.org/10.1155/2016/8298503.
36. Stocchi, F.; Giorgi, L.; Hunter, B.; Schapira, A.H. PREPARED: Comparison of prolonged and immediate release ropinirole in advanced Parkinson's disease. *Movement Disorders* **2011**, *26(7)*, 1259-1265, doi:http://dx.doi.org/10.1002/mds.23498.
37. Titlic, M.; Tonkic, A.; Jukic, I.; Lusic, I.; Dikanovic, M. Side effects of ropinirole in patients with idiopathic Parkinson's disease. *Bratislavske lekarske listy* **2008**, *109(6)*, 273-275.
38. Viallet, F.; Pitel, S.; Lancrenon, S.; Blin, O. Evaluation of the safety and tolerability of rasagiline in the treatment of the early stages of Parkinson's disease. *Current Medical Research and Opinion* **2013**, *29(1)*, 23-31, doi:http://dx.doi.org/10.1185/03007995.2012.752351.
39. Wang, Y.; Sun, S.; Zhu, S.; Liu, C.; Liu, Y.; Di, Q.; Shang, H.; Ren, Y.; Lu, C.; Gordon, M.F.; et al. The efficacy and safety of pramipexole ER versus IR in Chinese patients with Parkinson's disease: A randomized, double-blind, double-dummy, parallel-group study. *Translational Neurodegeneration* **2014**, *3(1) (no pagination)*, doi:http://dx.doi.org/10.1186/2047-9158-3-11.
40. Watts, R.L.; Jankovic, J.; Waters, C.; Rajput, A.; Boroojerdi, B.; Rao, J.; Watts, R.L.; Jankovic, J.; Waters, C.; Rajput, A.; et al. Randomized, blind, controlled trial of transdermal rotigotine in early Parkinson disease. *Neurology* **2007**, *68*, 272-276, doi:10.1212/01.wnl.0000252355.79284.22.
41. Wermuth, L. A double-blind, placebo-controlled, randomized, multi-center study of pramipexole in advanced Parkinson's disease. *European Journal of Neurology* **1998**, *5*, 235-242.

42. Yamamoto, M.; Uesugi, T.; Nakayama, T. Dopamine agonists and cardiac valvulopathy in Parkinson disease: a case-control study. *Neurology* **2006**, *67*, 1225-1229, doi:10.1212/01.wnl.0000238508.68593.1d.
43. Yamashiro, K.; Komine-Kobayashi, M.; Hatano, T.; Urabe, T.; Mochizuki, H.; Hattori, N.; Iwama, Y.; Daida, H.; Sakai, M.; Nakayama, T.; et al. The frequency of cardiac valvular regurgitation in Parkinson's disease. *Mov Disord* **2008**, *23*, 935-941, doi:10.1002/mds.22036.
44. Zesiewicz, T.A.; Chriscoe, S.; Jimenez, T.; Upward, J.; Davy, M.; Vanmeter, S. A randomized, fixed-dose, dose-response study of ropinirole prolonged release in advanced Parkinson's disease. *Neurodegenerative Disease Management* **2017**, *7(1)*, 61-72, doi:http://dx.doi.org/10.2217/nmt-2016-0038.
45. Zhang, Z.; Wang, J.; Zhang, X.; Chen, S.; Wang, Z.; Zhang, B.; Liu, C.; Qu, Q.; Cheng, Y.; Li, J.; et al. The efficacy and safety of ropinirole prolonged release tablets as adjunctive therapy in Chinese subjects with advanced Parkinson's disease: A multicenter, double-blind, randomized, placebo-controlled study. *Parkinsonism and Related Disorders* **2013**, *19(11)*, 1022-1026, doi:http://dx.doi.org/10.1016/j.parkreldis.2013.07.009.
46. Ziegler, M.; Castro-Caldas, A.; Del Signore, S.; Rascol, O. Efficacy of piribedil as early combination to levodopa in patients with stable parkinson's disease: A 6-month, randomized, placebo-controlled study. *Movement Disorders* **2003**, *18(4)*, 418-425, doi:http://dx.doi.org/10.1002/mds.10359.
47. Barzola, D.; Perez Akly, M.; Diaz, R.; Alvarez, F.; Esnaola, M.M.; Peralta, C. Treatment in Parkinson's disease patients aged 65 and older: A hospital-based cohort. *Movement Disorders* **2012**, *1)*, S38, doi:http://dx.doi.org/10.1002/mds.25051.
48. Bostan, S.; Celik, F.D.; Ozkan, S. Frequency and risk factors of peripheral edema as a side effect of dopamine agonist treatment. *Movement Disorders Clinical Practice* **2020**, *7(Supplement 2)*, S15, doi:http://dx.doi.org/10.1002/mdc3.12923.
49. Chiang, H.L.; Chang, F.; Tsui, D.; Tai, Y.; Ha, A.; Mahant, N.; Griffith, J.; Galea, D.; Kim, S.; Cruse, B.; et al. A prospective comparison of apomorphine, STN deep brain stimulation and levodopa-carbidopa intestinal gel therapy for motor fluctuations in PD. *Movement Disorders* **2017**, *32(Supplement 2)*, 935, doi:http://dx.doi.org/10.1002/mds.27087.
50. Cvetkovska, D.P.; Baneva, N.D. Quality of life in newly diagnosed patients with Parkinson's disease treated with extended-release pramipexole. *European Journal of Neurology* **2012**, *19(SUPPL.1)*, 556, doi:http://dx.doi.org/10.1111/j.1468-1331.2012.03889.x.
51. Dafsari, H.S.; Martinez-Martin, P.; Rizos, A.; Trost, M.; Santos Ghilardi, M.G.; Reddy, P.; Sauerbier, A.; Petry-Schmelzer, J.N.; Kramberger, M.; Borgemeester, R.W.K.; et al. EuroInf 2: Subthalamic stimulation, apomorphine, and levodopa infusion in Parkinson's disease. *Movement Disorders* **2019**, *34*, 353-365, doi:10.1002/mds.27626.
52. Factor, S.; Ondo, W.; Isaacson, S.; Bhargava, P.; Navia, B. A Long-Term Safety, Tolerability, and Efficacy Study of Apomorphine Sublingual Film for On-Demand Treatment of "OFF" Episodes in Patients With Parkinson's Disease: Interim Results. *Movement Disorders* **2020**, *35(SUPPL 1)*, S395, doi:https://dx.doi.org/10.1002/mds.28268.
53. Gehlen, W. Side effects of pharmacotherapy for Parkinson's disease. [German]. *Medizinische Monatsschrift fur Pharmazeuten* **1980**, *3(4)*, 104-110.
54. Gencler, O.S.; Oztekin, N.; Oztekin, M.F. Comparison of Pramipexole Versus Ropinirole in the Treatment of Parkinson's Disease. *Ideggyogyaszati Szemle* **2022**, *75(1-2)*, 39-49, doi:https://dx.doi.org/10.18071/ISZ.75.0023.
55. Hauser, R.A.; Slawek, J.; Barone, P.; Dohin, E.; Surmann, E.; Asgharnejad, M.; Bauer, L. Evaluation of rotigotine transdermal patch for the treatment of apathy and motor symptoms in Parkinson's disease. *BMC Neurology* **2016**, *16*, 1-12, doi:10.1186/s12883-016-0610-7.

56. Isaacson, S.; Espay, A.; Pahwa, R.; Truong, D.; Pappert, E.; Gardzinski, P.; Dzyngel, B.; Agro, A.; Fernandez, H.; Hauser, R. Safety of sublingual apomorphine film (APL-130277) for the treatment of off episodes in patients with Parkinson's disease: Preliminary results from the Phase 3 study dose titration phase. *Journal of the American Geriatrics Society* **2017**, *65(Supplement 1)*, S177-S178, doi:http://dx.doi.org/10.1111/imj.13426.
57. Li, S.H.; Chen, H.B.; Wang, Z.F.; Tang, R.H.; Zhang, X.Y.; Yang, J.S.; Zhao, W.Q.; Sun, X.R.; Ma, J. Efficacy and safety of ropinirole in the treatment of Parkinson's disease: a multi-center, randomized, double-blind and bromocriptine-controlled trial. *Zhonghua yi xue za zhi* **2013**, *Vol.93*, 1952-1957p.
58. Oeda, T.; Masaki, M.; Yamamoto, K.; Mizuta, E.; Kitagawa, N.; Isono, T.; Taniguchi, S.; Doi, K.; Yaku, H.; Yutani, C.; et al. High risk factors for valvular heart disease from dopamine agonists in patients with Parkinson's disease. *Journal of Neural Transmission* **2009**, *116(2)*, 171-178, doi:http://dx.doi.org/10.1007/s00702-008-0160-2.
59. Olanow, C.W.; Factor, S.A.; Espay, A.J.; Hauser, R.A.; Shill, H.A.; Isaacson, S.; Pahwa, R.; Leinonen, M.; Bhargava, P.; Sciarappa, K.; et al. Apomorphine sublingual film for off episodes in Parkinson's disease: a randomised, double-blind, placebo-controlled phase 3 study. *Lancet Neurology* **2020**, *19*, 135-144, doi:10.1016/S1474-4422(19)30396-5.
60. Pahwa, R.; Hauser, R.; Worden, M.; Bhargava, P.; Vakili, G.; Agro, A.; Blum, D.; Olanow, W.; Navia, B. Safety of sublingual apomorphine film (APL-130277) for the treatment of off episodes in patients with Parkinson's disease: Results from a Phase 3 double-blind, placebo-controlled trial. *Movement Disorders* **2018**, *33(Supplement 2)*, S165.
61. Parkinson Study Group. Pramipexole vs levodopa as initial treatment for Parkinson disease: A randomized controlled trial. Parkinson Study Group. *JAMA: Journal of the American Medical Association* **2000**, *284*, 1931-1938, doi:10.1001/jama.284.15.1931.
62. Pinter, M.M.; Pogarell, O.; Oertel, W.H. Efficacy, safety, and tolerance of the non-ergoline dopamine agonist pramipexole in the treatment of advanced Parkinson's disease: a double blind, placebo controlled, randomised, multicentre study. *Journal of Neurology, Neurosurgery & Psychiatry* **1999**, 436-441.
63. Pogarell, O.; Gasser, T.; van Hilten, J.J.; Spieker, S.; Pollentier, S.; Meier, D.; Oertel, W.H. Pramipexole in patients with Parkinson's disease and marked drug resistant tremor: a randomised, double blind, placebo controlled multicentre study. *Journal of Neurology, Neurosurgery & Psychiatry* **2002**, 713-720.
64. Tao, Z.; Chen, J.; Xiao, L.; Liu, C. Pramipexole versus selegiline in patients with Parkinson's disease: An effectiveness and safety (EAS) analysis. *Iranian Red Crescent Medical Journal* **2019**, *21(9) (no pagination)*, doi:http://dx.doi.org/10.5812/IRCMJ.96672.
65. Thijssen, E.; Den Heijer, J.; Puibert, D.; Lei, M.; Hasegawa, D.; Keum, K.; Mochel, K.; Roset, P.; Van Brummelen, E.; Naranda, T.; et al. Randomized, placebo-controlled study investigating the safety, pharmacokinetics and efficacy of inhaled apomorphine in Parkinson's disease patients. *Movement disorders* **2020**, *Vol.35*, S428-p, doi:https://doi.org/10.1002/mds.28268.
66. Wen, H.B.; Zhang, Z.X.; Luo, Y.; Chen, S.D.; Jiang, Y.P.; Zhang, X.Y.; Sun, X.R.; Wang, X.D.; Yao, C. Efficacy of pramipexole in the treatment of Parkinson's disease: A multi-center, randomized, double-blind, bromocriptine-control trial. [Chinese]. *Chinese Journal of Neurology* **2006**, *39(9)*, 604-608.
67. Zhang, Z.-X.; Liu, C.-F.; Tao, E.-X.; Shao, M.; Liu, Y.-M.; Wang, J.; Asgharnejad, M.; Xue, H.-B.; Surmann, E.; Bauer, L. Rotigotine transdermal patch in Chinese patients with advanced Parkinson's disease: A randomized, double-blind, placebo-controlled pivotal study. *Parkinsonism & Related Disorders* **2017**, *44*, 6-12, doi:10.1016/j.parkreldis.2017.08.015.

68. Zhang, Z.-X.; Shang, H.-F.; Hu, X.; Chen, S.; Zhao, Z.; Du, X.; Surmann, E.; Bauer, L.; Asgharnejad, M. Rotigotine transdermal patch in Chinese patients with early Parkinson's disease: A randomized, double-blind, placebo-controlled pivotal study. *Parkinsonism & Related Disorders* **2016**, *28*, 49-55, doi:10.1016/j.parkreldis.2016.04.022.
69. Zheng, B.; Guoqiang, W.; Yi, C. Efficacy of entacapone and pramipexole in treating non-motor symptoms of parkinson's disease: A prospective randomized controlled trial. [Chinese]. *Chinese Journal of Tissue Engineering Research* **2019**, *23(31)*, 5052-5058, doi:http://dx.doi.org/10.3969/j.issn.2095-4344.1422.
